# Transcriptome analysis reveals a ribosome constituents disorder involved in the RPL5 downregulated zebrafish model of Diamond-Blackfan anemia

**DOI:** 10.1186/s12920-016-0174-9

**Published:** 2016-03-09

**Authors:** Yang Wan, Qian Zhang, Zhaojun Zhang, Binfeng Song, Xiaomin Wang, Yingchi Zhang, Qiong Jia, Tao Cheng, Xiaofan Zhu, Anskar Yu-Hung Leung, Weiping Yuan, Haibo Jia, Xiangdong Fang

**Affiliations:** State Key Laboratory of Experimental Hematology, Institute of Hematology and Blood Diseases Hospital, Chinese Academy of Medical Sciences & Peking Union Medical College, Tianjin, 300020 China; CAS Key Laboratory of Genome Sciences and Information, Beijing Institute of Genomics, Chinese Academy of Sciences, Beijing, 100101 China; Key Laboratory of Molecular Biophysics of Ministry of Education, College of Life Science and Technology, Center for Human Genome Research, Huazhong University of Science and Technology, Wuhan, Hubei 430074 China; Department of Medicine, University of Hong Kong, QMH 418 Hong Kong, China

**Keywords:** DBA, RPL5, RNA-seq, ncRNA-seq, Zebrafish

## Abstract

**Background:**

Diamond–Blackfan anemia (DBA) was the first ribosomopathy associated with mutations in ribosome protein (RP) genes. The clinical phenotypes of DBA include failure of erythropoiesis, congenital anomalies and cancer predisposition. Mutations in RPL5 are reported in approximately 9 ~ 21 % of DBA patients, which represents the most common pathological condition related to a large-subunit ribosomal protein. However, it remains unclear how RPL5 downregulation results in severe phenotypes of this disease.

**Results:**

In this study, we generated a zebrafish model of DBA with RPL5 morphants and implemented high-throughput RNA-seq and ncRNA-seq to identify key genes, lncRNAs, and miRNAs during zebrafish development and hematopoiesis. We demonstrated that RPL5 is required for both primitive and definitive hematopoiesis processes. By comparing with other DBA zebrafish models and processing functional coupling network, we identified some common regulated genes, lncRNAs and miRNAs, that might play important roles in development and hematopoiesis.

**Conclusions:**

Ribosome biogenesis and translation process were affected more in RPL5 MO than in other RP MOs. Both P53 dependent (for example, cell cycle pathway) and independent pathways (such as Aminoacyl-tRNA biosynthesis pathway) play important roles in DBA pathology. Our results therefore provide a comprehensive basis for the study of molecular pathogenesis of RPL5*-*mediated DBA and other ribosomopathies.

**Electronic supplementary material:**

The online version of this article (doi:10.1186/s12920-016-0174-9) contains supplementary material, which is available to authorized users.

## Background

Ribosomopathy is defined as a collection of diseases caused by impaired ribosome biogenesis and function. The clinical phenotypes often consist of bone marrow failure and/or craniofacial or other skeletal defects [1]. Diamond-Blackfan anemia (DBA) (MIM 105650) was the first ribosomopathy to be associated with genetic mutations in ribosomal proteins when a mutation in ribosomal protein S19 (*RPS19)* was first reported in 1999 [2]. It is also known as a rare inherited bone marrow failure syndromes (IBMFS) characterized by the failure of erythropoiesis with normal platelet and myeloid lineages. The disease symptoms usually present within the first year of life. Various associated physical malformation are observed in 30–50 % of DBA cases [3], and cancer predisposition has been reported by cohort studies [4]. Corticosteroids, transfusion therapy and stem cell transplantation are the mainstay of treatment [5]. Since the initial identification of RPS19, approximately 50–60 % of DBA patients have been found to have mutations or deletions in genes encoding ribosomal protein (RP) of both the small and large subunits, including RPS24, RPS17, RPL35A, RPL5, RPL11, RPS7, RPS10, RPS26, RPL26 and RPS29 [6, 7].

Ribosomal protein L5 (RPL5) is part of the 60S ribosomal subunit and is localized in both the cytoplasm and nucleus of eukaryotic cells. Acting as a nucleocytoplasmic shuttle protein, it plays an important role in 5S rRNA intracellular transport during assembly of the large ribosomal subunit [8]. Mutations in RPL5 (MIM 603634) have been reported in approximately 5–10 % of DBA patients [6]. Clinical data have shown that mutations in RPL5 are associated with multiple physical abnormalities in DBA, and this was the first ribosomal protein gene to be associated with cleft lip and/or cleft palate abnormalities in DBA patients [9]. RPL5-mutated induced pluripotent stem cells from DBA patients exhibited defective 60S ribosomal subunit assembly, accumulation of 12S pre-rRNA, and impaired erythropoiesis [10].

The pathogenesis of DBA and how ribosomal defects produce a unique but diverse constellation of DBA abnormalities are still not fully understood. While it appears that in the conditions of RP haploinsufficiency caused by mutation of RP genes, RPL5, RPL11 and 5S rRNA bind to human double minute (HDM2), which regulates the proteasome-dependent degradation of P53 [11]. The abnormal activation of p53 pathway may result in accelerated apoptosis [12]. However, in many studies, both P53-dependent and P53-independent pathways have been confirmed to play a role in DBA pathogenesis [13–15].

In addition to mechanisms of gene regulation, functional studies of microRNAs (miRNAs) (19 ~ 24-nucleotide non-coding single-stranded RNA) and long noncoding RNAs (lncRNAs) (exceed 200 nucleotides non-coding single-stranded RNA) during development and hematopoiesis were reported [16, 17]. For example, miR-145 and miR-146a were identified as mediators of the 5q– syndrome phenotype (another ribosomopathy that primarily affects erythropoiesis) [18]. Several studies have indicated that lncRNAs may have spatial and temporal expression with potentially important roles during development and differentiation in zebrafish [19–21]. Some lncRNAs that are dynamically expressed during erythropoiesis are targeted by key erythroid transcription factors GATA binding protein 1 (GATA1), T-cell acute lymphocytic leukemia 1 (TALI), or Kruppel-like factor 1 (KLF1) [22]. However, the majority of lncRNAs remain uncharacterized, and lncRNAs involved in erythropoiesis are just beginning to be defined.

In this study, we generated zebrafish RPL5 morphants and characterized the deregulated mRNAs, ncRNAs and molecular regulatory networks in RPL5-deficient zebrafish embryos in comparison with controls using high-throughput RNA-seq and ncRNA-seq techniques. The RPL5-targeted central nodes of the mRNA regulatory network that we constructed will help to better understand the pathogenesis of DBA.

## Results

### Hematopoiesis and morphological abnormalities in RPL5 downregulation zebrafish

**Fig. 1:**
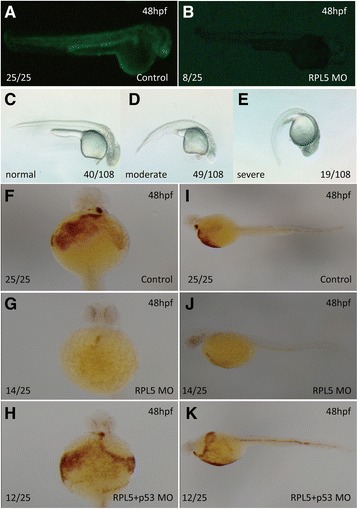
Hemoglobin staining of embryos injected with RPL5 MO and the effectiveness of RPL5 MO. **a**-**b** Embryos co-injected with 25 ng RPL5:*egfp* DNA and 0.25 ng control MO produced green fluorescent protein (**a**), and the expression of the green fluorescent fusion protein was inhibited by co-injection with 0.25 ng RPL5 MO (**b**). **c**-**e** The different phenotype and its ratio of embryos injected with 0.25 ng RPL5 MO **f**-**k** The O-staining results revealed a drastic reduction in the number of hemoglobin-stained blood cells when RPL5 was knocked down (**f** and **i** show the control; **g** and **j** show the RPL5 knockdown), and this was partially rescued by coinjection with P53 MO (**h** and **k**). **a**, **b**, **c**, **d**, **e**,**i**, **j** and **k** show the lateral view; **f**, **g**, and **h** show the ventral view

**Fig. 2:**
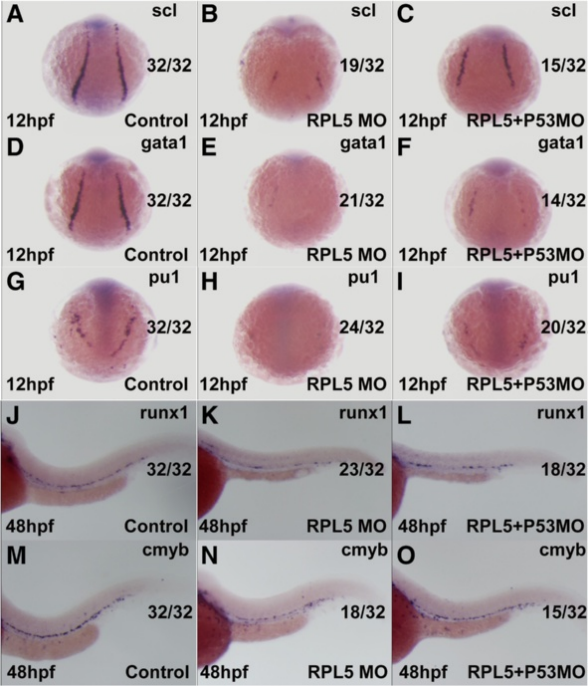
RPL5 is required for both primitive hematopoiesis and definitive hematopoiesis, which are partially mediated the by *P53* pathway in zebrafish. **a**-**c** The expression of *scl* was significantly decreased in RPL5 MO zebrafish and was partly rescued in RPL5 and *P53* double MO zebrafish at 12 hpf. **d**-**i** The expression of *gata1* and *pu1* was significantly decreased in RPL5 MO zebrafish and was partly rescued in RPL5 and *P53* double MO zebrafish at 12 hpf. **j**-**o** The expression of *c-myb* and *runx1* was significantly decreased in RPL5 MO zebrafish and was partly rescued in RPL5 and *P53* double MO zebrafish at 48 hpf

There are two isoforms of RPL5 (RPL5*a* and RPL5*b*), and the ATG MO that we designed could inhibit both isoforms *in vivo*. The effectiveness of the translation inhibition by RPL5 MO was confirmed by examining green fluorescent fusion protein expression by fluorescence microscopy (Fig. 1a and b). Embryos injected with control MO did not display any morphological changes, while RPL5 deficiency in zebrafish at a low concentration of 0.25 ng/embryo resulted in hematopoietic and developmental abnormalities resembling DBA, including a ventrally bent tail, smaller head and a reduction in the circulating blood cells as early as at 24hpf (Fig. 1c-e). At 48 hpf, hemoglobin-stained blood cells in the heart region were markedly decreased in RPL5 MO, which was partially rescued when P53 MO was co-injected with RPL5 MO, similar to our previous findings for RPS19 MO, RPL11 MO and RPS24 MO (Fig. 1f-k) [23–25]. The expression of the hemangioblast marker T-cell acute lymphocytic leukemia 1 (*scl*) was significantly decreased,as well as the primitive erythroid progenitor marker *gata1* and primitive myeloid progenitor marker Spi-1 proto-oncogene b (*pu1*) in RPL5 MO at 12 hpf. Moreover, the expression of the definitive hematopoietic stem cell markers v-myb avian myeloblastosis viral oncogene homolog (*c-myb*) and runt-related transcription factor 1(*runx1)* was also markedly decreased in RPL5 MO at 48 hpf (Fig. 2). Furthermore, co-injection of RPL5 Mo and P53 MO could partially rescue the primitive, definitive hematopoiesis defects caused by RPL5 MO alone (Fig. 2).

### Differentially expressed genes in RPL5 MO

**Fig. 3:**
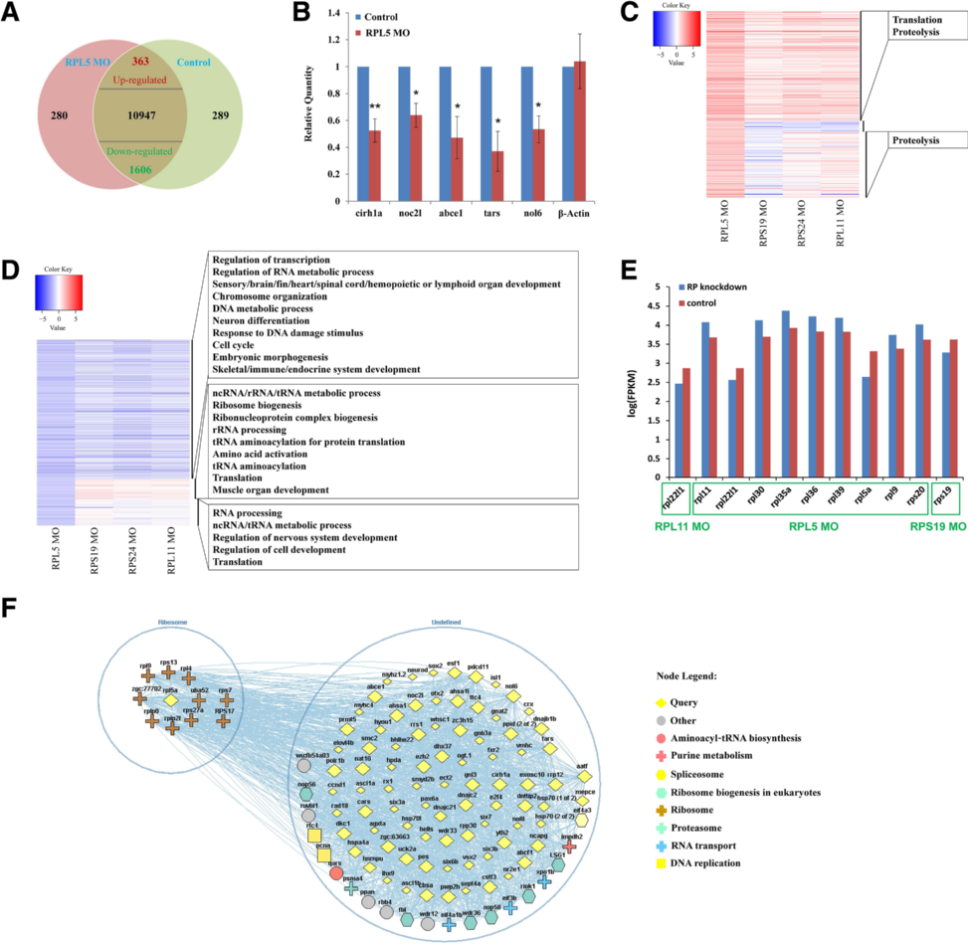
Characterization of RPL5-deficient zebrafish embryos transcriptome and identification of differentially expressed genes. **a** Transcriptome analysis identified 280 genes specifically expressed in RPL5 MO, 289 genes specifically expressed in Control, and 12916 commonly expressed genes. In the commonly expressed genes, 363 significantly up-regulated genes and 1606 significantly down-regulated genes in the RPL5 MO were detected using Cuffdiff (fold-change > 2 and *p*-value < 0.05). Venn diagram summarizes the number of differentially expressed genes, shared and distinct genes expressed in RPL5 MO and Control. **b** Representative experimental validation of the regulated genes by Real-time PCR analysis. Gene expression was represented as mean ± SD and Two-way ANOVA was performed for comparison between Control and RPL5-deficient zebrafish embryos (**P < 0.001, *P < 0.01, *n* = 3). Gene expression in Control samples was normalized to 1. **c**-**d** The expression patterns of up-regulated genes (**c**) and down-regulated genes (**d**), which was detected due to RPL5-deficiency, in other zebrafish models were showed by heatmaps. The corresponding GO enrichment analysis for genes in coordinated regulatory trend, genes in opposite regulatory trend, and genes with no regularities compared with other DBA zebrafish models was performed by DAVID tool. **e** Differentially expressed RP genes were detected in each DBA zebrafish model, which was separately compared with Control to filter the differentially expressed RP genes with threshold of fold-change > 2 and *p*-value < 0.05. **f** Network analysis of differential expressed genes based on FunCoup web source. The yellow diamond represents the significantly differential expressed gene in RPL5-deficient zebrafish embryos (FPKM > 1, fold-change > 2, FDR < 0.05). Other nodes are connected with our query genes by 1-expansion-depth. All the lines represent direct correlation of nodes

RNA-seq was used to identify and compare differential expressed genes in RPL5 morpholino (RPL5 MO) and control morpholino (Control) zebrafish embryos. Pair-end deep sequencing was performed on mRNA-seq libraries from these two samples. Using the TopHat and Cufflinks packages, more than 13,000 annotated genes were obtained, accounting for 83 % of the total genes assembled in the Zv9 zebrafish genome. We identified 363 up-regulated genes and 1606 down-regulated genes in RPL5 MO zebrafish (fold-change > 2, and *p*-value < 0.05) (Fig. 3a). The expression levels of 5 of these regulated genes (cirrhosis, autosomal recessive 1A (*cirh1a)*, NOC2-like nucleolar associated transcriptional repressor (*noc2l*), ATP-binding cassette, sub-family E (OABP), member 1(*abce1)*, threonyl-tRNA synthetase (*tars)*, nucleolar protein 6 (*nol6)*) were examined using RT-PCR, and the results confirmed that the expression pattern showed a similarly trend between the RT-PCR and RNA-seq results (Fig. 3b).

Compared with other DBA zebrafish models (such as RPS19 MO zebrafish, RPS24 MO zebrafish, and RPL11 MO zebrafish), these differentially expressed genes showed RPL5 MO had some general characters and little specificity among those models [23–25]. 214 up-regulated expressed genes in RPL5-deficient zebrafish that were related to biological functions of translation and proteolysis, showed the coordinated regulatory trend in other DBA zebrafish models (Additional file 1: Table S1). While 21 of the up-regulated genes in RPL5-deficient zebrafish were down-regulated in other DBA zebrafish models (Additional file 2: Table S2). The remaining 128 up-regulated genes, enriched in the GO term of proteolysis, showed no regularities in other DBA zebrafish models (Fig. 3c). For down-regulated expressed genes in RPL5-deficient zebrafish, 1205 out of them showed coordinated regulatory trends when compared with other models (Additional file 3: Table S3). Based on GO annotation, these genes were found enriched in GO terms of regulation of transcription, RNA/DNA metabolic process, organs and systems development, cell cycle, and so on. While 168 out of down-regulated genes in RPL5 MO, mainly related to translation process, such as ribosome biogenesis, rRNA processing, amino acid activation, and tRNA aminoacylation, had only slightly up-regulated expression in other models (Additional file 4: Table S4). There were still 233 genes with no regular expression trend in these DBA zebrafish models and were enriched in biological functions of RNA processing, regulation of cell development, and translation (Fig. 3d). It is noteworthy that a portion of significantly down-regulated genes was enriched in GO annotation of hematopoietic development, such as dyskeratosis congenita 1 (*dkc1*), CXXC finger protein 1b (*cxxc1)*, WD repeat domain 43 (*wdr43)*, heat shock protein 9 (*hspa9*), ribosomal protein L22-like 1 (*rpl22l1)*, prostaglandin-endoperoxide synthase 2a (*ptgs2a)* and odd-skipped related transcription factor 1 (*osr1)*.

### Genome-wide functional coupling network in RPL5 MO

To understand the interactive relationships among deregulated genes and identify key factors in the abnormal transcriptome of RPL5-deficient zebrafish, we input these genes into the Funcoup framework (http://funcoup.sbc.su.se/search/) to infer genome-wide functional couplings. The network was generated using 1-expansion-depth and grouped nodes for the ribosomal pathway (Fig. 3f). We found that RPL5*a*, which was significantly down-regulated in our data, was functionally coupled with many other ribosomal proteins such as RPS7, RPL9, RPS13, and RPS27, although most of them were not detected by significant expressional changes. More interestingly, these ribosomal targets were also functionally coupled with regulatory genes detected in other DBA zebrafish models. Additionally, *cirh1a*, N-acetyltransferase 10 (*nat10)*, *noc2l*, *dkc1*, *abce1*, *tars*, polymerase (RNA) I polypeptide B (*polr1b)*, activator of heat shock protein ATPase homolog 1a (*ahsa1)*, PWP2 periodic tryptophan protein homolog (*pwp2h)*, programmed cell death 11 (*pdcd11)*, nucleolar protein 6 (*nol6)*, and apoptosis antagonizing transcription factor (*aatf)* were found as central nodes in this network (connectivity > 60), which were not overlapped well with that of networks constructed by regulatory transcriptome of other RP MOs. Based on the literature, many of these genes are associated with the hematopoietic system, ribosome biogenesis and development process [26, 27].

### Deregulated molecular signaling pathways in the RPL5-deficient transcriptome

363 up-regulated genes and 1606 down-regulated genes were mapped to the KEGG pathway database (http://www.genome.jp/kegg/). Down-regulated genes in RPL5-deficient zebrafish were also enriched in several important pathways (Table 1). Spliceosome pathway was enriched by down-regulated genes mainly participating in synthesis of the small nuclear RNAs (snRNA) named U1, U2, U4, U5, and U6, which made up the major spliceosome. Down-regulated genes were also involved in the 4 phases of cell cycle (G1, S, G2, and M), especially DNA replication process in S phase. Another enriched pathway was Aminoacyl-tRNA biosynthesis pathway,which delivers the amino acid to the ribosome for incorporation into the polypeptide chain that is being produced. RPL5-deficient zebrafish showed defects when tRNA was charged to L-Glutamine, L-Alanine, L-Threonine, L-Cysteine, L-Methionine, L-Isoleucine, L-Phenylalanine, and L-Tyrosine. Besides, pathways of notch signaling, base excision repair, lysine degradation, RNA degradation, pyrimidine metabolism, and selenoamino acid metabolism were also enriched by down-regulated genes. Up-regulated genes were mapped preferentially to pathways associated with ribosome, and down-regulated genes were enriched in pathways of spliceosome, cell cycle and so on. (Table 1). We also compared the differentially expressed RP genes in different DBA zebrafish models. There were altogether 9 RP genes, expression of which was significantly changed in RPL5 MO. While rare RP genes were affected by RPS19, RPL11 or RPS24 knockdown (Fig. 3e). Apparently, RPL5 knockdown made more influences on the expression of RP genes compared with other DBA zebrafish models.

**Table 1:** Pathways enriched by regulated genes (fold-change > 2 and *p*-value < 0.05) in RPL5 MO

Expression Pattern	KEGG Pathway	Count	*P*-Value
Up-regulated	Ribosome	10	1.90E-05
Down-regulated	Spliceosome	25	2.80E-07
Down-regulated	Cell cycle	26	4.00E-07
Down-regulated	DNA replication	10	4.40E-04
Down-regulated	Aminoacyl-tRNA biosynthesis	9	1.70E-03
Down-regulated	Notch signaling pathway	11	2.20E-03
Down-regulated	Base excision repair	7	1.80E-02
Down-regulated	Lysine degradation	8	1.90E-02
Down-regulated	RNA degradation	9	3.00E-02

### Characteristic lncRNome expression patterns of RPL5 MO zebrafish and genes correlated with differentially expressed lncRNAs

**Fig. 4:**
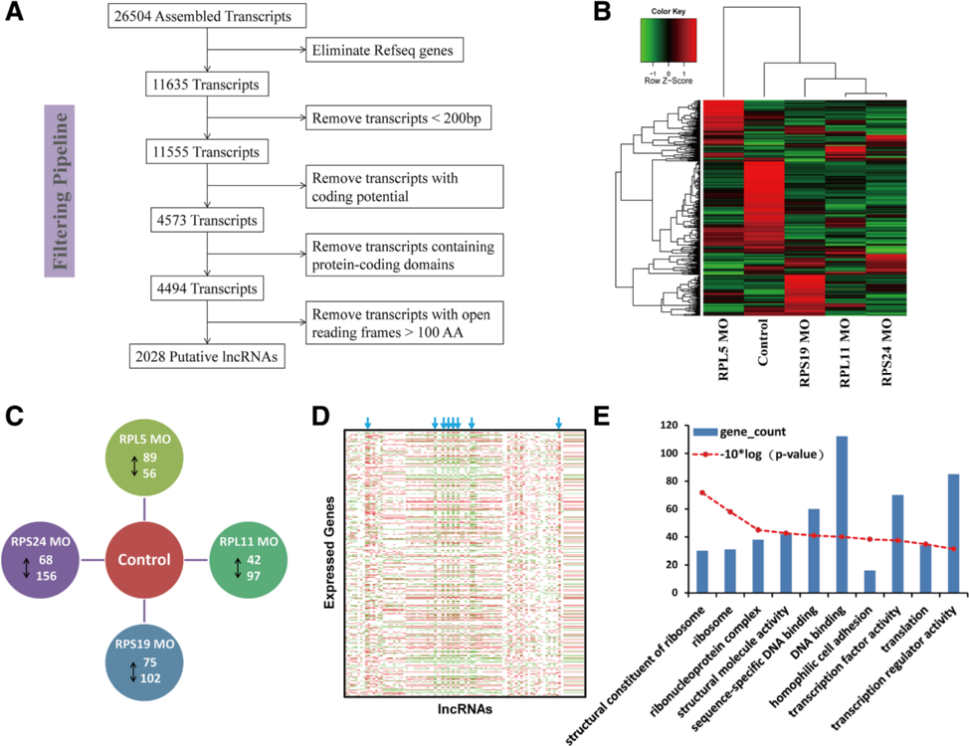
Identification of lncRNAs expressed in DBA zebrafish models. **a** Workflow for lncRNAs discovery. Details are described in “Methods”. **b** Unsupervised clustering of expression of predicted lncRNAs for diverse DBA zebrafish models and Control model. **c** Number of differentially expressed lncRNAs in different DBA zebrafish models compared with Control model, which was detected by R package DEGseq with threshold of fold-change > 2 and *p*-value < 0.05. **d** Correlation heatmap between the expression of lncRNAs and genes. Rows represent genes expressed in one of these zebrafish models, and the columns represent differentially expressed lncRNAs. The correlations between the expression of differentially expressed lncRNA and all genes are calculated by Pearson method. With the threshold of absolute correlation coefficient |R| ≥ 0.8, the significantly correlated gene-lncRNA couplings are colored by red or green. A red color indicates a positive correlation, whereas green bars represent a negative correlation. **e** Top 10 of the GO terms enriched by genes correlated with more than half of total differentially expressed lncRNAs

To determine the effects on global lncRNA transcription profile due to ribosomopathies, we analyzed the lncRNomes of DBA zebrafish models and the Control. Using coding prediction and ORF (open reading frame) identification, we found that 2028 potential lncRNAs were expressed in these zebrafish samples at 48 hpf (Fig. 4a). By unsupervised clustering of expression of all expressed lncRNAs, we found that compared with the Control, the majority of lncRNAs had lower expression in DBA zebrafish models. The expression pattern of genome-wide lncRNAs of RPL5-deficient zebrafish seemed more specific for our previous DBA zebrafish models (RPS19 MO,RPL11 MO and RPS24 MO) that are all clustered together except for the RPL5 MO in this study (Fig. 4b). Next, we identified the differentially expressed lncRNAs between DBA zebrafish models and the Control (Fig. 4c). Compared with genes, there were less commonly regulated lncRNAs between diverse DBA zebrafish models. We found only 7 commonly up-regulated lncRNAs and 8 down-regulated lncRNAs (fold-change > 2, *p*-value < 0.05). In addition, we found that 8 lncRNAs were specifically expressed in the Control sample and 24 lncRNAs were specifically in DBA zebrafish models respectively.

Since lncRNAs have been demonstrated to be involved in the transcriptional regulation, we explored the effects of the differentially expressed lncRNAs on the expression of genes in DBA zebrafish models. We analyzed the correlation between the expression of each lncRNA and expressed gene. Interestingly, we found that a set of commonly regulated lncRNAs (some were marked by arrows in Fig. 4d), was significantly associated with more than 3000 genes across the whole genome (Additional file 5: Table S5). Using an absolute correlation coefficient cutoff of greater than 0.8, we found a set of highly correlated genes (Fig. 4d). These genes were enriched in GO terms of structural constituent of ribosome, ribonucleoprotein complex and so on (Fig. 4e).

### MiRNAs involved in hematopoiesis and developmental process in RPL5-deficient zebrafish

**Fig. 5:**
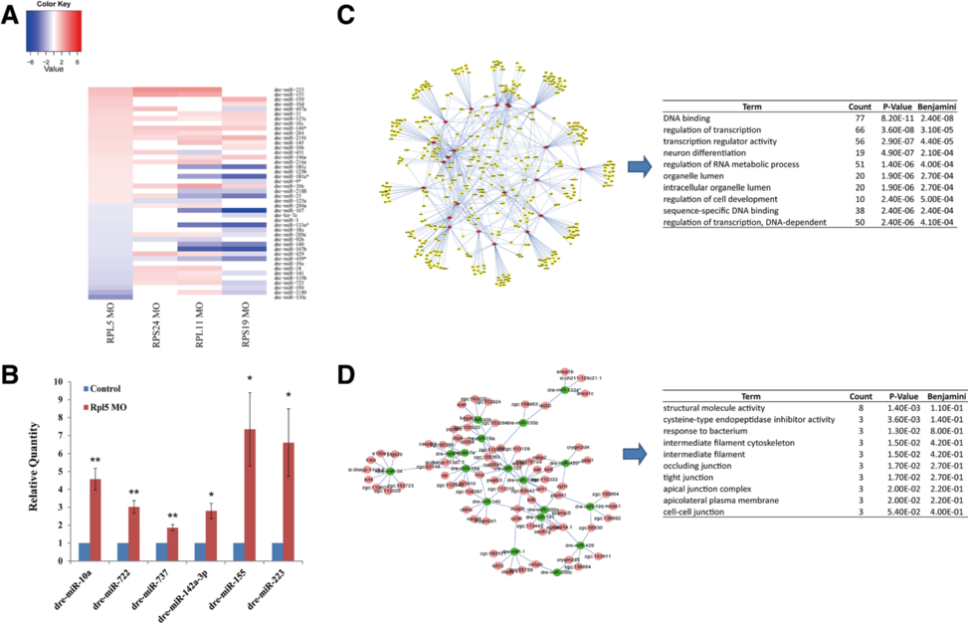
Differential expressed miRNAs and their potential targets in RPL5-deficient zebrafish embryos. **a** The heatmap shows expression pattern of miRNAs, which were significantly regulated with fold-change > 1.5 and *p*-value < 0.05 in RPL5 MO, in all DBA zebrafish models. The red module represents the up-regulated miRNA and the blue module represents the down-regulated miRNA. **b** Representative experimental validation of the regulated miRNAs by Real-time PCR analysis. miRNAs expression was represented as mean ± SD and Two-way ANOVA was performed for comparison between Control and RPL5-deficient zebrafish embryos (**P < 0.001, *P < 0.01, *n* = 3). miRNAs expression in Control samples was normalized to 1. **c**-**d** Overview of the regulatory networks containing differentially expressed miRNAs and their candidate mRNA targets in RPL5-deficient zebrafish embryos. Red/green nodes denote up/down-regulated miRNAs, and pink/yellow nodes denote their potential up/down-regulated mRNA targets. Edges denote the regulatory relationships between miRNAs and their predicted mRNA targets

To understand the observed changes in the miRNome and their biological functions in RPL5-deficient zebrafish embryos, we compared the miRNA expression profiles in RPL5 MO and the control animals. 6 of these regulated miRNAs were confirmed by RT-PCR analysis, and the ncRNA-seq results were validated using specific PCR for results with significant differences (*p*-value < 0.01) between RPL5-deficient zebrafish embryos and Control (Fig. 5b).

Using the DEGseq package, we identified 24 up-regulated miRNAs, 20 down-regulated miRNAs and 14 RPL5-deficient specific miRNAs in zebrafish (fold-change > 1.5, *p*-value < 0.05). As previously reported, some of the specifically expressed miRNAs in RPL5 MO (such as Dre-mir-142a-3p, Dre-miR-34b and Dre-miR-15a*) and some miRNAs with two-fold higher expression (such as Dre-miR-150, Dre-miR-223 and Dre-miR-155) are involved in the development and function of the hematological system and most of the above-listed miRNAs participate in normal hematologic functions by regulating the expression of *c-myb* [28–31]. Compared with other DBA zebrafish models, regulatory miRNome of RPL5-deficient zebrafish was more similar to that of RPS19 model than RPS24 model (Fig. 5a). Interesting, there was 5 miRNAs (dre-miR-125c, dre-miR-140*, dre-miR-2191, dre-miR-30b, dre-miR-459*) showing a coordinated regulatory trend among these DBA zebrafish models.

To determine the influences of regulated miRNAs on gene expression, we used the MicroCosm Targets database (http://www.ebi.ac.uk/enright-srv/microcosm/htdocs/targets/v5/) to predict the potential targets for significantly differential expressed miRNAs. We obtained 8799 target genes for up-regulated expressed miRNAs and 7760 target genes for down-regulated expressed miRNAs in RPL5-deficient zebrafish. Then, we analyzed these target genes through comparison with our RNA-seq results. Because the canonical miRNA regulation model suggests that miRNAs repress the expression of their downstream target genes [32], we overlapped down-regulated genes with the predicted targets of up-regulated miRNAs and overlapped up-regulated genes with the predicted targets of down-regulated miRNAs. As a result, we identified 73 significantly up-regulated genes and 405 significantly down-regulated genes as potential targets of regulated miRNAs in RPL5-deficient zebrafish (Fig. 5c-d). GO analysis by DAVID showed that enriched GO categories for the down-regulated targets were primarily associated with regulation of transcription, nerve system development, RNA metabolic process, and cell development.

## Discussion

### RPL5 is required for both primitive and definitive hematopoiesis

Hematopoiesis is a dynamic process consisted of primitive and definitive hematopoiesis based on the type of blood cells generated. Numerous transcription factors are involved in the complex regulation of each stage. In zebrafish, the primitive hematopoiesis starts at ∼ 11 h post fertilization (hpf) in the lateral plate mesoderm (LPM) during somitogenesis [33]. In our RPL5 MO at 12 hpf, primitive hematopoietic stem cell markers and the hemangioblast marker *scl*, which is especially critical for the development of arteries where adult hematopoietic stem cells emerge [34], was significantly decreased. The primitive erythroid progenitor marker *gata1* and primitive myeloid progenitor marker *pu1* two master regulators with a cross-inhibitory relationship that regulates the primitive erythroid and myeloid fates, were also markedly decreased. These data suggest that primitive hematopoiesis is affected by RPL5 downregulation.

The onset of definitive hematopoiesis is marked by the specification of HSCs, which support hematopoiesis throughout the life of a vertebrate. *C-myb* and *runx1* have been used as the earliest markers of definitive hematopoiesis due to their expression in the aorta-gonad-mesonephros (AGM) during HSCs specification. In our RPL5 MO at 48 hpf, the definitive hematopoietic stem cell markers *c-myb* and *runx1* displayed similarly decreased expression pattern. Hemoglobin-stained blood cells in the heart region at 48 hpf were also markedly decreased, similar to the *Rps19* MO, *Rpl11* MO and *RPs24* MO phenotypes that we previously observed (Fig. 1) [23–25].

Some of the significantly down-regulated genes in RPL5 MO also play important roles in primitive and definitive hematopoiesis. For instance, *osr1* is required for the development of hemangioblast [35]. Specific depletion of *cxxc1*, which was identified as a commonly down-regulated genes among the four DBA zebrafish models, inhibited genomic cytosine methylation and primitive hematopoiesis in zebrafish [36]. It has been demonstrated that deficiency of *dkc1* in zebrafish led to reduced definitive hematopoiesis mediated by *P53* [37]. Moreover, *wdr43* was identified as certain subunits required for definitive hematopoiesis [38]. Loss of *hspa9* in zebrafish recapitulated the ineffective hematopoiesis of the myelodysplastic syndromes [39].

In addition, many screened miRNAs were hematopoietic-specific, especially dre-miR-142a-3p, which can result in the loss of hematopoiesis during embryonic development in zebrafish [28]. Moreover, dre-miR-34b, dre-miR-15a* and dre-miR-150, which were thought to target *c-myb*, were significantly up-regulated.

Based on the RPL5 MO phenotypes, expression patterns of key genes and our mRNA-seq and ncRNA-seq results, we believe that RPL5 is closely related to both primitive hematopoiesis and definitive hematopoiesis.

### Ribosome biogenesis were affected more in RPL5 MO than in other RP MOs

Haploinsufficiency of RPs in DBA leads to not only pure red cell aplasia but also developmental defects [3]. Notably, mutation in RPL5 had a higher rate of malformation and was associated with a more severe phenotype than mutations in RPS19 [9]. In zebrafish, knockdown of ribosomal proteins leads to hypoplasia of the yolk sac extension and gross morphological defects in the head region [40]. Our RPL5 MO (0.25 ng/embryo) results showed a more severe defective phenotype, when compared with our previous RPL19 MO (2 ng/embryo), RPL11 MO (0.5 ng/embryo), RPS24 MO (0.5 ng/embryo) results, such as the severe curly tail morphology at 24 hpf and almost invisible expression of hemangioblast marker gene *scl* primitive erythroid progenitor marker *gata1* and myeloid progenitor marker *pu1* at 12 hpf (not shown).

Working as a nucleocytoplasmic shuttle protein RPL5 plays a central role during the process of ribosomal assembly. In this study, using mRNA-seq analysis, we found about 2/3 of total differentially expressed genes in RPL5 MO zebrafish had a concordant variation tendency in expression level with other DBA zebrafish models. These genes were related to functions of translation, regulation of transcription and so on. While the remaining genes (~1/3 of total differential expressed genes in RPL5 MO), which were showing specific expressional changes compared with other DBA zebrafish models, played important roles in proteolysis, ribosome biogenesis and ribonucleoprotein complex biogenesis. Interestingly, the up-regulation of ribosome pathway that was detected by pathway enrichment analysis in RPL5 MO, was not found in other DBA zebrafish models. This apparent acceleration of ribosomal biogenesis processes in RPL5 MO zebrafish may be caused by the feedback adjustments of RPL5 downregulation, but less severe in other DBA zebrafish models. Furthermore, comparing the differentially expressed ribosomal proteins in various DBA zebrafish models, it seems that RPL5 knockdown affects more the expression of ribosomal proteins. Our result confirmed the vital position of RPL5 in ribosome biogenesis and related biological process. It also provided a transcriptomic research basis to explain the special and more severe clinical manifestation of RPL5 mutated DBA patients in comparison with other DBA patients.

Interestingly, the genes correlated with more than half of the differentially expressed lncRNAs were also enriched in GO terms of structural constituent of ribosome and ribonucleoprotein complex. It implicates that RPL5 may have an important role in ribosome biogenesis by regulating some lncRNAs.

### Both P53 dependent and independent pathways such as Aminoacyl-tRNA biosynthesis pathway play important roles in DBA pathology

The role of *P53* pathway played in the pathogenesis of DBA has been clearly demonstrated while the specific regulatory mechanism is still largely unknown. In our current study, the co-injection of *P53* MO could partially rescue the morphological and hematopoiesis defects caused by RPL5 MO. From the transcriptome analysis, although the P53 signaling pathway was not significantly enriched, some genes involved in this pathway were detected significantly regulated, such as *p53*, *mdm4*, cyclin D2 a(*ccnd2a)*, growth arrest and DNA-damage-inducible, alpha a(*gadd45aa)*, growth arrest and DNA-damage-inducible, beta b (*gadd45bb)*, topoisomerase (DNA) II binding protein 1 (*topbp1)*, guanine nucleotide binding protein-like 3 (*gnl3)*, (glycogen synthase kinase 3 beta) *gsk3b*, cyclin D1 (*ccnd1)*, and checkpoint kinase 1 (*chek1)*. Moreover, the direct miRNA targets of P53, that were dre-miR-34a, dre-miR-145 and dre-miR-15a [41], were differentially expressed in RPL5 MO zebrafish when compared with the Control. P53 also is known to play an important role in some down-regulated genes enriched pathways that were mapped in RPL5-deficient zebrafish. For example, P53 participates in the cell cycle checkpoint control, such as G1/S checkpoint regulation and G2/M DNA damage checkpoint regulation. We found many cell cycle regulation genes were significantly down-regulated in all four phases of the cell cycle.

Based on the transcriptome analysis, we also found some important signaling pathways that were not correlated with P53 directly or indirectly, such as aminoacyl-tRNA biosynthesis pathway. Aminoacyl-tRNA biosynthesis is an important part of the translation process. In our data set, we found that tRNA charged to L-Glutamine, L-Alanine, L-Threonine, L-Cysteine, L-Methionine, L-Isoleucine, L-Phenylalanine, and L-Tyrosine was inhibited by down-regulated genes. These might have some correlation with amino acid L-leucine. It was reported that L-leucine, which increases translation via the mTOR pathway and the phosphorylation of S6K and 4E-BP, resulted in an improvement of anemia in zebrafish models of both DBA and del(5q) syndrome [42]. Another study showed that the administration of L-leucine significantly improved anemia in RPS19-deficient mice, increased the bone marrow cellularity, and alleviated stress hematopoiesis [43]. Thus, we believe that dysfunction in this pathway may lead to DBA symptoms via a reduction in leucine and the translation process.

## Conclusion

Mutations in RPL5 are reported in approximately 9 ~ 21 % of DBA patients, which represents the most common condition related to a large-subunit ribosomal protein. Mutation in RPL5 is associated with a more severe clinical phenotype [9].

In this study, we successfully generated zebrafish RPL5 morphants. Transcriptome deep sequencing was used to characterize the transcriptome profile of RPL5 MO zebrafish by comparing with other DBA zebrafish models and found the common regulated genes, lncRNAs and miRNAs for various kinds of DBA models. Our results show that RPL5 downregulation in zebrafish results in hematopoietic and developmental abnormalities that resemble human DBA. Both primitive hematopoiesis and definitive hematopoiesis were disturbed in RPL5 MO. Ribosome biogenesis and translation process were more affected in RPL5 MO than in other RP MOs, which may explain the clinical specialty of RPL5 mutated DBA patient. Moreover, both P53 dependent and independent pathways such as Aminoacyl-tRNA biosynthesis pathway play important roles in DBA pathology. Since almost all of genes that we screened were conserved in humans, future functional studies in mammalian and human cells are needed to ascertain the role of these pathways and signaling molecules.

## Methods

### Ethics statement

All of the studies using zebrafish were approved by the Animal Care and Use Committee of Huazhong University of Science and Technology.

### Zebrafish embryo maintenance, RPL5 morpholino (MO) microinjection, and hemoglobin staining

Wild-type zebrafish (*Danio rerio*; AB type) were bred and maintained under standard library conditions. Zebrafish embryos were kept in a 28.5 °C incubator, and the embryonic stages evaluated in this study have been previously described [44, 45]. The RPL5 MO (5-ACCCATTTTGTGATCGTTTGTTCTC-3), control MO (5- ACCCGTTTCGTAATCGTCTGTGCTC-3) and *P53* MO (5- GCGCCATTGCTTTGCAAGAATTG-3) were obtained from Gene-Tools, LLC (Philomath, OR, USA). Zebrafish embryos at the one-cell stage were injected with the MOs using a microinjector (WPI SYS-PV830). Based on our initial trials, 0.25 ng RPL5MO and control MO was chosen as the optimal concentration. Injected embryos were grown at 28.5 °C and observed under a microscope. The effectiveness of translational inhibition by RPL5 MO was tested *in vivo* using the RPL5:egfp green fluorescent fusion protein under fluorescence microscopy. The O-dianisidine (Sigma) staining protocols were performed as previously described [23] [24] and images were collected using an Olympus microscope with a digital camera (OLYMPUS IX71) and imported into Adobe Photoshop CS2 9.0.2 for orientation and figure preparation.

### Total RNA isolation, library preparation, and sequencing

Immediately after harvesting, 40–50 pooled embryos at 48 hpf from different experimental replicates were snap-frozen in liquid nitrogen and stored at −80 °C. Total RNA was extracted from pooled embryos using Trizol reagent (Invitrogen) according to the manufacturer’s instructions. RNA concentrations were determined using a NanoDrop 2000 (Thermo Scientific). The integrity of the RNA samples was checked using 1.2 % agarose gel electrophoresis, followed by removal of residual genomic DNA with RNase-free DNase I (Ambion).

Libraries of mRNA and miRNA were constructed using the Illumina mRNA-Seq and ncRNA library preparation kit according to the manufacturer’s instructions, respectively. The concentration and size distribution of the libraries were checked using an Agilent Bioanalyzer DNA 2000 chip (Agilent Technologies), followed by sequencing on an Illumina Hiseq 2000 sequencing platform. The RNA-Seq library was sequenced with 2 × 100 bp in pair-end mode by 100-bp lengths, and the ncRNA library was sequenced in single-end mode by 80-bp lengths. A total of 37–40 million reads were collected for RNA-Seq analysis and 3.8–13.5 million reads for ncRNA analysis.

### Mapping, annotation, and expression difference analysis for mRNA-seq data

Reads were processed and aligned to the UCSC zebrafish reference genome (build Zv9/danRer7, Jul. 2010) using TopHat (version 1.3.3) [46]. TopHat incorporates the Bowtie v0.12.7 algorithm to perform alignments. In brief, TopHat initially removes a portion of reads based on the quality of information accompanying each read and maps qualified reads to the reference genome. The reference index was built using Bowtie with a fasta file for the entire genome of zebrafish, which was downloaded from UCSC (http://genome.ucsc.edu/). Parameters were set by default, but the number of threads to align reads was set at 6. TopHat aligned read files were then entered into the Cufflinks (version 1.2.1) software for further analyses, including transcript assembly, abundance estimation, and differential expression and regulation testing in RNA-Seq samples [47]. To calculate the gene expression intensity, read counts were normalized to the number of fragments per kilobase of transcript per million mapped reads (FPKM) according to the gene length and total mapped reads [48]. Confidence intervals for estimates of FPKM were calculated using a Bayesian inference method [49]. Cuffdiff then performed the differential expression tests at the level of transcripts, primary transcripts and genes [49]. Differential expressed genes were characterized according to the criterion of a fold-change > 2 and *p*-value < 0.05.

### lncRNome identification and characterization

Cufflinks script was used to determine whether the detected transcripts were annotated by Refseq genes of zebrafish genome (build Zv9/danRer7, Jul. 2010). All of the annotated transcripts were eliminated, and the non-annotated transcripts were used for downstream analysis. Next, transcripts with a length more than 200 bp were filtered out for coding potential prediction analysis using Coding Potential Calculator software (CPC, http://cpc.cbi.pku.edu.cn/), which assessed the protein-coding potential of a transcript based on six biologically meaningful sequence features [50]. Transcript whose predicting result from CPC was ‘noncoding’ was considered as noncoding RNA of high potential and kept for the next step analysis [19]. Further, the selected transcripts translated in all 6 reading frames into amino acid sequences were matched to Pfam database by PfamScan, which was used to identify transcripts containing protein-coding domains [51]. For PfamScan, a cutoff e-value of 0.01 was used. Then the remaining transcripts were analyzed by GETORF, which was widely used to find and extract open reading frames (ORFs) (http://emboss.sourceforge.net/apps/cvs/emboss/apps/getorf.html) [52]. Transcripts containing ORFs of more than 300 nucleotides were removed, and the remaining transcripts were classified as putative lncRNAs.

### Mapping, annotation, and expression difference analysis for miRNA-seq data

First, FASTX-Toolkit clipper was used to remove sequencing adapters. Then, we converted the fastq file to a tab-delimited file, which held only the unique sequence read (tag) and the corresponding number of copies. After data preprocessing, we uploaded this file to DSAP (http://dsap.cgu.edu.tw) for the clustering of tags and classification of non-coding small RNAs and miRNAs based on sequencing homology searches against the Rfam and miRBase databases, respectively [53]. The differentially expressed miRNAs were detected with the R package DEGseq using the output data from DSAP. The threshold for filtering out the differentially expressed miRNAs is fold-change > 1.5 and *p*-value < 0.05.

### Gene ontology analysis and network construction

DAVID tool (https://david.ncifcrf.gov) was used for identifying enriched biological themes and discovering enriched canonical pathways in KEGG database [54, 55]. Enriched GO terms with Gene-Count > 5 and *p*-value < 0.05 were accepted for further discussion. We then constructed the functional coupling network for differentially expressed genes using FunCoup v3.0 (http://funcoup.sbc.su.se/search/), which provided an unspecific form of association encompassing direct physical interactions but also more general types of direct or indirect interactions such as regulatory interactions or participation the same process or pathway [56]. In addition, the linkages between genes and miRNAs were constructed based on the targeting information from the MicroCosm Target database (http://www.ebi.ac.uk/enright-srv/microcosm/htdocs/targets/v5/). Visualization of the regulatory relationship is implemented by Cytoscape software [57]. The importance of nodes in the networks was measured based on their connectivity, and the core molecules of the networks were considered nodes that were connected with greater numbers of edges.

### Quantitative Real-time PCR and Whole mount in situ hybridization

Real-time PCR of mRNA and miRNA was performed using SYBR Green PCR Master Mix (Fermentas, Guangzhou, China) and the All-in-OneTM miRNA qPCR Kit (GeneCopoeia, Maryland, USA), respectively, according to the manufacturer’s instructions. The experiments were repeated at least in triplicate. The primers for real-time PCR are shown in Additional file 6: Table S6. The digoxigenin-labeled antisense riboprobes were transcribed from a linearized plasmid containing g*ata1*, *pu1*, *scl*, *c-myb* and *runx1* using DIG RNA labeling Mix and T7 RNA polymerase (Roche, USA) [37, 58]. Whole-mount in situ hybridization was conducted as previously described [23].

### Data access

Supporting data sets are available in the Gene Expression Omnibus, with the accession number GSE58347, GSE54270, GSE51326, and GSE45699.

## Additional files

Additional file 1: Table S1. Up-regulated genes in RPL5 MO (fold-change > 2 and *p*-value < 0.05) showed coordinated regulatory trend in other DBA zebrafish models. (DOC 196 kb) Additional file 2: Table S2. Up-regulated genes in RPL5 MO (fold-change > 2 and *p*-value < 0.05) showed opposite regulatory trend in other DBA zebrafish models. (DOC 39 kb) Additional file 3: Table S3. Down-regulated genes in RPL5 MO (fold-change < 0.5 and *p*-value < 0.05) showed coordinated regulatory trend in other DBA zebrafish models. (DOC 976 kb) Additional file 4: Table S4. Down-regulated genes in RPL5 MO (fold-change < 0.5 and *p*-value < 0.05) showed opposite regulatory trend in other DBA zebrafish models. (DOC 159 kb) Additional file 5: Table S5. LncRNAs commonly regulated (fold-change > 2 and *p*-value < 0.05) in DBA zebrafish models and expression patterns correlated with more than 3000 genes (DOC 32 kb) Additional file 6: Table S6. The primers designed for real-time PCR (DOC 33 kb)

## Abbreviations

AGM: aorta-gonad-mesonephros
DBA: Diamond-Blackfan anemia
del(5q) MDS: myelodysplastic syndrome with loss of chromosome 5q
hpf: hours post fertilization
HSC: hematopoietic stem cell
IBMFS: inherited bone marrow failure syndromes
iPSC: induced pluripotent stem cell
lncRNA: long noncoding RNA
LPM: lateral plate mesoderm
miRNA: microRNA
MO: morpholino
ORF: open reading frame
RP: ribosomal protein
