# Supplementary material for: Transcriptome analysis reveals a ribosome constituents disorder involved in the RPL5 downregulated zebrafish model of Diamond-Blackfan anemia
Source: BMC Med Genomics. 2016 Mar 9;9:13. doi: 10.1186/s12920-016-0174-9 (PMC4785739; doi:10.1186/s12920-016-0174-9)
Supplement: Additional file 1: Table S1. — Up-regulated genes in RPL5 MO (fold-change > 2 and p-value < 0.05) showed coordinated regulatory trend in other DBA zebrafish models. (DOC 196 kb) [file 12920_2016_174_MOESM1_ESM.doc]

**Supplemental Tables**

**Table S1 Up-regulated genes in RPL5 MO (fold-change > 2 and p-value < 0.05) showed coordinated regulatory trend in other DBA zebrafish models.**

| gene_symbol | fc_RPL5 | fc_RPS19 | fc_RPS24 | fc_RPL11 |
| --- | --- | --- | --- | --- |
| LOC100002334 | 3.6179498 | 2.0169551 | 2.8141957 | 1.7392091 |
| LOC100002960 | 6.2240622 | 2.2547271 | 7.3209272 | 4.2444944 |
| LOC100329294 | 4.990063 | 3.6260363 | 9.1823302 | 1.6363264 |
| LOC794625 | 2.1147284 | 1.6224725 | 1.126678 | 1.4703157 |
| LOC799177 | 2.9469293 | 1.9464656 | 2.0165217 | 1.262294 |
| abracl | 2.1326759 | 1.4880669 | 1.3585166 | 1.2518818 |
| adam28 | 10.878906 | 4.1820162 | 4.5772236 | 3.1808819 |
| anapc11 | 2.7018958 | 1.8280349 | 1.3506255 | 1.7842796 |
| ankrd22 | 4.336911 | 1.5345772 | 1.3463343 | 2.4099721 |
| anxa1a | 2.2113834 | 1.7673415 | 2.6218409 | 1.4436891 |
| anxa1b | 5.0302991 | 1.3500588 | 2.7981413 | 2.10571 |
| anxa2a | 3.0014757 | 2.5785937 | 2.1375343 | 1.9551173 |
| anxa3a | 2.6189713 | 1.64721 | 2.199714 | 1.318895 |
| ap1s3b | 2.3871172 | 1.2925877 | 1.7439977 | 2.0102896 |
| arf4 | 34.974568 | 6.8854722 | 19.952183 | 35.413991 |
| arl11 | 4.9955397 | 1.5776054 | 3.5881794 | 1.7643857 |
| arrdc3b | 2.3348192 | 1.5570081 | 2.7994748 | 2.4769342 |
| atf3 | 2.0019049 | 1.4345849 | 1.7354549 | 3.2222605 |
| b2m | 7.5714096 | 3.4446279 | 3.7374322 | 2.7029392 |
| b4galt1 | 3.8423981 | 1.3544054 | 2.8358842 | 2.1237502 |
| cabp1a | 10.441773 | 5.8379808 | 6.3929951 | 7.4592323 |
| capg | 2.1839182 | 1.240708 | 1.5362478 | 1.1741383 |
| casp6l1 | 7.2750135 | 1.2909502 | 2.2864687 | 1.3280004 |
| cd9b | 2.6119339 | 1.1047225 | 1.7850612 | 1.7051007 |
| cebpd | 3.6845587 | 1.7800416 | 2.8154529 | 3.1764144 |
| cfl1 | 3.3350423 | 1.9540965 | 2.3146451 | 1.9591024 |
| ckba | 2.097847 | 1.5092468 | 1.8489589 | 1.361405 |
| cldn1 | 2.9669689 | 4.1635883 | 3.2110869 | 4.030683 |
| cldnf | 6.1665803 | 1.6685127 | 2.2895042 | 3.0343056 |
| cldni | 2.3870428 | 1.2313141 | 1.2954377 | 1.1709604 |
| clica | 2.3520802 | 1.0166269 | 1.0390098 | 1.0073218 |
| cmah | 4.0575279 | 1.0257891 | 1.8220004 | 1.5558842 |
| cox7a2 | 2.5039119 | 1.8485417 | 1.5215995 | 1.6386258 |
| cox7b | 2.2071344 | 1.5650926 | 1.6034285 | 1.5083113 |
| crebl2 | 2.2924829 | 1.2981756 | 1.3096209 | 1.3898875 |
| cx35.4 | 2.1654957 | 1.0549243 | 1.3301728 | 1.0922424 |
| cyp17a1 | 5.7643184 | 1.3965388 | 2.3754126 | 2.1619455 |
| cyp1a | 2.5405733 | 1.4208496 | 1.8671683 | 1.1032521 |
| cyp24a1 | 2.9689633 | 7.7741539 | 4.6963996 | 6.8508868 |
| cyp2aa3 | 145.29454 | 13.402459 | 64.11393 | 41.378969 |
| cyt1 | 6.0080159 | 1.4788472 | 2.1742341 | 1.8739755 |
| cyt1l | 6.6427363 | 1.5300674 | 2.6672973 | 2.2518794 |
| dbnla | 3.1037505 | 1.3080499 | 1.3351613 | 1.6081298 |
| ddx43 | 2.8201937 | 3.2142424 | 1.6103195 | 2.2291318 |
| dhrs9 | 3.5083356 | 1.8077273 | 1.4861092 | 1.2750505 |
| dicp3.1 | 8.2656729 | 5.1344055 | 5.8027459 | 4.5863847 |
| dkk1b | 2.0703771 | 1.4176916 | 1.8642856 | 1.0102514 |
| dnajc19 | 2.3897366 | 1.8679099 | 1.9182919 | 1.4426575 |
| dnajc5gb | 3.3620251 | 1.5164329 | 1.4768331 | 1.9629771 |
| dusp5 | 5.0279154 | 1.7894135 | 2.0700052 | 3.1031162 |
| egr2a | 4.6819347 | 1.0948582 | 1.3472112 | 2.9270117 |
| eps8l1 | 3.7382675 | 1.0281756 | 1.8907774 | 2.2340272 |
| esyt3 | 2.3460541 | 1.1449663 | 1.1823548 | 1.3486801 |
| ets2 | 5.3643134 | 2.7795453 | 3.7116365 | 3.8559754 |
| f2rl1.1 | 5.1766838 | 1.7679668 | 4.349335 | 6.0938512 |
| fam20a | 7.962626 | 3.0178462 | 3.2975276 | 2.8592807 |
| fbp1a | 2.0801536 | 1.1730852 | 1.3207472 | 1.5554326 |
| fos | 5.3798153 | 1.5936565 | 4.9456086 | 6.3476597 |
| fosl1 | 2.6890787 | 1.2260122 | 1.9484565 | 3.7453402 |
| foxq1a | 7.0464081 | 1.0265263 | 4.0693626 | 3.5689883 |
| fut9d | 2.753143 | 1.500612 | 3.8958295 | 1.9774781 |
| gadd45aa | 2.2768152 | 2.3815188 | 2.3104255 | 1.7698488 |
| gadd45bb | 2.2556014 | 1.4100823 | 2.3390905 | 2.7309996 |
| gbgt1l4 | 2.5622625 | 1.4813168 | 1.2952018 | 1.3437424 |
| ggcta | 3.1757899 | 2.1238985 | 1.3092981 | 1.288446 |
| glrx | 2.2049764 | 1.0726035 | 1.3801924 | 1.2450395 |
| gna14 | 3.3270703 | 1.564976 | 1.519504 | 1.8072469 |
| gnrh3 | 4.6013053 | 2.9074133 | 3.015452 | 4.3085548 |
| gpr183 | 3.9720171 | 1.5013746 | 3.9668314 | 2.4741584 |
| hoxc12b | 3.8848222 | 2.8777138 | 2.6080614 | 2.1337596 |
| hsd17b12a | 3.4333224 | 1.1894128 | 1.694379 | 2.2336717 |
| htra3a | 15.654419 | 12.328846 | 16.546978 | 13.364748 |
| icn | 4.2089744 | 1.4684781 | 1.9607602 | 2.3007009 |
| icn2 | 3.2869437 | 1.2910537 | 2.4057203 | 2.4812115 |
| jdp2 | 2.2231288 | 1.4028452 | 1.7453537 | 2.0902866 |
| junba | 4.6599298 | 2.0898453 | 4.3627883 | 4.7378987 |
| junbb | 3.7158171 | 2.3733614 | 4.3278967 | 4.7285551 |
| kazald2 | 5.0550929 | 2.3675995 | 1.9152218 | 1.6659386 |
| krt4 | 3.6263617 | 1.4451579 | 2.2729794 | 2.211965 |
| lamb4 | 3.0553086 | 2.4036001 | 4.6415168 | 2.6072072 |
| lect2l | 7.7699522 | 1.1823208 | 1.1883922 | 1.8151256 |
| lmo7a | 2.1008457 | 1.1125814 | 1.0180372 | 1.3868536 |
| lrata | 5.0672969 | 3.0396012 | 2.4387861 | 2.4791782 |
| lsm6 | 2.4496175 | 1.7645178 | 1.3664704 | 1.5698329 |
| lxn | 2.0156932 | 2.4702266 | 2.2488163 | 1.8441415 |
| mibp2 | 2.1855185 | 1.6260546 | 1.5190356 | 2.1045598 |
| mid1ip1a | 3.7736972 | 1.1115283 | 3.1907417 | 2.0200451 |
| mmp13a | 3.5837674 | 1.2654842 | 1.3797551 | 2.1643609 |
| mmp30 | 3.1363756 | 3.2321697 | 2.6749384 | 2.3707149 |
| mmp9 | 4.1463591 | 2.2927266 | 1.7500421 | 2.4029965 |
| mpzl3 | 2.7893794 | 1.3306277 | 1.9498417 | 1.5742839 |
| ms4a17a.4 | 3.4910092 | 5.2184477 | 1.8318177 | 2.250727 |
| mvp | 2.3094552 | 1.8664647 | 1.6022493 | 1.7124951 |
| nadka | 4.6181116 | 1.1266682 | 1.3238238 | 2.0082883 |
| ndufa5 | 2.2621221 | 1.3531339 | 1.2971241 | 1.2062451 |
| ndufs6 | 2.1398869 | 1.6828133 | 1.4899299 | 1.5139268 |
| nfkbiaa | 5.4339364 | 1.1425797 | 2.414959 | 2.341388 |
| nr4a1 | 2.4770929 | 1.4594612 | 1.5905086 | 2.1621452 |
| osbpl7 | 2.9867383 | 1.2680618 | 1.2916211 | 1.7603666 |
| paqr8 | 7.2530595 | 1.286856 | 1.8308534 | 1.5905021 |
| phf5a | 2.3440192 | 1.277021 | 1.2532272 | 1.4156771 |
| phlda2 | 2.0345506 | 1.1193517 | 2.1710481 | 1.4095985 |
| pin4 | 2.1717659 | 1.6131088 | 1.6853353 | 1.5975023 |
| pparaa | 2.8320486 | 2.0092622 | 1.998071 | 1.8482995 |
| prrg4 | 2.7491061 | 1.3144523 | 1.8317451 | 1.7360009 |
| psma6b | 4.754606 | 1.2345201 | 1.1437086 | 2.6525592 |
| psme1 | 3.9176565 | 1.4067579 | 1.3982307 | 1.626194 |
| ptgis | 3.280307 | 1.5545388 | 2.1361791 | 2.4295444 |
| ptgs2a | 2.4000256 | 1.6128261 | 2.1907335 | 2.4549044 |
| ptpmt1 | 2.2331299 | 1.7550182 | 1.6683113 | 1.6996743 |
| pvalb3 | 2.6471798 | 1.1948188 | 1.2995578 | 1.2730606 |
| pycard | 2.7890481 | 1.9488662 | 1.719986 | 1.7805457 |
| rpl11 | 2.5066915 | 1.7042731 | 1.6328498 | 1.0541418 |
| rpl30 | 2.7154931 | 1.6034067 | 1.6536387 | 1.7016768 |
| rpl35a | 2.8340155 | 1.4028433 | 1.4867092 | 1.7684412 |
| rpl36 | 2.4985369 | 1.4978369 | 1.2966867 | 1.6825429 |
| rpl9 | 2.3114408 | 1.3775549 | 1.2781821 | 1.507821 |
| rps20 | 2.4997425 | 1.1500885 | 1.3445992 | 1.1863392 |
| s100a1 | 11.633313 | 8.5462795 | 3.639198 | 12.892018 |
| s100a10b | 2.6238802 | 1.9348143 | 1.661487 | 1.3990778 |
| sc:d0139 | 4.0053034 | 1.0157215 | 1.6989549 | 2.7304014 |
| sec61g | 3.1567308 | 1.2329095 | 1.2929372 | 1.520018 |
| sepw2b | 4.318193 | 1.0232275 | 1.7595044 | 1.614598 |
| sh3gl1a | 2.7792208 | 1.9175971 | 2.5717656 | 2.0778222 |
| si:ch211-117m20.5 | 4.106238 | 2.2427149 | 2.6713088 | 3.1226804 |
| si:ch211-121a2.2 | 3.3139197 | 5.6447252 | 2.7060389 | 2.7794852 |
| si:ch211-129c21.1 | 2.3714808 | 1.2025973 | 1.4472538 | 1.3914625 |
| si:ch211-160d20.1 | 2.4367137 | 1.3567234 | 1.1630922 | 1.1742338 |
| si:ch211-229n2.7 | 4.7504971 | 1.6494566 | 3.5884019 | 2.2784935 |
| si:ch211-244h7.5 | 7.9631023 | 1.2770887 | 2.306409 | 3.9119782 |
| si:ch73-13b6.3 | 3.9580894 | 1.6668038 | 2.1993781 | 2.2217141 |
| si:dkey-24f17.5 | 10.389551 | 14.840939 | 21.03758 | 10.62181 |
| si:dkeyp-113d7.4 | 3.3379993 | 1.4788577 | 2.0719666 | 2.2607811 |
| si:dkeyp-113d7.7 | 9.586046 | 2.8590328 | 3.0075636 | 2.8375953 |
| si:dkeyp-11g8.6 | 7.4275352 | 2.1775703 | 2.6583241 | 4.4408174 |
| slc12a8 | 4.9572171 | 1.5389175 | 3.6654034 | 3.8091631 |
| slc6a11 | 2.3386896 | 1.7012838 | 1.5044741 | 1.5496296 |
| slc9a2 | 3.7791481 | 1.42191 | 2.3432373 | 2.9353509 |
| smx5 | 2.3574257 | 1.7929183 | 1.4442938 | 1.5960445 |
| socs3a | 3.1067053 | 1.7932111 | 3.2360121 | 4.105072 |
| stard10 | 5.3425289 | 1.068742 | 2.0023678 | 2.0509442 |
| tctex1d1 | 3.4494257 | 1.9104628 | 2.2795207 | 1.5275705 |
| tlcd1 | 7.2927385 | 3.6474443 | 6.2312442 | 4.4497166 |
| tmem14c | 2.1034786 | 1.4073693 | 1.4736752 | 1.4294883 |
| tmem176l.4 | 2.202333 | 1.941057 | 1.8369564 | 1.1415563 |
| tmem238 | 2.6037891 | 1.7636567 | 1.1207305 | 1.5071413 |
| tmprss13a | 5.3962641 | 1.0693135 | 1.4251974 | 2.7637982 |
| tnfb | 4.1538854 | 1.658425 | 2.0909775 | 1.6686431 |
| tnni1al | 2.4981698 | 1.9682677 | 2.4980409 | 1.5847482 |
| tomm5 | 2.003298 | 1.6271614 | 1.1888481 | 1.2962171 |
| traf4b | 2.2710601 | 1.6367783 | 2.0295097 | 1.8896133 |
| vegfaa | 3.4911477 | 1.7705785 | 2.4471215 | 2.3146923 |
| wu:fb15g10 | 3.2640805 | 1.5201042 | 1.6423606 | 1.6699513 |
| wu:fe18c06 | 5.9283422 | 3.6804674 | 4.5840998 | 1.3211884 |
| zgc:100868 | 3.2511829 | 2.0752079 | 1.6477271 | 2.7033992 |
| zgc:101000 | 2.4910553 | 1.4825352 | 2.0197999 | 1.5283129 |
| zgc:101565 | 13.263054 | 1.5162414 | 4.7556139 | 5.6683856 |
| zgc:101810 | 3.8489494 | 2.3557391 | 3.2153133 | 2.0009221 |
| zgc:109888 | 3.1393925 | 1.601726 | 1.4339401 | 1.6979865 |
| zgc:110283 | 3.5649805 | 1.6735972 | 1.7796611 | 2.1525499 |
| zgc:110307 | 2.1992647 | 1.7891562 | 2.1436142 | 1.8049131 |
| zgc:110333 | 2.5754039 | 1.1848395 | 1.7255605 | 1.4758017 |
| zgc:110340 | 2.0689276 | 1.1169965 | 4.0751248 | 3.2036674 |
| zgc:110788 | 7.9830485 | 1.9466506 | 4.7616822 | 2.9690874 |
| zgc:111983 | 6.6320848 | 1.9054869 | 2.8790252 | 1.457949 |
| zgc:112009 | 3.6838136 | 3.4321879 | 3.9377087 | 1.2272043 |
| zgc:112300 | 5.6936244 | 1.9284333 | 3.2048896 | 2.1194211 |
| zgc:112315 | 2.2657896 | 1.8115802 | 1.4828268 | 1.5046839 |
| zgc:112355 | 8.7904217 | 1.025302 | 7.2557745 | 5.2884933 |
| zgc:112964 | 8.5038646 | 2.2178488 | 3.6967019 | 3.7515213 |
| zgc:123068 | 8.0154731 | 2.3619939 | 1.8488152 | 2.8111986 |
| zgc:123327 | 2.4787202 | 1.4787266 | 1.5500021 | 1.6598637 |
| zgc:136864 | 2.1046084 | 1.5046144 | 1.205199 | 1.3444249 |
| zgc:136892 | 6.1002746 | 1.3745326 | 2.6178653 | 1.8646836 |
| zgc:152911 | 4.8718948 | 1.7943512 | 3.0794621 | 2.8769978 |
| zgc:153258 | 3.3690405 | 1.2527005 | 2.8494046 | 2.4425589 |
| zgc:153284 | 4.2567832 | 1.3874524 | 2.3534601 | 2.5821941 |
| zgc:153723 | 4.7236915 | 1.9247283 | 1.4397381 | 2.2681364 |
| zgc:154054 | 4.4312064 | 1.4216744 | 2.7584775 | 2.3055025 |
| zgc:154093 | 2.0765336 | 1.39003 | 1.7958728 | 1.694216 |
| zgc:154164 | 8.1001426 | 2.0170161 | 4.5136358 | 2.1778817 |
| zgc:158343 | 2.4092213 | 6.2003779 | 2.4526073 | 5.6176705 |
| zgc:162184 | 3.6419853 | 1.7917396 | 1.2225276 | 2.6771616 |
| zgc:163030 | 4.8892778 | 1.8684292 | 3.4132106 | 2.6213112 |
| zgc:163083 | 9.4276641 | 2.6033797 | 5.4744481 | 8.5423254 |
| zgc:165571 | 7.0264198 | 1.931734 | 4.9524433 | 4.0144134 |
| zgc:171775 | 2.0805157 | 1.3113925 | 1.4187178 | 1.1815653 |
| zgc:171795 | 2.1341758 | 2.0289469 | 1.2584587 | 1.6691521 |
| zgc:171818 | 14.569228 | 4.2429878 | 5.5143621 | 4.2577513 |
| zgc:171957 | 3.8766915 | 1.9663034 | 2.5817293 | 2.2375173 |
| zgc:172075 | 4.2314915 | 1.6663718 | 1.3748686 | 1.7978244 |
| zgc:172260 | 4.2604938 | 2.6547766 | 2.528385 | 2.1212052 |
| zgc:173729 | 3.8719577 | 1.7031163 | 2.8985849 | 2.5318389 |
| zgc:173961 | 10.778484 | 3.0449114 | 2.221191 | 3.9723568 |
| zgc:174178 | 5.2762902 | 2.0506705 | 3.2287307 | 2.0442473 |
| zgc:174688 | 4.0154433 | 2.1872041 | 2.3352115 | 2.20741 |
| zgc:174689 | 6.4649988 | 2.2435701 | 4.0389715 | 3.1801676 |
| zgc:194246 | 2.808162 | 1.7003884 | 1.5038607 | 1.0401847 |
| zgc:194314 | 5.8325968 | 1.3185692 | 4.5452261 | 2.6905146 |
| zgc:198241 | 11.367108 | 14.757189 | 12.826547 | 5.2925293 |
| zgc:56530 | 2.6681268 | 2.039579 | 1.4755635 | 1.8423401 |
| zgc:63759 | 4.3145885 | 1.4759299 | 1.7459108 | 1.0416094 |
| zgc:63942 | 2.980227 | 1.0796344 | 1.4546144 | 1.3698307 |
| zgc:85789 | 2.0278606 | 1.0446883 | 1.3806879 | 1.5116232 |
| zgc:86896 | 2.0473036 | 1.6305912 | 2.5321225 | 1.5192508 |
| zgc:91849 | 2.7443304 | 1.0951721 | 1.1794368 | 2.4633033 |
| zgc:91887 | 4.7981626 | 1.0608887 | 2.0587607 | 2.3481626 |
| zgc:91908 | 2.4854939 | 1.0244505 | 1.6226312 | 1.3637284 |
| zgc:92380 | 2.232133 | 1.5397775 | 1.8553175 | 1.2724393 |
| zgc:92480 | 30.215373 | 10.676972 | 4.9307302 | 5.8797286 |
| zgc:92533 | 3.0634895 | 1.6202651 | 1.9442771 | 1.6935868 |
| zgc:92598 | 2.4522052 | 2.0216913 | 1.9035915 | 1.6707098 |
| zgc:92749 | 13.137807 | 3.4228266 | 6.235592 | 3.2990158 |
| zgc:92868 | 2.0821366 | 1.1702677 | 1.1577269 | 1.1933683 |
