# Supplementary material for: Transcriptome analysis reveals a ribosome constituents disorder involved in the RPL5 downregulated zebrafish model of Diamond-Blackfan anemia
Source: BMC Med Genomics. 2016 Mar 9;9:13. doi: 10.1186/s12920-016-0174-9 (PMC4785739; doi:10.1186/s12920-016-0174-9)
Supplement: Additional file 2: Table S2. — Up-regulated genes in RPL5 MO (fold-change > 2 and p-value < 0.05) showed opposite regulatory trend in other DBA zebrafish models. (DOC 39 kb) [file 12920_2016_174_MOESM2_ESM.doc]

**Table S2 Up-regulated genes in RPL5 MO (fold-change > 2 and p-value < 0.05) showed opposite regulatory trend in other DBA zebrafish models.**

| gene_symbol | fc_RPL5 | fc_RPS19 | fc_RPS24 | fc_RPL11 |
| --- | --- | --- | --- | --- |
| LOC568809 | 2.0902342 | 0.7578966 | 0.8189251 | 0.5558349 |
| LOC569000 | 2.0761684 | 0.9851736 | 0.6226967 | 0.4690982 |
| ces3 | 2.3426507 | 0.2281081 | 0.4304435 | 0.4543171 |
| crygm2d11 | 2.7218639 | 0.6791213 | 0.7004869 | 0.3637341 |
| crygm2d15 | 2.6533212 | 0.2436079 | 0.4852001 | 0.1792875 |
| crygm2d16 | 2.915679 | 0.754142 | 0.5252344 | 0.3482235 |
| crygm2d4 | 2.9550777 | 0.7751904 | 0.5524662 | 0.4651827 |
| crygm2d5 | 2.7823752 | 0.6322979 | 0.3957917 | 0.3424905 |
| crygm2d7 | 2.9067097 | 0.7300713 | 0.6424873 | 0.5667397 |
| crygm2d8 | 2.9944349 | 0.897668 | 0.8769687 | 0.551827 |
| ebi3 | 3.4737664 | 0.1078882 | 0.4569149 | 0.6208215 |
| elovl6 | 2.455047 | 0.2157517 | 0.3676508 | 0.4311512 |
| hpdb | 2.0581566 | 0.827795 | 0.5173756 | 0.7482549 |
| oat | 2.2241115 | 0.4502656 | 0.3151168 | 0.561666 |
| rbp4 | 2.1776053 | 0.7507244 | 0.7053753 | 0.724006 |
| zgc:110602 | 6.7283417 | 0.3213814 | 0.9157104 | 0.5298329 |
| zgc:162193 | 2.0123519 | 0.9063183 | 0.7484732 | 0.8214731 |
| zgc:162402 | 4.3786801 | 0.7713938 | 0.8073227 | 0.599003 |
| zgc:165347 | 2.2036264 | 0.6246987 | 0.551719 | 0.3972419 |
| zgc:171792 | 2.1093226 | 0.6940154 | 0.4838862 | 0.2775437 |
| zgc:86723 | 3.066954 | 0.7317852 | 0.5468263 | 0.3720781 |
