# Supplementary material for: Transcriptome analysis reveals a ribosome constituents disorder involved in the RPL5 downregulated zebrafish model of Diamond-Blackfan anemia
Source: BMC Med Genomics. 2016 Mar 9;9:13. doi: 10.1186/s12920-016-0174-9 (PMC4785739; doi:10.1186/s12920-016-0174-9)
Supplement: Additional file 3: Table S3. — Down-regulated genes in RPL5 MO (fold-change < 0.5 and p-value < 0.05) showed coordinated regulatory trend in other DBA zebrafish models. (DOC 976 kb) [file 12920_2016_174_MOESM3_ESM.doc]

**Table S3 Down-regulated genes in RPL5 MO (fold-change < 0.5 and p-value < 0.05) showed coordinated regulatory trend in other DBA zebrafish models.**

| gene_symbol | fc_RPL5 | fc_RPS19 | | fc_RPS24 | fc_RPL11 |
| --- | --- | --- | --- | --- | --- |
| LOC100000800 | 0.3439599 | 0.1334674 | | 0.1527644 | 0.39657 |
| LOC100002393 | 0.4721611 | 0.4155123 | | 0.407429 | 0.6319813 |
| LOC100002616 | 0.4637918 | 0.7661436 | | 0.1816132 | 0.5910123 |
| LOC100003866 | 0.3181458 | 0.3062255 | | 0.2597772 | 0.3980564 |
| LOC100005283 | 0.0397756 | 0 | | 0.1409183 | 0.0814636 |
| LOC100148281 | 0.3759021 | 0.9275063 | | 0.7796578 | 0.7311148 |
| LOC325449 | 0.4322972 | 0.5698755 | | 0.6168196 | 0.5314676 |
| LOC402838 | 0.4163113 | 0.7780851 | | 0.6048536 | 0.6872857 |
| LOC555453 | 0.3835498 | 0.4972867 | | 0.6221956 | 0.8160675 |
| LOC556480 | 0.2062756 | 0.1600797 | | 0.0458362 | 0.2642977 |
| LOC559147 | 0.124874 | 0.2230438 | | 0.1362476 | 0.3343319 |
| LOC561143 | 0.4012791 | 0.3578676 | | 0.409411 | 0.5048268 |
| LOC566022 | 0.3229615 | 0.8555786 | | 0.6871126 | 0.6175011 |
| LOC567595 | 0.2571796 | 0.2508725 | | 0.0655323 | 0.1884702 |
| LOC568087 | 0.3782531 | 0.6532635 | | 0.5274275 | 0.6785008 |
| LOC792509 | 0.4244952 | 0.1240823 | | 0.1901215 | 0.1744247 |
| LOC796180 | 0.4160126 | 0.5783034 | | 0.265398 | 0.5284648 |
| LOC797776 | 0.1075158 | 0.1839621 | | 0.022617 | 0 |
| acrc | 0.4193071 | 0.7169104 | | 0.8477797 | 0.7073315 |
| acta2 | 0.4366119 | 0.4941388 | | 0.4921049 | 0.2451025 |
| adar | 0.4303062 | 0.5324621 | | 0.4727004 | 0.4707135 |
| adarb1 | 0.4605257 | 0.2715779 | | 0.1917191 | 0.3622647 |
| adnp2b | 0.4506972 | 0.5530211 | | 0.4483741 | 0.4802651 |
| afmid | 0.3458384 | 0.682161 | | 0.4480461 | 0.4760092 |
| agfg1b | 0.4960545 | 0.880918 | | 0.6638887 | 0.7400532 |
| aggf1 | 0.34597 | 0.7556137 | | 0.9660288 | 0.6078597 |
| agmat | 0.1636224 | 0.7112496 | | 0.3854305 | 0.4464924 |
| agxt2 | 0.4435501 | 0.4532956 | | 0.4333486 | 0.4351715 |
| agxt2l1 | 0.3600692 | 0.3811291 | | 0.2503829 | 0.2400325 |
| agxta | 0.0813188 | 0.530239 | | 0.3409768 | 0.2911937 |
| ahr1b | 0.1235444 | 0.2172995 | | 0.1467566 | 0.3379111 |
| ahsa1 | 0.2918612 | 0.9866575 | | 0.7978857 | 0.8634361 |
| aldh1a3 | 0.1661029 | 0.3208594 | | 0.2372143 | 0.2741034 |
| alg3 | 0.3716068 | 0.9530239 | | 0.7202535 | 0.9258504 |
| alp | 0.4596754 | 0.5708482 | | 0.5545311 | 0.4979864 |
| ampd1 | 0.2307844 | 0.3177163 | | 0.2898883 | 0.1293529 |
| amph | 0.3962071 | 0.3347425 | | 0.2919035 | 0.4119008 |
| ankhb | 0.4253839 | 0.5137158 | | 0.4557689 | 0.585348 |
| ankhd1 | 0.4955448 | 0.7390758 | | 0.5391294 | 0.7357082 |
| ankmy2a | 0.3441529 | 0.8061322 | | 0.5970689 | 0.5984094 |
| ankrd10a | 0.4249556 | 0.5576037 | | 0.4331404 | 0.6321973 |
| ap2b1 | 0.4947099 | 0.7170406 | | 0.6561931 | 0.6407831 |
| apex1 | 0.4640453 | 0.7739745 | | 0.6772776 | 0.598629 |
| api5 | 0.4379747 | 0.860321 | | 0.6637583 | 0.7279189 |
| aplnrb | 0.3900811 | 0.4336411 | | 0.3131369 | 0.3121069 |
| aqr | 0.4793005 | 0.8360469 | | 0.8130884 | 0.7805605 |
| arf3a | 0.3885706 | 0.4182439 | | 0.2288551 | 0.3048538 |
| arglu1b | 0.4105602 | 0.7196391 | | 0.6858671 | 0.6512218 |
| arhgap10 | 0.4661995 | 0.9230928 | | 0.6349353 | 0.8030981 |
| arhgap12b | 0.3743339 | 0.425252 | | 0.4269435 | 0.4914703 |
| arhgef7a | 0.4766143 | 0.5080819 | | 0.4113789 | 0.4640468 |
| arid1aa | 0.4632792 | 0.6116555 | | 0.2924151 | 0.6839163 |
| arid2 | 0.4683879 | 0.5418956 | | 0.4132017 | 0.6324528 |
| arid4a | 0.3889202 | 0.4791794 | | 0.3870044 | 0.4843353 |
| arl13a | 0.1565818 | 0.1361094 | | 0.1388122 | 0.0962314 |
| arl16 | 0.3773583 | 0.9569283 | | 0.9334902 | 0.5146081 |
| arl3l2 | 0.1040272 | 0 | | 0.0914325 | 0.212609 |
| arntl2 | 0.4272478 | 0.734131 | | 0.6558882 | 0.424661 |
| arx | 0.4381871 | 0.8215631 | | 0.5953445 | 0.7587706 |
| asap2a | 0.4443944 | 0.3856536 | | 0.4243 | 0.4093204 |
| ascc1 | 0.3782867 | 0.8452773 | | 0.5022043 | 0.551183 |
| ascl1a | 0.1885608 | 0.8196137 | | 0.6025728 | 0.6282195 |
| ascl1b | 0.3173816 | 0.9781165 | | 0.5889131 | 0.6247479 |
| asic1b | 0.2908739 | 0.2298342 | | 0.2350641 | 0.3252689 |
| asns | 0.3976126 | 0.9905943 | | 0.7060931 | 0.8804517 |
| aspn | 0.4400899 | 0.4831353 | | 0.3054049 | 0.3397418 |
| atf6 | 0.4394622 | 0.4811481 | | 0.4700059 | 0.7491391 |
| atoh1b | 0.325404 | 0.5921044 | | 0.7691716 | 0.4975258 |
| atoh7 | 0.1141387 | 0.1240686 | | 0.0751227 | 0.3934837 |
| atp1a1a.5 | 0.4951503 | 0.8678614 | | 0.9599576 | 0.5394143 |
| atp1a3b | 0.4379672 | 0.8403494 | | 0.6422977 | 0.694314 |
| atp1b2a | 0.4316827 | 0.4682229 | | 0.2516186 | 0.4703089 |
| atp1b2b | 0.3065585 | 0.2156405 | | 0.0726031 | 0.146578 |
| atp2b1b | 0.1683962 | 0.0813051 | | 0.283545 | 0.3071064 |
| atp6v1b2 | 0.4492301 | 0.5674757 | | 0.4563153 | 0.5173693 |
| atrx | 0.4793299 | 0.4402634 | | 0.3727557 | 0.4677624 |
| atxn2l | 0.441272 | 0.8288566 | | 0.7417639 | 0.8134321 |
| atxn7l3 | 0.3044961 | 0.3724609 | | 0.2808568 | 0.3924031 |
| aven | 0.4480779 | 0.6503128 | | 0.7839132 | 0.642088 |
| axdnd1 | 0.1609985 | 0.619681 | | 0.1719365 | 0.3962004 |
| ba1l | 0.0814135 | 0 | | 0.1427809 | 0.496016 |
| bag4 | 0.4812047 | 0.8173442 | | 0.6799639 | 0.6597861 |
| barhl1.1 | 0.3430247 | 0.3986154 | | 0.2918174 | 0.3392428 |
| barhl1.2 | 0.466742 | 0.2140118 | | 0.2608079 | 0.3529757 |
| barhl2 | 0.4666785 | 0.3266872 | | 0.4053125 | 0.4139393 |
| barx1 | 0.3747789 | 0.1659481 | | 0.1439277 | 0.3001317 |
| bbs1 | 0.2356958 | 0.8962571 | | 0.6024541 | 0.6342311 |
| bbs7 | 0.4040134 | 0.9636984 | | 0.8316863 | 0.7138395 |
| bcdo2l | 0.1032662 | 0.7052695 | | 0.183275 | 0.4232148 |
| bcl7a | 0.4370663 | 0.5806813 | | 0.4632481 | 0.5642698 |
| bend3 | 0.4819852 | 0.7018369 | | 0.4188634 | 0.6286565 |
| bgna | 0.1970347 | 0.2752749 | | 0.1308413 | 0.1008768 |
| bgnb | 0.3199737 | 0.606982 | | 0.4109883 | 0.2733507 |
| bhlhe22 | 0.2738023 | 0.3400157 | | 0.253971 | 0.3265835 |
| bhlhe23 | 0.1479058 | 0.0795973 | | 0.0261613 | 0.1009336 |
| bin2a | 0.2086792 | 0.9933623 | | 0.4492708 | 0.447854 |
| bmi1a | 0.3776846 | 0.5900566 | | 0.4226869 | 0.6625116 |
| bmp3 | 0.4930596 | 0.4948838 | | 0.3809844 | 0.6690454 |
| bora | 0.391837 | 0.6236965 | | 0.6824688 | 0.5911756 |
| brca2 | 0.1998464 | 0.3485217 | | 0.1699469 | 0.3125362 |
| brd2a | 0.4614701 | 0.6833741 | | 0.3793708 | 0.5056161 |
| brd3a | 0.4089481 | 0.4779999 | | 0.3913337 | 0.4776689 |
| brd3b | 0.4361075 | 0.4850348 | | 0.3590652 | 0.5510973 |
| brd4 | 0.4642526 | 0.6064106 | | 0.3770473 | 0.5992235 |
| bri3bp | 0.4049425 | 0.4178982 | | 0.2627917 | 0.5347452 |
| brms1 | 0.2800595 | 0.3931712 | | 0.2693349 | 0.3864366 |
| bsx | 0.1307599 | 0.2234056 | | 0.1711471 | 0.3333898 |
| btbd10a | 0.4676051 | 0.4669545 | | 0.4857966 | 0.5933627 |
| btg3 | 0.4688941 | 0.7952681 | | 0.7013212 | 0.87669 |
| cab39 | 0.4372047 | 0.7360992 | | 0.3648976 | 0.6113068 |
| cabin1 | 0.4663706 | 0.4171177 | | 0.2864839 | 0.5352027 |
| cabp5b | 0.0577364 | 0.0358586 | | 0.2032428 | 0.059021 |
| cacnb4b | 0.4138623 | 0.5406993 | | 0.4330757 | 0.6096831 |
| cadm3 | 0.2294783 | 0.2585357 | | 0.1622385 | 0.4965467 |
| calb2a | 0.3566954 | 0.5589243 | | 0.5564582 | 0.4922262 |
| calb2b | 0.4480228 | 0.8179586 | | 0.7665081 | 0.5926432 |
| cald1l | 0.4977809 | 0.673118 | | 0.467764 | 0.5578691 |
| camsap2 | 0.4050056 | 0.3666505 | | 0.2004959 | 0.4211944 |
| capn3 | 0.3149845 | 0.4341745 | | 0.3991081 | 0.4269885 |
| caprin1a | 0.3883076 | 0.7931188 | | 0.6528845 | 0.7206815 |
| casp8ap2 | 0.3994482 | 0.3534687 | | 0.2274909 | 0.3052221 |
| casq1a | 0.3539929 | 0.824259 | | 0.8326584 | 0.7758318 |
| cbx1a | 0.3908051 | 0.6002089 | | 0.5765481 | 0.5713009 |
| cbx6a | 0.4559623 | 0.3248036 | | 0.3154735 | 0.4623679 |
| ccdc102a | 0.474748 | 0.5426186 | | 0.476501 | 0.496217 |
| ccdc6b | 0.3638883 | 0.5647771 | | 0.5185745 | 0.760431 |
| ccdc82 | 0.4336697 | 0.5261822 | | 0.5697645 | 0.5392599 |
| ccdc85ca | 0.4157699 | 0.3245331 | | 0.389627 | 0.35286 |
| ccna2 | 0.4655163 | 0.5990582 | | 0.6930959 | 0.5847775 |
| ccnb3 | 0.4966609 | 0.4995396 | | 0.4852152 | 0.5001595 |
| ccnd1 | 0.3016189 | 0.4622755 | | 0.3807002 | 0.4852221 |
| ccnd2a | 0.4305518 | 0.4672222 | | 0.3556003 | 0.4496137 |
| ccne1 | 0.332596 | 0.6484879 | | 0.6471321 | 0.5141179 |
| ccnf | 0.4348957 | 0.5713272 | | 0.6980654 | 0.7072473 |
| ccnt2a | 0.4773487 | 0.7235974 | | 0.6375426 | 0.7548915 |
| ccnt2b | 0.4392171 | 0.9816132 | | 0.7227595 | 0.8338119 |
| cd36 | 0.2660122 | 0.532228 | | 0.4710241 | 0.4236977 |
| cdc20 | 0.3913708 | 0.5833054 | | 0.5852111 | 0.5160237 |
| cdc23 | 0.4872649 | 0.815358 | | 0.7813842 | 0.7044495 |
| cdc27 | 0.4956386 | 0.764459 | | 0.6773835 | 0.715638 |
| cdc7 | 0.219381 | 0.4346033 | | 0.6384039 | 0.4814513 |
| cdc73 | 0.4015607 | 0.8684501 | | 0.8094689 | 0.9297616 |
| cdk11b | 0.4287013 | 0.8620175 | | 0.9595478 | 0.8140622 |
| cdk5r2a | 0.4297985 | 0.3959105 | | 0.2927597 | 0.3240324 |
| cdkn1bl | 0.4294171 | 0.799822 | | 0.5285085 | 0.6793791 |
| cdx1b | 0.0826732 | 0.4106524 | | 0.1832337 | 0.2963891 |
| celf3a | 0.4969285 | 0.3189379 | | 0.353277 | 0.5524855 |
| cenpo | 0.3358661 | 0.4930228 | | 0.493327 | 0.3955138 |
| cenpp | 0.3409839 | 0.5135746 | | 0.5696275 | 0.3746174 |
| cep128 | 0.2944166 | 0.6295646 | | 0.6549837 | 0.6037612 |
| cep70 | 0.432023 | 0.6284701 | | 0.5202188 | 0.5305614 |
| cep89 | 0.2998713 | 0.5235905 | | 0.2500583 | 0.7109469 |
| cetn3 | 0.4609188 | 0.4382638 | | 0.4648797 | 0.3743894 |
| cetp | 0.4565086 | 0.4849444 | | 0.8088161 | 0.4731405 |
| cfh | 0.4347444 | 0.1982373 | | 0.2650929 | 0.3099138 |
| cfhl4 | 0.4727613 | 0.2600624 | | 0.3653256 | 0.2824003 |
| chaf1a | 0.4371134 | 0.7676199 | | 0.8746377 | 0.5827593 |
| chd4a | 0.4082533 | 0.5430851 | | 0.4233579 | 0.5136256 |
| chd8 | 0.4049352 | 0.6510975 | | 0.5212465 | 0.6542003 |
| chek1 | 0.4263539 | 0.5379118 | | 0.6829612 | 0.5620172 |
| chkb | 0.3975102 | 0.6708041 | | 0.4728565 | 0.707256 |
| chrna6 | 0.1377792 | 0.6843708 | | 0.4886348 | 0.4233941 |
| chtf18 | 0.3596271 | 0.5492865 | | 0.622344 | 0.6116713 |
| chtopa | 0.4742349 | 0.5917654 | | 0.7046058 | 0.5964354 |
| cldn15a | 0.1687356 | 0.2821351 | | 0.1372248 | 0.3185404 |
| cldn15la | 0.3637629 | 0.6315924 | | 0.2119837 | 0.3330989 |
| cldna | 0.3685415 | 0.2734808 | | 0.1752525 | 0.3866681 |
| cnot10 | 0.4326009 | 0.838738 | | 0.8592275 | 0.8827241 |
| cnot3a | 0.3868966 | 0.6181964 | | 0.4412557 | 0.5214285 |
| col10a1 | 0.1886864 | 0.1852214 | | 0.0579544 | 0.1306277 |
| col11a2 | 0.2741266 | 0.4827751 | | 0.5026709 | 0.5767044 |
| col27a1a | 0.4194343 | 0.3438815 | | 0.1623153 | 0.3491226 |
| col2a1a | 0.3386799 | 0.3742458 | | 0.5540634 | 0.5688421 |
| col9a2 | 0.416912 | 0.3538335 | | 0.433721 | 0.5380206 |
| cp | 0.3327583 | 0.2332131 | | 0.2766826 | 0.3470878 |
| cplx2l | 0.4881004 | 0.9796007 | | 0.7168367 | 0.6317525 |
| cpne2 | 0.3509976 | 0.5964806 | | 0.3107417 | 0.4728855 |
| cratb | 0.4889983 | 0.9133171 | | 0.8254084 | 0.6654984 |
| crb2a | 0.3683494 | 0.5992362 | | 0.31147 | 0.5473 |
| creb1a | 0.4951186 | 0.4739755 | | 0.4933417 | 0.5631496 |
| crispld1b | 0.3503612 | 0.2919493 | | 0.2209134 | 0.2739526 |
| crmp1 | 0.4511866 | 0.2943701 | | 0.2715419 | 0.2429134 |
| crnkl1 | 0.4869062 | 0.8106674 | | 0.772034 | 0.7293015 |
| crsp7 | 0.3661463 | 0.6819767 | | 0.4361207 | 0.460529 |
| crx | 0.1377152 | 0.0444161 | | 0.044478 | 0.1722921 |
| cryaa | 0.3747123 | 0.6127632 | | 0.4300718 | 0.2811496 |
| cryabb | 0.1290623 | 0.0400654 | | 0.0572763 | 0.0661205 |
| cryba1b | 0.2543956 | 0.8074531 | | 0.6042569 | 0.5645958 |
| crybb1 | 0.19883 | 0.8901126 | | 0.5543503 | 0.616471 |
| csad | 0.4290439 | 0.2625673 | | 0.3581984 | 0.3192615 |
| cse1l | 0.4336734 | 0.8359206 | | 0.6511393 | 0.6394686 |
| cspg5b | 0.3610401 | 0.2751219 | | 0.2035649 | 0.2857202 |
| csrp1b | 0.0803874 | 0 | | 0.0702355 | 0 |
| csrp2bp | 0.4675696 | 0.7597668 | | 0.6494444 | 0.6685429 |
| cstf1 | 0.4591246 | 0.7845857 | | 0.7587708 | 0.7757658 |
| ctbp1 | 0.444185 | 0.6641342 | | 0.5671245 | 0.7280175 |
| ctcf | 0.4866422 | 0.530314 | | 0.489371 | 0.6235668 |
| ctdp1 | 0.3784932 | 0.7327562 | | 0.5669459 | 0.6841576 |
| ctnnd2b | 0.3929777 | 0.4867148 | | 0.4813996 | 0.6077109 |
| ctr9 | 0.4900434 | 0.9511093 | | 0.7976718 | 0.8391649 |
| cul1a | 0.4440245 | 0.7589353 | | 0.5868237 | 0.6455082 |
| cul4b | 0.4950684 | 0.6848845 | | 0.5247562 | 0.6740405 |
| cwf19l1 | 0.4922377 | 0.8742279 | | 0.761428 | 0.9264168 |
| cwf19l2 | 0.4459544 | 0.5998589 | | 0.6898047 | 0.6228468 |
| cxcl12b | 0.3955885 | 0.2835916 | | 0.4039536 | 0.4211395 |
| cxxc1 | 0.4406305 | 0.7471679 | | 0.7787955 | 0.6381511 |
| cygb2 | 0.1829593 | 0.113618 | | 0.1615181 | 0.1247937 |
| cyp1b1 | 0.4065212 | 0.2407255 | | 0.1840955 | 0.4017889 |
| cyp1d1 | 0.349916 | 0.7332164 | | 0.6524156 | 0.6005961 |
| cyp2aa8 | 0.4678286 | 0.5164855 | | 0.303836 | 0.4863656 |
| cyp2p9 | 0.2006776 | 0.1607462 | | 0.2800544 | 0.4113034 |
| cyp2x10.2 | 0.1930068 | 0.250715 | | 0.1865928 | 0.0718456 |
| dachb | 0.3649691 | 0.5313214 | | 0.5029906 | 0.5674879 |
| dachc | 0.4397314 | 0.6749448 | | 0.4175834 | 0.6008234 |
| dachd | 0.4648917 | 0.4259017 | | 0.5011106 | 0.4793567 |
| dact1 | 0.4512597 | 0.4154299 | | 0.3546721 | 0.4288832 |
| dact2 | 0.4086803 | 0.5184591 | | 0.5217068 | 0.5951348 |
| dag1 | 0.4762841 | 0.8527798 | | 0.6918837 | 0.854784 |
| daxx | 0.4388406 | 0.6729247 | | 0.6204284 | 0.6956806 |
| dbh | 0.1347754 | 0.1394684 | | 0.0398349 | 0.4141727 |
| dbx1b | 0.2650705 | 0.9582514 | | 0.5349738 | 0.6102325 |
| dck | 0.4498181 | 0.3673555 | | 0.3802508 | 0.3063416 |
| ddc | 0.3013077 | 0.2754744 | | 0.2237186 | 0.1802807 |
| ddx42 | 0.4745591 | 0.8535661 | | 0.7708949 | 0.7507365 |
| decr2 | 0.4423762 | 0.5525477 | | 0.3600347 | 0.4951937 |
| depdc1a | 0.4060633 | 0.3723197 | | 0.3445278 | 0.4227169 |
| desmb | 0.1216273 | 0.0755195 | | 0.1077716 | 0.0622828 |
| dhx15 | 0.476245 | 0.9937378 | | 0.9830273 | 0.9748642 |
| dhx16 | 0.4533976 | 0.8406992 | | 0.699319 | 0.7647233 |
| dhx38 | 0.4791976 | 0.5645195 | | 0.3979935 | 0.4803902 |
| dhx9 | 0.4991375 | 0.6614169 | | 0.5938597 | 0.7470262 |
| dicer1 | 0.4612976 | 0.5843656 | | 0.270414 | 0.5861627 |
| dicp3.3 | 0.183909 | 0.0761582 | | 0.2687714 | 0.0626049 |
| dido1 | 0.4571878 | 0.4415231 | | 0.284515 | 0.489486 |
| diexf | 0.3618076 | 0.9836728 | | 0.8654045 | 0.8699875 |
| dkk3 | 0.1611142 | 0.7732421 | | 0.4765286 | 0.5089519 |
| dlc | 0.4622717 | 0.5303172 | | 0.4043944 | 0.9010102 |
| dlgap5 | 0.4432855 | 0.6686657 | | 0.7339741 | 0.7673512 |
| dlx2a | 0.4410407 | 0.6465509 | | 0.3962792 | 0.4923166 |
| dlx6a | 0.2771223 | 0.3951722 | | 0.49843 | 0.283543 |
| dmtf1 | 0.4631978 | 0.5511692 | | 0.7059712 | 0.757458 |
| dnaja1l | 0.4709006 | 0.387529 | | 0.5115215 | 0.6377023 |
| dnajb11 | 0.4519358 | 0.4450465 | | 0.3081235 | 0.4063235 |
| dnm1b | 0.3716944 | 0.2192237 | | 0.1238792 | 0.2762147 |
| dnmt1 | 0.397291 | 0.6178371 | | 0.6122036 | 0.5979598 |
| dnmt4 | 0.3549782 | 0.7712897 | | 0.7305688 | 0.5967748 |
| dnmt5 | 0.3369886 | 0.5020292 | | 0.3822301 | 0.5341973 |
| dnmt7 | 0.4562665 | 0.4512498 | | 0.4005641 | 0.4540259 |
| dpf1 | 0.3832936 | 0.5355657 | | 0.427738 | 0.5304959 |
| dpys | 0.3138024 | 0.8627605 | | 0.5979196 | 0.5055136 |
| dpysl5a | 0.3921347 | 0.4740953 | | 0.3369092 | 0.4138541 |
| dtnbb | 0.1032401 | 0.6409834 | | 0.4582238 | 0.1057865 |
| dync1li1 | 0.4853487 | 0.7138079 | | 0.6322608 | 0.6013331 |
| e2f4 | 0.3414227 | 0.6524045 | | 0.5184013 | 0.5829134 |
| e2f7 | 0.3763419 | 0.3811874 | | 0.4585521 | 0.4621187 |
| ebf3 | 0.4358689 | 0.2827596 | | 0.2504468 | 0.3849283 |
| ect2 | 0.2995686 | 0.9382076 | | 0.8572683 | 0.5638675 |
| edc3 | 0.4060496 | 0.4775979 | | 0.3300921 | 0.5553332 |
| edc4 | 0.4034723 | 0.6376412 | | 0.4542515 | 0.6013585 |
| eef1a1a | 0.3156168 | 0.2407473 | | 0.2361067 | 0.3141484 |
| ehmt1a | 0.3860121 | 0.7070348 | | 0.6359273 | 0.5233742 |
| ehmt2 | 0.4312099 | 0.8658099 | | 0.8768985 | 0.8103538 |
| eif2ak1 | 0.4251029 | 0.7327233 | | 0.5542297 | 0.7051513 |
| eif2ak3 | 0.4888383 | 0.4081969 | | 0.4547545 | 0.5368771 |
| eif3ja | 0.4942903 | 0.8727253 | | 0.653221 | 0.9297086 |
| eif4bb | 0.3433922 | 0.9248713 | | 0.8175122 | 0.8446547 |
| elovl4b | 0.1213927 | 0.4082361 | | 0.476272 | 0.4147037 |
| emilin1a | 0.4552305 | 0.5267914 | | 0.4309231 | 0.5302185 |
| emilin1b | 0.468325 | 0.4523614 | | 0.4092751 | 0.455779 |
| emx2 | 0.4063615 | 0.240427 | | 0.274717 | 0.3414351 |
| eng2a | 0.3781794 | 0.5763342 | | 0.5118353 | 0.4881793 |
| enpp2 | 0.3706576 | 0.2691433 | | 0.113504 | 0.3800297 |
| entpd3 | 0.278709 | 0.3460919 | | 0.4217983 | 0.2519448 |
| epc2 | 0.4613746 | 0.8592935 | | 0.7824901 | 0.83297 |
| ephb2b | 0.4458271 | 0.5502678 | | 0.5905086 | 0.6451462 |
| epyc | 0.4097813 | 0.6213694 | | 0.6855979 | 0.8155712 |
| ercc4 | 0.4770302 | 0.540955 | | 0.4584971 | 0.4956566 |
| ercc6l | 0.4989793 | 0.4189892 | | 0.3566065 | 0.4228194 |
| eri1 | 0.4295275 | 0.8162063 | | 0.595239 | 0.7258145 |
| erlec1 | 0.464949 | 0.4188179 | | 0.4767679 | 0.4958753 |
| esco2 | 0.4324495 | 0.5424409 | | 0.6782984 | 0.665513 |
| etaa1 | 0.3900667 | 0.5624622 | | 0.6483359 | 0.4384602 |
| exo1 | 0.3981454 | 0.4416843 | | 0.4757847 | 0.4014351 |
| eya4 | 0.353368 | 0.8649385 | | 0.9579893 | 0.7571429 |
| ezh2 | 0.3663768 | 0.7483339 | | 0.5404129 | 0.5926678 |
| ezrl | 0.4034049 | 0.6875646 | | 0.3817957 | 0.4549859 |
| f7i | 0.4001389 | 0.0728573 | | 0.3024748 | 0.2599165 |
| fabp10a | 0.1735414 | 0.0540569 | | 0.0720334 | 0.2620839 |
| fam168a | 0.4968372 | 0.4514195 | | 0.2291058 | 0.4591409 |
| fam212aa | 0.469839 | 0.491765 | | 0.4759016 | 0.4509731 |
| fam54b | 0.4499842 | 0.7158053 | | 0.744444 | 0.5772365 |
| fam58a | 0.4358689 | 0.7085851 | | 0.6466193 | 0.687578 |
| fam60al | 0.4704636 | 0.6908573 | | 0.6541941 | 0.6239598 |
| fam76b | 0.4419844 | 0.7577471 | | 0.7612873 | 0.8308204 |
| fam82a2 | 0.3381592 | 0.7246094 | | 0.8395858 | 0.7875829 |
| fam98b | 0.4201958 | 0.8254594 | | 0.8242867 | 0.5262684 |
| fan1 | 0.4578528 | 0.8704709 | | 0.7081693 | 0.6573095 |
| fancd2 | 0.4666858 | 0.6272059 | | 0.6136569 | 0.6391329 |
| fbxl14b | 0.4510716 | 0.5558347 | | 0.4919786 | 0.5986567 |
| fbxo11a | 0.4612967 | 0.6058844 | | 0.4820305 | 0.6842754 |
| fen1 | 0.4840442 | 0.7711757 | | 0.7219419 | 0.5762104 |
| fezf1 | 0.2754252 | 0.2992608 | | 0.5986658 | 0.7054372 |
| fezf2 | 0.2956423 | 0.3530307 | | 0.3924899 | 0.5471686 |
| fgd | 0.4391033 | 0.6738622 | | 0.6228839 | 0.7030156 |
| fgf10a | 0.4403418 | 0.7097977 | | 0.5632706 | 0.5551914 |
| fgf13b | 0.44864 | 0.4548665 | | 0.3322212 | 0.4246199 |
| fgfrl1a | 0.4362354 | 0.4408268 | | 0.4214612 | 0.56335 |
| fhl2b | 0.3102122 | 0.0917221 | | 0.0785142 | 0.1361504 |
| fhla | 0.400646 | 0.95192 | | 0.5230559 | 0.7842633 |
| fkbp4 | 0.4488569 | 0.4514682 | | 0.6153034 | 0.6760204 |
| fkbp9 | 0.4363262 | 0.3017197 | | 0.5002589 | 0.5889803 |
| flrt3 | 0.4636786 | 0.5125934 | | 0.3426184 | 0.6234887 |
| flvcr1 | 0.3403309 | 0.5635428 | | 0.3763135 | 0.7258179 |
| foxa2 | 0.3367839 | 0.3286145 | | 0.2981998 | 0.3530619 |
| foxc1a | 0.4723673 | 0.4567456 | | 0.5280872 | 0.5501234 |
| foxc1b | 0.3234483 | 0.3658324 | | 0.4170211 | 0.4638561 |
| foxd1 | 0.2985296 | 0.256328 | | 0.2115035 | 0.3319553 |
| foxd1l | 0.2992681 | 0.7443923 | | 0.7905964 | 0.8331861 |
| foxf1 | 0.331783 | 0.4609149 | | 0.3017779 | 0.3229373 |
| foxf2a | 0.3819976 | 0.2340831 | | 0.2489083 | 0.4218824 |
| foxg1a | 0.3191616 | 0.3782938 | | 0.48219 | 0.4714879 |
| foxg1b | 0.1352201 | 0.1793576 | | 0.1118218 | 0.4786146 |
| foxi1 | 0.2418803 | 0.7269482 | | 0.436443 | 0.5942471 |
| foxi2 | 0.1481596 | 0.4370408 | | 0.294161 | 0.416811 |
| foxj1b | 0.327544 | 0.7866501 | | 0.753194 | 0.3779655 |
| foxk2 | 0.4729032 | 0.916194 | | 0.8685445 | 0.7906597 |
| foxl1 | 0.2819076 | 0.2250487 | | 0.2319997 | 0.2474873 |
| foxm1 | 0.3699921 | 0.6057533 | | 0.5586276 | 0.5542339 |
| foxn4 | 0.2261464 | 0.3743916 | | 0.3506809 | 0.522985 |
| fzd10 | 0.381825 | 0.8376538 | | 0.6107168 | 0.6501955 |
| fzd5 | 0.107646 | 0.2974254 | | 0.1622392 | 0.2866836 |
| fzd7a | 0.4873536 | 0.814159 | | 0.5940433 | 0.819251 |
| fzd8a | 0.2866177 | 0.4704695 | | 0.2084334 | 0.3650105 |
| fzd9b | 0.3518075 | 0.5342688 | | 0.2928044 | 0.3038315 |
| g2e3 | 0.477648 | 0.5101814 | | 0.4603536 | 0.472659 |
| gabpb2b | 0.3337143 | 0.4292773 | | 0.3681958 | 0.4755438 |
| galntl4a | 0.0688827 | 0.1283062 | | 0.4886506 | 0.2822447 |
| gap43 | 0.4873358 | 0.4938367 | | 0.4078402 | 0.3611358 |
| gas1a | 0.4502234 | 0.415716 | | 0.3579546 | 0.4161744 |
| gas1b | 0.3699692 | 0.4976446 | | 0.3996871 | 0.5328628 |
| gcgb | 0.1447187 | 0.1499557 | | 0.1927823 | 0.2610238 |
| gemin4 | 0.4926627 | 0.8202193 | | 0.8472932 | 0.7980074 |
| gfra1a | 0.2727684 | 0.5980208 | | 0.5622258 | 0.3931547 |
| gh1 | 0.1038807 | 0.1720433 | | 0.0304905 | 0.1416114 |
| gle1 | 0.3430575 | 0.8228453 | | 0.8362387 | 0.8324314 |
| gli1 | 0.3327544 | 0.8951074 | | 0.5877659 | 0.6574219 |
| glrbb | 0.3973515 | 0.4994156 | | 0.301202 | 0.4469136 |
| gnao1b | 0.3912791 | 0.3961665 | | 0.368718 | 0.3824287 |
| gnat2 | 0.3133056 | 0.3919306 | | 0.3871338 | 0.6015697 |
| gnb3a | 0.1058198 | 0.1159462 | | 0.0827893 | 0.076515 |
| gnb5a | 0.2159724 | 0.4381181 | | 0.3050973 | 0.44195 |
| gng13b | 0.2393652 | 0.6494057 | | 0.3845707 | 0.516526 |
| gpd1l | 0.433202 | 0.7470882 | | 0.4834143 | 0.6112883 |
| gpr22 | 0.2161168 | 0.3568607 | | 0.1915232 | 0.2705951 |
| gpr98 | 0.4442978 | 0.7569965 | | 0.3616946 | 0.7718414 |
| gps2 | 0.4716224 | 0.7033676 | | 0.7563078 | 0.6393809 |
| grip2a | 0.3267448 | 0.4837107 | | 0.5367592 | 0.6442748 |
| gsk3aa | 0.4873798 | 0.7024232 | | 0.8355089 | 0.7984441 |
| gsk3b | 0.4884357 | | 0.5144223 | 0.4302606 | 0.6459357 |
| gsx1 | 0.3010271 | | 0.6468658 | 0.6368704 | 0.8652664 |
| gtf2e1 | 0.4469026 | | 0.5914982 | 0.6335192 | 0.7135161 |
| gtf2f1 | 0.3468409 | | 0.560209 | 0.6003763 | 0.480084 |
| gtpbp2 | 0.4110432 | | 0.3791799 | 0.489308 | 0.408995 |
| gucy1b3 | 0.3015351 | | 0.4011992 | 0.2862678 | 0.2867612 |
| habp2 | 0.2775539 | | 0.1680279 | 0.2951011 | 0.1989782 |
| hand2 | 0.3651164 | | 0.3705176 | 0.2917216 | 0.3118626 |
| has2 | 0.2786374 | | 0.5881484 | 0.2230138 | 0.3713474 |
| hat1 | 0.4931353 | | 0.776545 | 0.7229791 | 0.7044769 |
| haus3 | 0.3934589 | | 0.4921041 | 0.5266213 | 0.4324197 |
| hbaa1 | 0.1205075 | | 0.1474457 | 0.1009472 | 0.7186402 |
| hcfc1a | 0.3703049 | | 0.561347 | 0.4264485 | 0.5811559 |
| hcfc1b | 0.4946178 | | 0.5101486 | 0.3765136 | 0.5080701 |
| hdgfrp2 | 0.3876018 | | 0.6536473 | 0.6134867 | 0.6148827 |
| hells | 0.2894288 | | 0.461106 | 0.4003069 | 0.3986711 |
| helz | 0.4454468 | | 0.3751551 | 0.5201409 | 0.6008316 |
| her6 | 0.349787 | | 0.6545956 | 0.5165175 | 0.48263 |
| hes2.2 | 0.1733981 | | 0.2038587 | 0.1197291 | 0.2974213 |
| hexim1 | 0.4920677 | | 0.643922 | 0.5325191 | 0.5574127 |
| hey1 | 0.4266247 | | 0.4395841 | 0.3088286 | 0.3340006 |
| hhip | 0.4306847 | | 0.9393963 | 0.6115585 | 0.7695897 |
| hirip3 | 0.408251 | | 0.3851033 | 0.5566076 | 0.463697 |
| hmga1b | 0.3729862 | | 0.304768 | 0.1646954 | 0.2942636 |
| hmga2 | 0.4150202 | | 0.5799071 | 0.3158809 | 0.5451667 |
| hmmr | 0.4115021 | | 0.5791792 | 0.5982952 | 0.5830024 |
| hmx1 | 0.1901908 | | 0.389932 | 0.365118 | 0.4655259 |
| hmx3 | 0.4657462 | | 0.3262487 | 0.2966 | 0.3486756 |
| hmx4 | 0.1939097 | | 0.5231869 | 0.360239 | 0.4207833 |
| hnrnpa3 | 0.3916727 | | 0.4924943 | 0.3543204 | 0.4441498 |
| hnrnph1 | 0.4789341 | | 0.740817 | 0.7411221 | 0.5637177 |
| hnrnpu | 0.4390174 | | 0.6381932 | 0.5411204 | 0.5999616 |
| hnrnpul1 | 0.4434142 | | 0.6494028 | 0.5378898 | 0.5780195 |
| hoxb4a | 0.3583906 | | 0.3071831 | 0.3968315 | 0.3272045 |
| hpca | 0.4529777 | | 0.5592913 | 0.5339052 | 0.5619485 |
| hpda | 0.083413 | | 0.0684859 | 0.0610666 | 0.0847159 |
| hprt1l | 0.4519912 | | 0.4820443 | 0.6550262 | 0.5780815 |
| hsd11b2 | 0.3967301 | | 0.3429333 | 0.2549388 | 0.33157 |
| hsd17b12b | 0.4992341 | | 0.7061392 | 0.5632254 | 0.6128747 |
| hsp47 | 0.2302586 | | 0.3997257 | 0.7133108 | 0.579132 |
| hsp90b1 | 0.4806059 | | 0.2023059 | 0.2994265 | 0.2546425 |
| hspa5 | 0.4278361 | | 0.2800328 | 0.4387189 | 0.5271055 |
| htatsf1 | 0.3820914 | | 0.7262146 | 0.7290727 | 0.6956015 |
| hyou1 | 0.3390109 | | 0.510945 | 0.3773336 | 0.4870115 |
| ift172 | 0.4718064 | | 0.5831118 | 0.4397844 | 0.5167809 |
| ift81 | 0.359009 | | 0.4817035 | 0.4017893 | 0.3799528 |
| igsf21b | 0.275215 | | 0.2029071 | 0.35134 | 0.4230549 |
| il1rapl1a | 0.2779092 | | 0.7475851 | 0.4794856 | 0.4418252 |
| ing1 | 0.4384041 | | 0.669738 | 0.5273164 | 0.5851343 |
| inhbaa | 0.3495159 | | 0.2398191 | 0.2128901 | 0.4527344 |
| inhbb | 0.4328743 | | 0.5438685 | 0.375938 | 0.4438182 |
| inpp5e | 0.4816435 | | 0.5750366 | 0.4281917 | 0.4810663 |
| ins | 0.1966722 | | 0.2249837 | 0.1290223 | 0.2573286 |
| insm1a | 0.326327 | | 0.4264231 | 0.3727166 | 0.6319374 |
| insm1b | 0.2863747 | | 0.7139407 | 0.6570221 | 0.7414913 |
| ints2 | 0.4933997 | | 0.6741978 | 0.5043013 | 0.6142014 |
| ints3 | 0.4716193 | | 0.7275488 | 0.6003377 | 0.6830339 |
| ints8 | 0.4566724 | | 0.732727 | 0.6774625 | 0.7352226 |
| ints9 | 0.4952405 | | 0.6618902 | 0.6916004 | 0.716719 |
| invs | 0.4134465 | | 0.9297908 | 0.95192 | 0.9840833 |
| ipo9 | 0.4579036 | | 0.8447121 | 0.7435242 | 0.7813316 |
| iqca1 | 0.2578124 | | 0.8460228 | 0.6548287 | 0.5285522 |
| irx1b | 0.3882517 | | 0.7151187 | 0.5749953 | 0.5646229 |
| irx5a | 0.4098611 | | 0.6362049 | 0.5543615 | 0.5579706 |
| irx7 | 0.3208625 | | 0.7211907 | 0.2017661 | 0.3280314 |
| isl1 | 0.3105161 | | 0.3217553 | 0.2547957 | 0.4059759 |
| isl2a | 0.4541999 | | 0.5349564 | 0.5201546 | 0.4982735 |
| isl2b | 0.4444662 | | 0.3501333 | 0.4109243 | 0.4482485 |
| itga11a | 0.2795566 | | 0.7664603 | 0.4535073 | 0.6373236 |
| jam3b | 0.4851075 | | 0.4894682 | 0.3449144 | 0.5523536 |
| kat7 | 0.3588284 | | 0.768923 | 0.7673834 | 0.8047958 |
| kat8 | 0.387446 | | 0.7745839 | 0.5312575 | 0.5847184 |
| kbtbd12 | 0.4573375 | | 0.8590988 | 0.9530249 | 0.7270728 |
| khsrp | 0.4559893 | | 0.8453772 | 0.7304498 | 0.7119487 |
| kiaa0586 | 0.3881153 | | 0.4998479 | 0.6995296 | 0.6033626 |
| kiaa0907 | 0.4616933 | | 0.6826341 | 0.7965542 | 0.9620182 |
| kif11 | 0.3830141 | | 0.6032573 | 0.6492598 | 0.594654 |
| kif14 | 0.4096698 | | 0.4768362 | 0.4563656 | 0.5673023 |
| kif18a | 0.3735646 | | 0.6149854 | 0.8071338 | 0.7147253 |
| kif2c | 0.4928607 | | 0.72913 | 0.6771725 | 0.7655432 |
| kifap3 | 0.4885767 | | 0.5599432 | 0.3414265 | 0.6372727 |
| kin | 0.4162507 | | 0.830518 | 0.4421411 | 0.5747647 |
| klc1a | 0.4391435 | | 0.4304996 | 0.389835 | 0.3473535 |
| klhdc4 | 0.4392745 | | 0.7850446 | 0.8425807 | 0.7753918 |
| kmo | 0.1986773 | | 0.4194124 | 0.176201 | 0.2035273 |
| kpna4 | 0.4452573 | | 0.7970146 | 0.8746593 | 0.7722303 |
| krcp | 0.4449694 | | 0.328268 | 0.4567006 | 0.3740372 |
| kri1 | 0.3892395 | | 0.9706473 | 0.8186871 | 0.8943493 |
| l3mbtl2 | 0.3737466 | | 0.8027751 | 0.7601976 | 0.6121537 |
| laptm4b | 0.4794075 | | 0.284172 | 0.2478695 | 0.4123491 |
| larp7 | 0.4787862 | | 0.6062815 | 0.4776735 | 0.6518024 |
| lbx2 | 0.466947 | | 0.6801353 | 0.3942005 | 0.4915624 |
| lcmt1 | 0.3274346 | | 0.9210792 | 0.5999594 | 0.6741849 |
| lctla | 0.2457482 | | 0.4424676 | 0.3273271 | 0.2014657 |
| ldb1a | 0.4142546 | | 0.500932 | 0.4489214 | 0.6048505 |
| ldb2b | 0.4131549 | | 0.3916228 | 0.2138954 | 0.3603702 |
| ldlr | 0.2062262 | | 0.5889433 | 0.2933742 | 0.3805297 |
| leo1 | 0.4435243 | | 0.7663846 | 0.7591331 | 0.6589232 |
| lgsn | 0.1290178 | | 0.1802291 | 0.0859232 | 0 |
| lhfpl2b | 0.4768544 | | 0.3621871 | 0.5540317 | 0.4184956 |
| lhx2b | 0.3877054 | | 0.2535462 | 0.1633947 | 0.3520782 |
| lhx3 | 0.1832935 | | 0.2403188 | 0.1076183 | 0.5205825 |
| lhx6 | 0.3890634 | | 0.1565485 | 0.1282548 | 0.2364142 |
| lhx9 | 0.433184 | | 0.3303768 | 0.3193363 | 0.3595591 |
| lim2.3 | 0.2502549 | | 0.8682366 | 0.6521755 | 0.5664636 |
| lim2.4 | 0.3084992 | | 0.7223571 | 0.6808184 | 0.5909188 |
| lim2.5 | 0.1567735 | | 0.9090264 | 0.182606 | 0.159937 |
| lin7b | 0.3518196 | | 0.4119618 | 0.3911494 | 0.3987161 |
| lingo4b | 0.1476976 | | 0.2292672 | 0.1962951 | 0 |
| lix1l | 0.4502778 | | 0.3998201 | 0.260586 | 0.4347014 |
| llgl1 | 0.4673511 | | 0.6295914 | 0.6757674 | 0.6540947 |
| lmbrd2b | 0.3567838 | | 0.3827433 | 0.3512652 | 0.3787679 |
| lmnb1 | 0.4706685 | | 0.7825783 | 0.7149401 | 0.6622606 |
| lmo4b | 0.4084328 | | 0.3616035 | 0.314938 | 0.3943809 |
| lnx1 | 0.4801366 | | 0.7416268 | 0.9844335 | 0.6476572 |
| lppr3b | 0.4513417 | | 0.7060121 | 0.641852 | 0.6121514 |
| lrig3 | 0.471151 | | 0.8088606 | 0.7806779 | 0.9280741 |
| lrp2a | 0.3997663 | | 0.886767 | 0.548219 | 0.9336375 |
| lsm14ab | 0.4892595 | | 0.9318884 | 0.6581245 | 0.8799383 |
| ltbp3 | 0.3892344 | | 0.4452693 | 0.3223509 | 0.5590814 |
| ltk | 0.2846556 | | 0.3398185 | 0.3318029 | 0.3369289 |
| mab21l1 | 0.3894693 | | 0.2577014 | 0.2186045 | 0.267369 |
| mad1l1 | 0.3504192 | | 0.8652677 | 0.7149818 | 0.6122604 |
| maf | 0.4219506 | | 0.7669652 | 0.3675098 | 0.3835186 |
| mafa | 0.3867865 | | 0.7000377 | 0.6405903 | 0.6649037 |
| magi2 | 0.3357771 | | 0.5419594 | 0.0398776 | 0.3673368 |
| marcksa | 0.4228446 | | 0.3938381 | 0.2949992 | 0.4049133 |
| mat2ab | 0.3888373 | | 0.7148757 | 0.6376387 | 0.6130547 |
| matn1 | 0.1593003 | | 0.0893353 | 0.0547053 | 0.0789689 |
| matr3l | 0.4217212 | | 0.6193583 | 0.5586968 | 0.640647 |
| mb | 0.2605127 | | 0.3625105 | 0.3958507 | 0.3898718 |
| mcm10 | 0.4525499 | | 0.6310025 | 0.5474239 | 0.5855805 |
| mcm3 | 0.3780689 | | 0.373068 | 0.7257356 | 0.5181798 |
| mcm5 | 0.4821495 | | 0.4927727 | 0.7266224 | 0.589489 |
| mcm6 | 0.4781279 | | 0.4614234 | 0.5548948 | 0.5354374 |
| mcmbp | 0.4766768 | | 0.7188358 | 0.8685653 | 0.7972258 |
| mcoln1 | 0.1728676 | | 0.4780445 | 0.3955811 | 0.3001241 |
| mdm4 | 0.4625263 | | 0.2892484 | 0.3934208 | 0.3521966 |
| med14 | 0.446365 | | 0.7959302 | 0.6 | 0.693768 |
| med15 | 0.4696347 | | 0.7985101 | 0.5768977 | 0.7257305 |
| med18 | 0.4754819 | | 0.9570463 | 0.7397058 | 0.7685574 |
| med23 | 0.4964997 | | 0.7943427 | 0.737296 | 0.8183895 |
| med24 | 0.4186118 | | 0.6728681 | 0.5591436 | 0.7753893 |
| meis2a | 0.4958678 | | 0.4616909 | 0.4382509 | 0.5561909 |
| melk | 0.3969473 | | 0.4182016 | 0.4971031 | 0.5979744 |
| men1 | 0.3629551 | | 0.4530476 | 0.458338 | 0.4738303 |
| mepce | 0.3192279 | | 0.474896 | 0.3663748 | 0.5633979 |
| metap2a | 0.1504472 | | 0.3840839 | 0.6053193 | 0.6157801 |
| metrn | 0.4625258 | | 0.7675962 | 0.561066 | 0.7826153 |
| mettl14 | 0.4478882 | 0.8220176 | | 0.7860424 | 0.8441509 |
| mettl3 | 0.3505618 | 0.7618139 | | 0.6892101 | 0.5433838 |
| mettl6 | 0.4135031 | 0.8359731 | | 0.5039655 | 0.6118021 |
| mfap1 | 0.4816727 | 0.7159626 | | 0.6390177 | 0.6495044 |
| mgrn1b | 0.493703 | 0.4018643 | | 0.1901766 | 0.320513 |
| mib | 0.4582496 | 0.6080148 | | 0.761033 | 0.8602137 |
| mier1a | 0.4006411 | 0.5166249 | | 0.4274454 | 0.5170979 |
| mipa | 0.2161646 | 0.5419622 | | 0.3549067 | 0.3153646 |
| mipb | 0.2637697 | 0.5226621 | | 0.5030157 | 0.4312566 |
| mllt3 | 0.4875602 | 0.6181406 | | 0.5427065 | 0.7101405 |
| mmrn2a | 0.3962526 | 0.6627795 | | 0.3445934 | 0.6455882 |
| mms22l | 0.3422747 | 0.5884064 | | 0.6305551 | 0.5850633 |
| morc2 | 0.4478908 | 0.8898431 | | 0.6565381 | 0.6729793 |
| mpp6b | 0.4122162 | 0.5724151 | | 0.6213862 | 0.6085202 |
| msh6 | 0.3985034 | 0.6307498 | | 0.7248389 | 0.5681388 |
| msi1 | 0.3738786 | 0.5618259 | | 0.4705516 | 0.5630984 |
| msxe | 0.4199937 | 0.4980094 | | 0.5116617 | 0.5071938 |
| mta2 | 0.4628128 | 0.6023185 | | 0.5227739 | 0.6870463 |
| mthfd2 | 0.4494527 | 0.6676327 | | 0.5174034 | 0.747173 |
| mtx3 | 0.3850409 | 0.2731967 | | 0.2200155 | 0.4087744 |
| mxtx1 | 0.1033658 | 0.6418037 | | 0.2748316 | 0.5293469 |
| c-mybl2 | 0.4236972 | 0.5454782 | | 0.6103477 | 0.6858238 |
| mycn | 0.3594729 | 0.9067188 | | 0.5953364 | 0.6592578 |
| myh11a | 0.4020412 | 0.3927548 | | 0.2058721 | 0.4057952 |
| myhc4 | 0.3068834 | 0.7491182 | | 0.5154421 | 0.7141758 |
| myhz1.2 | 0.263504 | 0.94429 | | 0.7549031 | 0.6062692 |
| myhz2 | 0.3736768 | 0.659486 | | 0.6740068 | 0.7261176 |
| myl9b | 0.3738229 | 0.427194 | | 0.8953727 | 0.860478 |
| myog | 0.4244006 | 0.8191548 | | 0.5811269 | 0.8214485 |
| mysm1 | 0.4007857 | 0.6298841 | | 0.3975764 | 0.5098853 |
| nadsyn1 | 0.3992778 | 0.7111872 | | 0.6689791 | 0.8022517 |
| nat8l | 0.4576493 | 0.7534911 | | 0.5469428 | 0.784759 |
| nbas | 0.4641722 | 0.5824571 | | 0.4632593 | 0.5685061 |
| ncam1a | 0.4177355 | 0.4838668 | | 0.3154295 | 0.6628608 |
| ncam1b | 0.4766463 | 0.9169149 | | 0.9276387 | 0.9837208 |
| ncapd2 | 0.4542443 | 0.6663716 | | 0.7251263 | 0.6871445 |
| ncapd3 | 0.3641178 | 0.7109906 | | 0.6922587 | 0.6175034 |
| ncapg | 0.3517149 | 0.7061477 | | 0.6583191 | 0.6425158 |
| ncapg2 | 0.3995931 | 0.8547548 | | 0.8053048 | 0.7025399 |
| ndrg1b | 0.1680196 | 0.0329462 | | 0.0782766 | 0.1629765 |
| necap1 | 0.4897476 | 0.4530059 | | 0.2570816 | 0.4229481 |
| nefl | 0.3526228 | 0.6726215 | | 0.5657122 | 0.3961493 |
| nefm | 0.364443 | 0.682279 | | 0.7761233 | 0.4691586 |
| neil3 | 0.2609305 | 0.6194572 | | 0.6349553 | 0.4035194 |
| nek2 | 0.3345779 | 0.5987702 | | 0.6631646 | 0.705619 |
| neurod | 0.3072665 | 0.1307269 | | 0.1072846 | 0.2219237 |
| neurod4 | 0.2249867 | 0.390051 | | 0.3281324 | 0.5107975 |
| nfat5 | 0.2977915 | 0.4873468 | | 0.4587251 | 0.8332425 |
| nfe2l3 | 0.4946757 | 0.5908331 | | 0.4121979 | 0.581492 |
| nkapl | 0.4840173 | 0.5378719 | | 0.7296811 | 0.6288321 |
| nkrf | 0.4109953 | 0.7547651 | | 0.6550055 | 0.6115636 |
| nkx2.1a | 0.1154893 | 0.1195465 | | 0.2371071 | 0.511562 |
| nkx2.1b | 0.2721272 | 0.3294896 | | 0.3856803 | 0.4040841 |
| nkx2.3 | 0.4337935 | 0.603609 | | 0.3973807 | 0.6494083 |
| nkx2.5 | 0.1799516 | 0.223477 | | 0.4778173 | 0.3224239 |
| nkx3.3 | 0.3262584 | 0.440422 | | 0.2257655 | 0.3920242 |
| nkx6.1 | 0.4870668 | 0.6015019 | | 0.4795049 | 0.6031567 |
| nme7 | 0.473788 | 0.5985341 | | 0.6507839 | 0.5861907 |
| nmt2 | 0.435863 | 0.5966491 | | 0.4219807 | 0.5460322 |
| nog2 | 0.3624999 | 0.9059156 | | 0.6469999 | 0.5802315 |
| nol8 | 0.248433 | 0.8706233 | | 0.6245226 | 0.8013548 |
| nono | 0.4983604 | 0.7510085 | | 0.7470382 | 0.7624099 |
| nptna | 0.4431235 | 0.5232094 | | 0.4080982 | 0.5045825 |
| nptxrb | 0.3924223 | 0.4872603 | | 0.5187333 | 0.7168212 |
| npvf | 0.125856 | 0.1758649 | | 0.2217815 | 0.5147906 |
| nr2e1 | 0.2563111 | 0.4496237 | | 0.6279654 | 0.5193731 |
| nr2e3 | 0.1849934 | 0.089335 | | 0.0592642 | 0.1895253 |
| nr2f1b | 0.494608 | 0.4724095 | | 0.544673 | 0.5811472 |
| nr2f2 | 0.4611308 | 0.4034353 | | 0.2964175 | 0.4794951 |
| nsfa | 0.3776762 | 0.4069945 | | 0.3031251 | 0.4047935 |
| nucks1b | 0.4169404 | 0.5239882 | | 0.4450864 | 0.494608 |
| nuf2 | 0.3738546 | 0.5315994 | | 0.3991362 | 0.6340488 |
| numbl | 0.3215208 | 0.9897488 | | 0.7023914 | 0.6316329 |
| nup155 | 0.4217139 | 0.8805577 | | 0.8350721 | 0.836725 |
| nusap1 | 0.4260821 | 0.5852224 | | 0.5173017 | 0.639555 |
| obfc2aa | 0.3774428 | 0.4823332 | | 0.3774921 | 0.4750913 |
| ogt.1 | 0.4871277 | 0.5286485 | | 0.4963748 | 0.7497548 |
| olig2 | 0.2489198 | 0.6424994 | | 0.2894319 | 0.4497447 |
| onecut1 | 0.4039866 | 0.5016748 | | 0.5054265 | 0.7301653 |
| opn1lw2 | 0.3850128 | 0.2603255 | | 0.6205243 | 0.5167328 |
| orc1 | 0.4813824 | 0.8741875 | | 0.96508 | 0.8949772 |
| orc5 | 0.4912142 | 0.9925438 | | 0.9015467 | 0.8945371 |
| osr1 | 0.4437344 | 0.4495403 | | 0.307283 | 0.4885507 |
| otud5b | 0.4296566 | 0.72146 | | 0.6712263 | 0.6753199 |
| otx1b | 0.4398398 | 0.6032397 | | 0.6395289 | 0.7499824 |
| otx2 | 0.3029126 | 0.2175065 | | 0.2735204 | 0.3616466 |
| otx5 | 0.246517 | 0.0900228 | | 0.0941431 | 0.291987 |
| oxsr1a | 0.4513566 | 0.541055 | | 0.4607326 | 0.5905601 |
| p2rx3a | 0.0862015 | 0.1605753 | | 0 | 0 |
| pacsin1a | 0.2210002 | 0.1961068 | | 0.1384701 | 0.3546953 |
| pacsin1b | 0.4688096 | 0.4987249 | | 0.4594866 | 0.5039191 |
| papd4 | 0.4096681 | 0.6519164 | | 0.7025333 | 0.5819632 |
| papolb | 0.4785426 | 0.9454938 | | 0.6151489 | 0.8196936 |
| papss1 | 0.4674515 | 0.5749604 | | 0.490932 | 0.5393201 |
| parp1 | 0.408504 | 0.7658477 | | 0.6175973 | 0.7014927 |
| parp2 | 0.484279 | 0.6855981 | | 0.6707952 | 0.6270924 |
| parpbp | 0.3809386 | 0.7331855 | | 0.7236964 | 0.8431371 |
| pax6a | 0.2723088 | 0.5252356 | | 0.5397428 | 0.5583143 |
| pax6b | 0.1683901 | 0.3659531 | | 0.4153517 | 0.4556939 |
| paxip1 | 0.4286375 | 0.6077314 | | 0.6114321 | 0.579304 |
| pbrm1l | 0.462984 | 0.6422088 | | 0.5634353 | 0.6760053 |
| pcdh18a | 0.4405109 | 0.4471218 | | 0.3944606 | 0.5932083 |
| pcdh1g22 | 0.3554656 | 0.2282924 | | 0.0990729 | 0.0998264 |
| pcdh1g26 | 0.1903672 | 0.0940674 | | 0.0736086 | 0.064361 |
| pcdh1gc6 | 0.3169912 | 0.5543614 | | 0.1233871 | 0.2951342 |
| pcdh2aa1 | 0.2180232 | 0.069834 | | 0.0608108 | 0 |
| pcdh2ab7 | 0.2384535 | 0.3212249 | | 0.0626178 | 0.2330813 |
| pcdh8 | 0.1030545 | 0.0159943 | | 0 | 0.0792477 |
| pcm1 | 0.4226844 | 0.7089953 | | 0.5581218 | 0.643123 |
| pcmtl | 0.3792097 | 0.9223183 | | 0.7144061 | 0.8212953 |
| pcsk5b | 0.4864866 | 0.6804418 | | 0.7303314 | 0.6877344 |
| pcyt1ab | 0.4529932 | 0.5672714 | | 0.6370313 | 0.6883175 |
| pdcd4b | 0.3598241 | 0.1711208 | | 0.4028113 | 0.8078963 |
| pdgfab | 0.3984124 | 0.8121573 | | 0.8569361 | 0.9565255 |
| pdia2 | 0.1879632 | 0 | | 0 | 0.0481266 |
| pdia4 | 0.3725996 | 0.2331779 | | 0.2217633 | 0.2965296 |
| pds5a | 0.4215359 | 0.7491888 | | 0.6170478 | 0.6468941 |
| pdzd3b | 0.0738989 | 0.4588635 | | 0.065418 | 0 |
| pdzk1 | 0.2040882 | 0.802572 | | 0.5905776 | 0.3344058 |
| pex1 | 0.3645937 | 0.6631946 | | 0.950529 | 0.7426965 |
| pfn2l | 0.4816762 | 0.6858007 | | 0.4402538 | 0.5825848 |
| phax | 0.3964176 | 0.6444028 | | 0.5761254 | 0.6054209 |
| phc1 | 0.4798053 | 0.5522397 | | 0.5614105 | 0.5240295 |
| phf16 | 0.4937744 | 0.7930861 | | 0.7253781 | 0.8624182 |
| phf17 | 0.4572713 | 0.4957864 | | 0.5215276 | 0.6141422 |
| phf2 | 0.406116 | 0.4912313 | | 0.2646962 | 0.4894771 |
| phf20b | 0.4597178 | 0.7044503 | | 0.538951 | 0.7158657 |
| phf23a | 0.4667245 | 0.8558683 | | 0.715711 | 0.6046126 |
| phf23b | 0.4192501 | 0.5205758 | | 0.4411785 | 0.446675 |
| phf6 | 0.466861 | 0.9336935 | | 0.8005772 | 0.85055 |
| phf8 | 0.4267429 | 0.4248505 | | 0.397674 | 0.5088161 |
| phlda3 | 0.3731046 | 0.8622472 | | 0.9293966 | 0.722584 |
| phox2bb | 0.4127347 | 0.4193602 | | 0.3511131 | 0.2535057 |
| pias4a | 0.4037213 | 0.8213905 | | 0.6322298 | 0.7562095 |
| picalm | 0.4982321 | 0.7206694 | | 0.5584708 | 0.6086824 |
| pif1 | 0.3697779 | 0.5781894 | | 0.6489421 | 0.8141187 |
| pisd | 0.3868892 | 0.5936609 | | 0.472293 | 0.6673259 |
| piwil2 | 0.2511956 | 0.1403462 | | 0.0894759 | 0.1288068 |
| pknox1.1 | 0.4018546 | 0.4025927 | | 0.6083274 | 0.5100932 |
| plac8.1 | 0.1715092 | 0.3434834 | | 0.1326911 | 0.0776858 |
| plagl2 | 0.3785042 | 0.4099705 | | 0.3868083 | 0.5284118 |
| plagx | 0.4678376 | 0.6153961 | | 0.4718492 | 0.5758788 |
| plk1 | 0.4512406 | 0.7007119 | | 0.7504984 | 0.7289756 |
| plk4 | 0.3371221 | 0.5300617 | | 0.4963727 | 0.55246 |
| plod2 | 0.372118 | 0.8392718 | | 0.4745932 | 0.6518403 |
| plrg1 | 0.4378443 | 0.8910494 | | 0.9056017 | 0.8885073 |
| pls1 | 0.2764766 | 0.8269902 | | 0.7827724 | 0.3350233 |
| pltp | 0.4214015 | 0.453532 | | 0.5725459 | 0.5466702 |
| pmelb | 0.3611368 | 0.5099143 | | 0.7005776 | 0.7012829 |
| pola2 | 0.4853108 | 0.644743 | | 0.7770064 | 0.5702771 |
| pold3 | 0.36961 | 0.4406089 | | 0.5006525 | 0.5107883 |
| pole | 0.4451055 | 0.5971156 | | 0.6221351 | 0.6020817 |
| pole3 | 0.4843788 | 0.6195548 | | 0.5817738 | 0.6074968 |
| pomca | 0.2500056 | 0.1074918 | | 0.1016447 | 0.2163073 |
| pot1 | 0.3440214 | 0.614066 | | 0.7510526 | 0.5288208 |
| pou2f1b | 0.4521764 | 0.3823932 | | 0.276587 | 0.4437245 |
| pou3f1 | 0.4250741 | 0.6227025 | | 0.4072796 | 0.4973072 |
| pou3f3a | 0.3518798 | 0.6698186 | | 0.7036312 | 0.6933738 |
| pou3f3b | 0.4243315 | 0.4348931 | | 0.4023963 | 0.5095794 |
| pou4f1 | 0.4800601 | 0.2704405 | | 0.32798 | 0.2597186 |
| pou4f2 | 0.3523902 | 0.1259897 | | 0.0943835 | 0.2076524 |
| pou6f1 | 0.4672926 | 0.3735546 | | 0.3644977 | 0.5447472 |
| ppa1 | 0.4919109 | 0.9195836 | | 0.6729965 | 0.7141484 |
| ppig | 0.4101408 | 0.4329798 | | 0.522961 | 0.5676287 |
| ppil4 | 0.442092 | 0.8234041 | | 0.7210773 | 0.7418292 |
| ppm1g | 0.4925727 | 0.6642298 | | 0.6661151 | 0.6210361 |
| ppp1r14ba | 0.4524916 | 0.3696965 | | 0.2803579 | 0.3661427 |
| ppp2ca | 0.4558818 | 0.3147805 | | 0.3100187 | 0.4669339 |
| ppp3cca | 0.3518569 | 0.566486 | | 0.492756 | 0.5500996 |
| ppp4r2a | 0.4194176 | 0.5535978 | | 0.4803186 | 0.6465939 |
| ppt2 | 0.4817851 | 0.5315264 | | 0.4516717 | 0.5766729 |
| ppwd1 | 0.4882398 | 0.8716954 | | 0.7127858 | 0.6648165 |
| prdm8b | 0.3298359 | 0.4095535 | | 0.1371874 | 0.3740279 |
| prep | 0.4411548 | 0.7326357 | | 0.5858504 | 0.5652779 |
| prkcbp1l | 0.4320884 | 0.4657281 | | 0.4523961 | 0.4787248 |
| prkrira | 0.4341169 | 0.6448344 | | 0.5452363 | 0.5405809 |
| prkrirb | 0.4509051 | 0.5883538 | | 0.4209708 | 0.2898129 |
| prl | 0.3497013 | 0.2869649 | | 0.1654167 | 0.2555769 |
| prmt10 | 0.4191322 | 0.6765443 | | 0.4919462 | 0.5757543 |
| prmt2 | 0.30921 | 0.5259238 | | 0.5113615 | 0.5642556 |
| prmt8b | 0.1894438 | 0.1176737 | | 0.0830801 | 0.2902203 |
| prom1b | 0.2823261 | 0.2165018 | | 0.1183478 | 0.1021989 |
| prosc | 0.4290204 | 0.8717047 | | 0.7504187 | 0.5654529 |
| prox1a | 0.3952972 | 0.3979624 | | 0.4655438 | 0.5092948 |
| prpf3 | 0.4782778 | 0.6718004 | | 0.6271093 | 0.710875 |
| prpf38b | 0.4587213 | 0.9845874 | | 0.8003362 | 0.8177094 |
| prpf4 | 0.4186039 | 0.9654081 | | 0.9137886 | 0.8777842 |
| prpf40a | 0.4112918 | 0.8983197 | | 0.6968447 | 0.7461965 |
| prpf4bb | 0.3666907 | 0.5828476 | | 0.4733532 | 0.5981525 |
| prpf8 | 0.4649907 | 0.7794096 | | 0.6031527 | 0.6911634 |
| pspc1 | 0.4349332 | 0.6539912 | | 0.5121987 | 0.5217649 |
| ptch2 | 0.4778181 | 0.9071542 | | 0.4565069 | 0.7897625 |
| ptf1a | 0.3462944 | 0.9235417 | | 0.4842496 | 0.6872647 |
| ptges3a | 0.4566473 | 0.8534509 | | 0.6344245 | 0.7496753 |
| ptprna | 0.4605369 | 0.6970732 | | 0.9949382 | 0.8454059 |
| puf60a | 0.4730969 | 0.9340421 | | 0.7858136 | 0.8460259 |
| pvalb6 | 0.3816095 | 0.6290684 | | 0.2188067 | 0.4196994 |
| pvrl1a | 0.1723309 | 0.3210089 | | 0.1526692 | 0.1764847 |
| pygo2 | 0.3903722 | 0.5968043 | | 0.417711 | 0.4810245 |
| rab3ab | 0.252811 | 0.3141531 | | 0.1258546 | 0.2209009 |
| rab6b | 0.4466077 | 0.4350862 | | 0.2750779 | 0.2888317 |
| rabl5 | 0.383657 | 0.7435867 | | 0.51046 | 0.579592 |
| rac3a | 0.4074337 | 0.3979603 | | 0.368772 | 0.34217 |
| racgap1 | 0.4483965 | 0.5638093 | | 0.6020393 | 0.6293282 |
| rad18 | 0.1689987 | 0.6782519 | | 0.6057012 | 0.5739728 |
| rangap1 | 0.4261589 | 0.8038834 | | 0.741091 | 0.6024025 |
| rap2ip | 0.4673442 | 0.3659503 | | 0.3030667 | 0.4401763 |
| rbfox1 | 0.4701353 | 0.3534595 | | 0.2918006 | 0.2731814 |
| rbm25 | 0.4729625 | 0.9587795 | | 0.841256 | 0.9375953 |
| rbm39b | 0.4728454 | 0.792886 | | 0.7870551 | 0.8063982 |
| rbm4.2 | 0.4724659 | 0.7627029 | | 0.6661025 | 0.6761644 |
| rbm5 | 0.3544233 | 0.5689954 | | 0.5534601 | 0.6790696 |
| rcc2 | 0.4595977 | 0.5312711 | | 0.4762861 | 0.4749844 |
| rcor1 | 0.4729351 | 0.7678292 | | 0.533395 | 0.608741 |
| rcor2 | 0.3797126 | 0.5598721 | | 0.7351008 | 0.7041852 |
| rcv1 | 0.3019942 | 0.2091458 | | 0.1235166 | 0.2855055 |
| rexo1 | 0.4673217 | 0.4498693 | | 0.4103471 | 0.382077 |
| rfc1 | 0.4530791 | 0.6322747 | | 0.502235 | 0.5837073 |
| rfng | 0.3884914 | 0.3943094 | | 0.5210594 | 0.4275964 |
| rfx1a | 0.3589906 | 0.325734 | | 0.360188 | 0.4907073 |
| rfx3 | 0.401766 | 0.5321152 | | 0.4821996 | 0.7070022 |
| rgs5a | 0.4458985 | 0.224921 | | 0.1176962 | 0.1928368 |
| rho | 0.072661 | 0.1353579 | | 0.0481909 | 0.1301574 |
| ric8a | 0.3474808 | 0.2614952 | | 0.4021724 | 0.5179777 |
| rif1 | 0.4303187 | 0.5357482 | | 0.5139099 | 0.5256348 |
| riok1 | 0.4325123 | 0.9421956 | | 0.9420956 | 0.6607332 |
| rlbp1b | 0.4370166 | 0.5243791 | | 0.607961 | 0.5683329 |
| rltpr | 0.4303613 | 0.5753874 | | 0.2919964 | 0.4371407 |
| rnaseh2b | 0.4860413 | 0.6842984 | | 0.6656432 | 0.5340377 |
| rnasen | 0.3980949 | 0.7606931 | | 0.5847133 | 0.6093037 |
| rnf220a | 0.4690288 | 0.6545213 | | 0.5908028 | 0.556644 |
| rnpc3 | 0.4407388 | 0.5321328 | | 0.574453 | 0.8837589 |
| rnpep | 0.4254749 | 0.6944013 | | 0.6718828 | 0.6060173 |
| ropn1l | 0.2234025 | 0.7632447 | | 0.3263293 | 0.5322669 |
| rorab | 0.4352509 | 0.3148363 | | 0.2158231 | 0.3804436 |
| rorb | 0.1570623 | 0.1950695 | | 0.1386823 | 0.1928436 |
| rpgrip1l | 0.2782588 | 0.1468271 | | 0.1859246 | 0.4138334 |
| rpl22l1 | 0.4902734 | 0.5697966 | | 0.7009276 | 0.3914175 |
| rprd2a | 0.4086022 | 0.7346106 | | 0.4495347 | 0.5625865 |
| rrm1 | 0.4698011 | 0.5628295 | | 0.6896765 | 0.6108211 |
| rrnad1 | 0.2292203 | 0.4553885 | | 0.4074199 | 0.3759158 |
| rrs1 | 0.328298 | 0.9893688 | | 0.7623141 | 0.7788023 |
| rs1 | 0.0560962 | 0.1857987 | | 0.0494591 | 0.1051845 |
| rspo1 | 0.272278 | 0.9341884 | | 0.4964981 | 0.5434171 |
| rufy3 | 0.4404522 | 0.5191535 | | 0.4001437 | 0.5734024 |
| runx2b | 0.188057 | 0.2043459 | | 0.1248986 | 0.24071 |
| ruvbl1 | 0.4473185 | 0.98314 | | 0.747923 | 0.7076286 |
| rx1 | 0.0890914 | 0.4543319 | | 0.367873 | 0.5056083 |
| rx2 | 0.1617192 | 0.3730691 | | 0.4267654 | 0.2361379 |
| rx3 | 0.1729266 | 0.1933044 | | 0.18301 | 0.4245053 |
| rxrba | 0.4277503 | 0.8292431 | | 0.4723017 | 0.7478869 |
| rybpa | 0.4297178 | 0.433366 | | 0.3714427 | 0.4589813 |
| safb | 0.4332472 | 0.6928725 | | 0.7194407 | 0.63154 |
| saga | 0.2060698 | 0.335814 | | 0.4355763 | 0.3697785 |
| sall1a | 0.4481672 | 0.6483361 | | 0.3805219 | 0.5817295 |
| samd7 | 0.2720503 | 0.1126122 | | 0.0482139 | 0.2043262 |
| sarm1 | 0.4520874 | 0.4071768 | | 0.2433168 | 0.5808554 |
| sart1 | 0.4431856 | 0.6785926 | | 0.5705097 | 0.6638851 |
| sart3 | 0.4618819 | 0.7760907 | | 0.6820766 | 0.6293772 |
| sass6 | 0.3836822 | 0.7554113 | | 0.5950583 | 0.6519329 |
| satb1a | 0.3492053 | 0.216803 | | 0.1692233 | 0.2765587 |
| scg2b | 0.4877505 | 0.3458743 | | 0.3161746 | 0.4380266 |
| scg3 | 0.3606246 | 0.4012886 | | 0.2636364 | 0.3667346 |
| scmh1 | 0.2833525 | 0.4870571 | | 0.1664811 | 0.2248102 |
| scrt1b | 0.3683569 | 0.3836529 | | 0.4317125 | 0.59632 |
| scrt2 | 0.4568561 | 0.7175061 | | 0.4925479 | 0.7001278 |
| sema3bl | 0.369862 | 0.4264221 | | 0.2821573 | 0.4876133 |
| sema3d | 0.3859328 | 0.2139256 | | 0.2451427 | 0.2515162 |
| sema6d | 0.397795 | 0.487572 | | 0.5266237 | 0.8367569 |
| senp3a | 0.4681452 | 0.6631702 | | 0.5651808 | 0.6115453 |
| sephs1 | 0.4375117 | 0.7196012 | | 0.7526108 | 0.7457247 |
| sept4a | 0.1541641 | 0.2533821 | | 0.2249985 | 0.2693413 |
| sept5a | 0.4763437 | 0.754222 | | 0.6327041 | 0.6445473 |
| sestd1 | 0.3144133 | 0.8061055 | | 0.7147043 | 0.6364172 |
| setb | 0.4916414 | 0.8885883 | | 0.7213179 | 0.8318714 |
| setdb1a | 0.4064937 | 0.6180414 | | 0.5007061 | 0.5499682 |
| setdb1b | 0.4957756 | 0.6364954 | | 0.6109377 | 0.7174643 |
| sf3a1 | 0.4206943 | 0.9096895 | | 0.8582225 | 0.8389314 |
| sgk3 | 0.4609301 | 0.5630785 | | 0.3041495 | 0.3505961 |
| sgsm3 | 0.4838312 | 0.6375758 | | 0.4519439 | 0.6483774 |
| sh2b1 | 0.4876129 | 0.531582 | | 0.4744494 | 0.7025332 |
| sh3bgrl2 | 0.2087516 | 0.1978247 | | 0.2827024 | 0.315182 |
| shha | 0.3509454 | 0.6441689 | | 0.5153049 | 0.6568737 |
| shhb | 0.2900327 | 0.4389431 | | 0.2732566 | 0.3713758 |
| shisa3 | 0.3889563 | 0.5263723 | | 0.4366787 | 0.5000263 |
| si:busm1-142b24.1 | 0.3326558 | 0.3852212 | | 0.3930503 | 0.5445253 |
| si:ch1073-463l14.2 | 0.4039155 | 0.3601824 | | 0.2201101 | 0.3929993 |
| si:ch211-106n13.3 | 0.1211129 | 0.1503684 | | 0.0539558 | 0.2484522 |
| si:ch211-13c6.2 | 0.4505966 | 0.6425451 | | 0.4380156 | 0.5701169 |
| si:ch211-154o6.2 | 0.4977386 | 0.605533 | | 0.6686626 | 0.6512919 |
| si:ch211-175l6.9 | 0.4471556 | 0.7923458 | | 0.6989582 | 0.7977465 |
| si:ch211-198d18.2 | 0.4467358 | 0.6487297 | | 0.3626741 | 0.4582771 |
| si:ch211-216l23.2 | 0.4638748 | 0.5852045 | | 0.5687706 | 0.713751 |
| si:ch211-220f13.1 | 0.4914764 | 0.2930342 | | 0.257162 | 0.3322967 |
| si:ch211-221n23.1 | 0.4650643 | 0.4471163 | | 0.489405 | 0.7705018 |
| si:ch211-236k19.4 | 0.2879163 | 0.3849692 | | 0.1873729 | 0.4429155 |
| si:ch211-244o22.2 | 0.3896636 | 0.5444792 | | 0.5381067 | 0.5618032 |
| si:ch211-244p18.3 | 0.3642805 | 0.7005525 | | 0.3918489 | 0.5752982 |
| si:ch211-247j6.1 | 0.4976318 | 0.5221819 | | 0.4467268 | 0.7224648 |
| si:ch211-251j10.5 | 0.4024356 | 0.8017363 | | 0.6143269 | 0.7023654 |
| si:ch211-255a21.1 | 0.4522593 | 0.4371421 | | 0.450846 | 0.5693825 |
| si:ch211-262h13.3 | 0.4679849 | 0.7235613 | | 0.6390943 | 0.5570785 |
| si:ch211-67n3.1 | 0.4294643 | 0.6238869 | | 0.359121 | 0.6956935 |
| si:ch211-69g19.2 | 0.4818638 | 0.5920133 | | 0.705003 | 0.7000437 |
| si:ch211-80h18.1 | 0.3538597 | 0.4779208 | | 0.3308698 | 0.6011022 |
| si:dkey-105e17.1 | 0.2798804 | 0.3128314 | | 0.1979654 | 0.5729445 |
| si:dkey-119f1.1 | 0.3196116 | 0.5889369 | | 0.5014894 | 0.5254922 |
| si:dkey-147f20.5 | 0.4976845 | 0.7188427 | | 0.6094228 | 0.7125592 |
| si:dkey-15f17.9 | 0.1838066 | 0.3993573 | | 0.1146608 | 0.2828099 |
| si:dkey-174m14.3 | 0.2576522 | 0.2908247 | | 0.187555 | 0.2401546 |
| si:dkey-20d21.12 | 0.4026787 | 0.6252084 | | 0.5414132 | 0.7584801 |
| si:dkey-22i16.3 | 0.1311148 | 0.1503328 | | 0.0355227 | 0.0412467 |
| si:dkey-22l11.2 | 0.4534997 | 0.5155464 | | 0.4715575 | 0.5434597 |
| si:dkey-231l1.6 | 0.3365584 | 0.6584614 | | 0.5175615 | 0.6170627 |
| si:dkey-235k4.1 | 0.2490908 | 0.3867329 | | 0.1463941 | 0.467048 |
| si:dkey-245p14.4 | 0.2765788 | 0.3245552 | | 0.6445464 | 0.1881266 |
| si:dkey-252h13.5 | 0.1646802 | 0.1024277 | | 0 | 0 |
| si:dkey-264p5.1 | 0.1229349 | 0.2673046 | | 0.3222655 | 0.1254249 |
| si:dkey-33c12.4 | 0.3756312 | 0.7299964 | | 0.575648 | 0.6955917 |
| si:dkey-52k20.7 | 0.0944638 | 0.1466912 | | 0.0761647 | 0.0296562 |
| si:dkey-57a22.11 | 0.3816585 | 0.450182 | | 0.3481072 | 0.4695995 |
| si:dkey-67c22.2 | 0.4256378 | 0.5816061 | | 0.4528132 | 0.7456211 |
| si:dkey-6n6.1 | 0.2338191 | 0.4476143 | | 0.6743067 | 0.6587952 |
| si:dkey-76p14.4 | 0.409691 | 0.3463726 | | 0.2135737 | 0.4798007 |
| si:dkey-98f17.5 | 0.3953681 | 0.5333607 | | 0.481807 | 0.7673423 |
| si:dkeyp-117h8.4 | 0.315025 | 0.4138434 | | 0.4299115 | 0.4491964 |
| si:dkeyp-35b8.5 | 0.4314699 | 0.3255003 | | 0.5978226 | 0.5418137 |
| si:dkeyp-86e4.1 | 0.4341466 | 0.3030498 | | 0.2601436 | 0.3261811 |
| si:rp71-1g18.1 | 0.4987731 | 0.5258965 | | 0.4621045 | 0.4935297 |
| sin3aa | 0.4745628 | 0.5498248 | | 0.3941047 | 0.6150889 |
| sin3b | 0.4682477 | 0.6088303 | | 0.5274477 | 0.5643052 |
| six1a | 0.3438887 | 0.5716636 | | 0.6083382 | 0.7993919 |
| six1b | 0.465187 | 0.752827 | | 0.8294613 | 0.7655381 |
| six3a | 0.2746824 | 0.3468827 | | 0.3053761 | 0.43394 |
| six3b | 0.2551786 | 0.6059572 | | 0.4090909 | 0.4484173 |
| six4.3 | 0.4149535 | 0.4665086 | | 0.1693748 | 0.3563163 |
| six6b | 0.1559559 | 0.2376978 | | 0.2299832 | 0.2902922 |
| six7 | 0.2097317 | 0.0532627 | | 0.0169855 | 0.1173345 |
| ska3 | 0.371344 | 0.4869863 | | 0.6125648 | 0.4753662 |
| skiv2l2 | 0.4222922 | 0.9944929 | | 0.9823708 | 0.8899662 |
| skp2 | 0.3656129 | 0.7049711 | | 0.6787499 | 0.6199693 |
| slc12a10.3 | 0.276198 | 0.249393 | | 0.6264134 | 0.1545251 |
| slc15a1b | 0.0937588 | 0.1164175 | | 0.1666788 | 0.1922125 |
| slc17a6a | 0.3205658 | 0.4326492 | | 0.2229873 | 0.3714469 |
| slc1a3b | 0.4540582 | 0.4207683 | | 0.2243546 | 0.4200267 |
| slc1a5 | 0.4073933 | 0.5211103 | | 0.338918 | 0.5005199 |
| slc25a14 | 0.4695414 | 0.5796599 | | 0.3659135 | 0.5198615 |
| slc25a46 | 0.4709833 | 0.6295456 | | 0.6146221 | 0.4958094 |
| slc26a1 | 0.1886393 | 0.5465284 | | 0.2571422 | 0.5285469 |
| slc26a6l | 0.0858954 | 0.266627 | | 0 | 0.1761286 |
| slc39a10 | 0.4801301 | 0.4828581 | | 0.4147447 | 0.5859583 |
| slc5a11 | 0.0793776 | 0.1971269 | | 0.4935443 | 0.0813493 |
| slc5a2 | 0.1720047 | 0.373766 | | 0.9165466 | 0.7491471 |
| slc5a9 | 0.0584408 | 0.907105 | | 0.657063 | 0.359287 |
| slc6a5 | 0.3016554 | 0.3745437 | | 0.3801881 | 0.5154692 |
| slit2 | 0.3632371 | 0.580798 | | 0.5608596 | 0.6831183 |
| slmapa | 0.494158 | 0.6546534 | | 0.6568642 | 0.697143 |
| slmapb | 0.3333892 | 0.4485017 | | 0.2659791 | 0.3642467 |
| sltm | 0.4755144 | 0.6990717 | | 0.6159026 | 0.5984516 |
| smad2 | 0.4543747 | 0.7423293 | | 0.742904 | 0.7723045 |
| smarca5 | 0.4365482 | 0.7580462 | | 0.7459517 | 0.7403415 |
| smarcad1 | 0.3955149 | 0.6306806 | | 0.5151982 | 0.5758233 |
| smarcc1b | 0.3576064 | 0.6576869 | | 0.5768576 | 0.6760758 |
| smarcd1 | 0.4679575 | 0.7119641 | | 0.6335427 | 0.8586343 |
| smarcd3b | 0.4430032 | 0.5501955 | | 0.6358794 | 0.5828377 |
| smc1al | 0.3932665 | 0.5101339 | | 0.4799731 | 0.5720808 |
| smc2 | 0.3602652 | 0.56025 | | 0.5075825 | 0.5668077 |
| smc3 | 0.441976 | 0.5270099 | | 0.5211742 | 0.5665993 |
| smc4 | 0.4230949 | 0.6210959 | | 0.7833559 | 0.6651243 |
| smc5 | 0.4305103 | 0.5683459 | | 0.754557 | 0.6371034 |
| smg7 | 0.4985142 | 0.780638 | | 0.5453777 | 0.7430189 |
| smpd4 | 0.4110803 | 0.9716039 | | 0.6525185 | 0.6196283 |
| snap25b | 0.4964555 | 0.3350042 | | 0.3071125 | 0.4640475 |
| snapc4 | 0.342921 | 0.4320027 | | 0.4677155 | 0.7657233 |
| sncb | 0.4805421 | 0.2912636 | | 0.2894448 | 0.3511055 |
| socs6b | 0.4742139 | 0.5985325 | | 0.4567083 | 0.618788 |
| sox11b | 0.3940918 | 0.6448554 | | 0.4734191 | 0.526353 |
| sox1a | 0.3809941 | 0.301368 | | 0.4118177 | 0.4918339 |
| sox1b | 0.4538474 | 0.3495771 | | 0.5546822 | 0.5530203 |
| sox2 | 0.3238411 | 0.4344813 | | 0.420553 | 0.4479558 |
| sox21b | 0.3959807 | 0.5464602 | | 0.565747 | 0.7780351 |
| sox3 | 0.4330815 | 0.9411803 | | 0.9165738 | 0.8248247 |
| sox9a | 0.3681783 | 0.4620798 | | 0.4135895 | 0.5567473 |
| sox9b | 0.2998828 | 0.5352078 | | 0.4698136 | 0.630029 |
| sp7 | 0.1480653 | 0.2758408 | | 0.1743525 | 0.454517 |
| sparcl1 | 0.372958 | 0.4341919 | | 0.3719806 | 0.4377371 |
| srd5a1 | 0.1034093 | 0.1926255 | | 0.1831973 | 0.4235925 |
| srpk1b | 0.3726954 | 0.7825379 | | 0.7718491 | 0.7907664 |
| srrm2 | 0.4127438 | 0.7647586 | | 0.6358168 | 0.8293043 |
| srrt | 0.4815075 | 0.9959873 | | 0.8017947 | 0.7923744 |
| srsf2b | 0.4277187 | 0.7525471 | | 0.6897667 | 0.7575294 |
| ssrp1a | 0.433471 | 0.7045752 | | 0.6215181 | 0.5655892 |
| sst6 | 0.1672769 | 0.1733164 | | 0.239295 | 0.1132135 |
| star | 0.3785322 | 0.2502485 | | 0.441332 | 0.1623469 |
| stil | 0.4258014 | 0.5638889 | | 0.379602 | 0.5605799 |
| stip1 | 0.4471215 | 0.6920734 | | 0.678285 | 0.8524977 |
| stk36 | 0.329346 | 0.6474203 | | 0.4770053 | 0.4223017 |
| stm | 0.3135831 | 0.2452552 | | 0.1035791 | 0.2052298 |
| stxbp1a | 0.4808454 | 0.4967089 | | 0.466975 | 0.5131563 |
| stxbp1b | 0.2515444 | 0.1581244 | | 0.1005353 | 0.2610084 |
| suds3 | 0.4374662 | 0.6728938 | | 0.5766942 | 0.6429189 |
| sulf1 | 0.4905692 | 0.817695 | | 0.5917085 | 0.7109493 |
| supt6h | 0.476697 | 0.728221 | | 0.5534333 | 0.6185715 |
| surf6 | 0.3198661 | 0.9307773 | | 0.653085 | 0.8320394 |
| suv39h1b | 0.441299 | 0.5452237 | | 0.555221 | 0.508217 |
| suv420h1 | 0.4888645 | 0.3854876 | | 0.445019 | 0.6032936 |
| suz12b | 0.4420521 | 0.6782614 | | 0.4706802 | 0.5166261 |
| syn2a | 0.4094778 | 0.4789068 | | 0.3949731 | 0.4073375 |
| syncrip | 0.4750135 | 0.6217077 | | 0.5220331 | 0.6184721 |
| syncripl | 0.4515009 | 0.3130812 | | 0.273405 | 0.372565 |
| sypb | 0.43147 | 0.3837726 | | 0.2731947 | 0.4594637 |
| syt5b | 0.1476166 | 0.1374815 | | 0.1177969 | 0.2116943 |
| tacc3 | 0.4731586 | 0.6609127 | | 0.6567962 | 0.6270543 |
| taf1 | 0.4879063 | 0.5250136 | | 0.4253854 | 0.5667697 |
| taf12 | 0.4489395 | 0.5871214 | | 0.4238699 | 0.6435322 |
| taf2 | 0.4233275 | 0.5891899 | | 0.3891531 | 0.6261862 |
| tbc1d20 | 0.2342516 | 0.270167 | | 0.6199015 | 0.37654 |
| tbr1b | 0.4828477 | 0.3214135 | | 0.5648205 | 0.536986 |
| tbx1 | 0.3333087 | 0.3019737 | | 0.2280412 | 0.2935008 |
| tbx15 | 0.386972 | 0.388078 | | 0.4369406 | 0.3967517 |
| tbx18 | 0.3276445 | 0.6407747 | | 0.407349 | 0.6379514 |
| tbx21 | 0.073727 | 0.9154846 | | 0.7201113 | 0.7555132 |
| tbx2a | 0.355179 | 0.3527904 | | 0.2529919 | 0.3257911 |
| tbx2b | 0.3798907 | 0.2723098 | | 0.3311291 | 0.4909772 |
| tbx4 | 0.2664118 | 0.3101361 | | 0.1773805 | 0.4094825 |
| tcea1 | 0.4774907 | 0.6623645 | | 0.51401 | 0.6165818 |
| tcf12 | 0.448392 | 0.6406882 | | 0.4856933 | 0.6255252 |
| tcf21 | 0.1331564 | 0.5112797 | | 0.2869917 | 0.3462724 |
| tcf7l1b | 0.387207 | 0.4263303 | | 0.5219512 | 0.447641 |
| tchp | 0.4686942 | 0.4796162 | | 0.6622163 | 0.5824379 |
| tdrd1 | 0.2639568 | 0.2457877 | | 0.3057527 | 0.2978192 |
| terf2ip | 0.4753277 | 0.805212 | | 0.572703 | 0.5651384 |
| tert | 0.4209571 | 0.5059763 | | 0.4784854 | 0.5235469 |
| tfdp1a | 0.4978333 | 0.7081122 | | 0.698666 | 0.7676034 |
| tfip11 | 0.4956294 | 0.8192077 | | 0.8689872 | 0.8037665 |
| tfr1b | 0.354355 | 0.5461737 | | 0.2138383 | 0.3633358 |
| tgfbrap1 | 0.4271953 | 0.8186981 | | 0.8425327 | 0.7902476 |
| tgif1 | 0.4277355 | 0.6548565 | | 0.6950329 | 0.7366974 |
| thoc2 | 0.4201439 | 0.6964587 | | 0.5108201 | 0.7055439 |
| thoc3 | 0.4707125 | 0.8390576 | | 0.6870695 | 0.6236616 |
| tial1 | 0.4703385 | 0.6363444 | | 0.5374152 | 0.5962889 |
| ticrr | 0.420353 | 0.5940437 | | 0.4108837 | 0.5551814 |
| tinf2 | 0.4521177 | 0.6266806 | | 0.6381353 | 0.7757237 |
| tlk2 | 0.4955921 | 0.7270706 | | 0.6973668 | 0.7821649 |
| tmem161a | 0.292047 | 0.8696497 | | 0.6749782 | 0.842459 |
| tmem161b | 0.378867 | 0.5708476 | | 0.3654308 | 0.5139894 |
| tmem183a | 0.3930388 | 0.6570093 | | 0.3843937 | 0.4645982 |
| tmpob | 0.4045328 | 0.583612 | | 0.5741121 | 0.5971715 |
| tmx3 | 0.1479737 | 0 | | 0.0217981 | 0.0504818 |
| tnfaip8 | 0.4462873 | 0.3676264 | | 0.3515673 | 0.3872776 |
| tnfsf10 | 0.4754467 | 0.5080354 | | 0.3037209 | 0.311412 |
| tnni2a.1 | 0.0873807 | 0.1628829 | | 0 | 0 |
| tnni2b.1 | 0.3916558 | 0.9645531 | | 0.8197614 | 0.7220049 |
| tnpo2 | 0.4004309 | 0.7484561 | | 0.6627209 | 0.8156644 |
| tnr | 0.2939849 | 0.2867778 | | 0.2246666 | 0.3662228 |
| top1l | 0.3602599 | 0.4062143 | | 0.3603271 | 0.4772277 |
| top2a | 0.381036 | 0.5363525 | | 0.5332507 | 0.5571962 |
| top2b | 0.3809496 | 0.5451097 | | 0.4318319 | 0.546056 |
| topbp1 | 0.3696187 | 0.7543696 | | 0.6163101 | 0.6028514 |
| tox | 0.4592315 | 0.4601921 | | 0.5007994 | 0.619746 |
| tp53 | 0.4391119 | 0.747606 | | 0.617356 | 0.669149 |
| tp53rk | 0.4859658 | 0.9132565 | | 0.8286728 | 0.8132063 |
| tpm1 | 0.4317629 | 0.7954658 | | 0.3801749 | 0.5900769 |
| tprb | 0.4014469 | 0.6688185 | | 0.4356838 | 0.5902498 |
| tra2b | 0.4284489 | 0.6190353 | | 0.7839522 | 0.700572 |
| traip | 0.3709444 | 0.4651406 | | 0.4775248 | 0.3204251 |
| trh | 0.2645386 | 0.3135812 | | 0.2130201 | 0.2216489 |
| trim33 | 0.4973112 | 0.685146 | | 0.4884136 | 0.6532647 |
| trim47 | 0.4719552 | 0.6894746 | | 0.6306071 | 0.5518022 |
| trim9 | 0.3928933 | 0.5735072 | | 0.3781839 | 0.5771479 |
| trmt11 | 0.393152 | 0.9262966 | | 0.5486493 | 0.7556046 |
| trub1 | 0.3173896 | 0.7419767 | | 0.5406886 | 0.5284984 |
| try | 0.2661108 | 0.5070246 | | 0.3093641 | 0.2170692 |
| tshb | 0.0998162 | 0.0620196 | | 0.0868824 | 0.1017286 |
| ttk | 0.4990661 | 0.6123569 | | 0.8177127 | 0.7118959 |
| tubgcp2 | 0.4947628 | 0.9172053 | | 0.8618818 | 0.6375889 |
| tubgcp3 | 0.446926 | 0.6564458 | | 0.6124758 | 0.6302207 |
| tubgcp5 | 0.4277877 | 0.6509862 | | 0.617073 | 0.5940534 |
| tulp4 | 0.2197508 | 0.2113718 | | 2.076E-05 | 0.3326524 |
| uba2 | 0.493601 | 0.7330161 | | 0.7494528 | 0.6978408 |
| ubn2 | 0.4810073 | 0.6901219 | | 0.5652105 | 0.7549046 |
| ubp1 | 0.4360665 | 0.6180179 | | 0.4707108 | 0.466106 |
| ubr5 | 0.4889279 | 0.6709966 | | 0.6343989 | 0.7517971 |
| ubxn7 | 0.4790429 | 0.8349931 | | 0.8455373 | 0.7126034 |
| uck2a | 0.3565635 | 0.6537052 | | 0.4274237 | 0.5160784 |
| ugt5c3 | 0.317209 | 0.3940394 | | 0.2115811 | 0.4335977 |
| uhrf1 | 0.4003186 | 0.5481674 | | 0.491816 | 0.5370596 |
| unc119b | 0.3743612 | 0.4194461 | | 0.5154511 | 0.5378552 |
| uox | 0.0762694 | 0.047376 | | 0.133723 | 0.1298059 |
| upf3a | 0.370224 | 0.3646361 | | 0.1470107 | 0.4314322 |
| upf3b | 0.466648 | 0.6010333 | | 0.554136 | 0.5700972 |
| upk3l | 0.0941079 | 0 | | 0 | 0.0963366 |
| uri1 | 0.4232668 | 0.5739865 | | 0.6643035 | 0.6905616 |
| use1 | 0.4783264 | 0.6464127 | | 0.6262484 | 0.759987 |
| usp1 | 0.4584723 | 0.6270079 | | 0.6113494 | 0.5273696 |
| usp39 | 0.4573173 | 0.8220315 | | 0.7798577 | 0.8875573 |
| usp49 | 0.3946507 | 0.6898323 | | 0.6038191 | 0.7715052 |
| usp54a | 0.3726838 | 0.5450607 | | 0.3432482 | 0.6932218 |
| vasa | 0.128864 | 0.1723112 | | 0.0881591 | 0.1422727 |
| vasnb | 0.4824621 | 0.2279821 | | 0.2693808 | 0.3655741 |
| vax1 | 0.2436626 | 0.2117848 | | 0.2093232 | 0.5411222 |
| vezf1b | 0.4272623 | 0.5188207 | | 0.378066 | 0.6123893 |
| vgll4 | 0.0365289 | 0.0824596 | | 0.0023143 | 0.0689963 |
| vil1l | 0.1962543 | 0.5787197 | | 0.3711689 | 0.3270119 |
| vmhc | 0.1179502 | 0.0646948 | | 0.0685304 | 0.0675685 |
| vmhcl | 0.1684013 | 0.4305102 | | 0.2139387 | 0.1242232 |
| vrk3 | 0.4670908 | 0.8474647 | | 0.5433573 | 0.7656767 |
| vsx1 | 0.0948385 | 0.1030582 | | 0.0908666 | 0.1132603 |
| vsx2 | 0.1339804 | 0.461227 | | 0.3715954 | 0.4741126 |
| wacb | 0.3394812 | 0.6404632 | | 0.4232338 | 0.5180166 |
| wdhd1 | 0.4904427 | 0.4877128 | | 0.6749486 | 0.569758 |
| wdr33 | 0.4134799 | 0.9124505 | | 0.7691857 | 0.8306116 |
| wdr5 | 0.4876123 | 0.8715812 | | 0.7183358 | 0.7204069 |
| wfdc1 | 0.4121123 | 0.6225234 | | 0.599577 | 0.5297826 |
| whsc1 | 0.3400689 | 0.6417873 | | 0.6134083 | 0.6031145 |
| wu:fi41d10 | 0.2725093 | 0.8843134 | | 0.9298272 | 0.6530509 |
| wu:fk11d03 | 0.4936761 | 0.5080922 | | 0.5578587 | 0.627509 |
| xpc | 0.3628411 | 0.6965906 | | 0.6185284 | 0.6877732 |
| xrcc6 | 0.3995894 | 0.5686396 | | 0.586873 | 0.4420301 |
| yaf2 | 0.4582059 | 0.9074974 | | 0.5621119 | 0.6954753 |
| yth2 | 0.3252411 | 0.8352146 | | 0.7039556 | 0.7543628 |
| ythdf1 | 0.4979426 | 0.4840909 | | 0.5283162 | 0.6770002 |
| ythdf3 | 0.4336517 | 0.7781478 | | 0.5734025 | 0.6242847 |
| zbed4 | 0.3903205 | 0.4175924 | | 0.3587286 | 0.4599298 |
| zbtb49 | 0.443454 | 0.8111196 | | 0.9909507 | 0.7972156 |
| zc2hc1a | 0.4992608 | 0.7254557 | | 0.3658794 | 0.5185388 |
| zc3h13 | 0.4884555 | 0.5588838 | | 0.4990227 | 0.5747824 |
| zc3h15 | 0.3542204 | 0.8809491 | | 0.6109713 | 0.6493315 |
| zc3h18 | 0.4317119 | 0.8582968 | | 0.6677394 | 0.8237823 |
| zc3hc1 | 0.4864721 | 0.9669791 | | 0.8172836 | 0.7667514 |
| zcchc17 | 0.4516332 | 0.7469033 | | 0.6970281 | 0.5642964 |
| zfp161 | 0.4798622 | 0.5700659 | | 0.7006261 | 0.6750585 |
| zfr | 0.4966484 | 0.7724128 | | 0.6399719 | 0.8407951 |
| zgc:100869 | 0.4908939 | 0.6525695 | | 0.6892451 | 0.7240966 |
| zgc:100927 | 0.4638061 | 0.9747485 | | 0.6695069 | 0.7415402 |
| zgc:101030 | 0.3283907 | 0.5389884 | | 0.4083665 | 0.5577481 |
| zgc:101040 | 0.2207138 | 0.4112649 | | 0.2493444 | 0.1610911 |
| zgc:101100 | 0.3400354 | 0.7600708 | | 0.5303677 | 0.9890764 |
| zgc:101606 | 0.3214363 | 0.8188365 | | 0.9481293 | 0.9169791 |
| zgc:101661 | 0.4583986 | 0.6698315 | | 0.5712115 | 0.4934009 |
| zgc:101765 | 0.295824 | 0.4821996 | | 0.343362 | 0.3028105 |
| zgc:101814 | 0.449175 | 0.9200314 | | 0.8956378 | 0.8357568 |
| zgc:101819 | 0.4493635 | 0.6432418 | | 0.6591058 | 0.6960156 |
| zgc:101827 | 0.4134592 | 0.6534651 | | 0.5052467 | 0.6737097 |
| zgc:101859 | 0.1149359 | 0.4638853 | | 0.5342747 | 0.3236444 |
| zgc:101872 | 0.4054688 | 0.8940644 | | 0.8422009 | 0.6664717 |
| zgc:103459 | 0.4870074 | 0.9784894 | | 0.6322187 | 0.659752 |
| zgc:103670 | 0.4919855 | 0.6830294 | | 0.5247967 | 0.659381 |
| zgc:103692 | 0.4820026 | 0.7247945 | | 0.5129382 | 0.7745184 |
| zgc:109949 | 0.1326969 | 0.2966319 | | 0.1174012 | 0.4075392 |
| zgc:109965 | 0.1922367 | 0.1044388 | | 0.1278431 | 0.2707635 |
| zgc:109984 | 0.3397704 | 0.2285186 | | 0.1003844 | 0.2682593 |
| zgc:110045 | 0.2323224 | 0.2774433 | | 0.1262526 | 0.5668594 |
| zgc:110053 | 0.2727483 | 0.7854633 | | 0.5875883 | 0.2279542 |
| zgc:110063 | 0.4556618 | 0.4927693 | | 0.7494345 | 0.6136096 |
| zgc:110224 | 0.3098477 | 0.6470881 | | 0.7997086 | 0.6829842 |
| zgc:110251 | 0.051574 | 0.0320192 | | 0.1833392 | 0.0528629 |
| zgc:110377 | 0.3134532 | 0.3283079 | | 0.3369936 | 0.331226 |
| zgc:110682 | 0.4084263 | 0.6731377 | | 0.6614529 | 0.6621961 |
| zgc:110687 | 0.4916707 | 0.6894779 | | 0.6141838 | 0.8387545 |
| zgc:112083 | 0.3463285 | 0.5623691 | | 0.5498795 | 0.511847 |
| zgc:112178 | 0.4321631 | 0.5343532 | | 0.5164061 | 0.5435507 |
| zgc:112294 | 0.0611413 | 0.0379735 | | 0 | 0.0624985 |
| zgc:112335 | 0.3569257 | 0.6110758 | | 0.6540882 | 0.5846243 |
| zgc:112359 | 0.2780577 | 0.746762 | | 0.4772091 | 0.4484331 |
| zgc:112980 | 0.4943168 | 0.9572229 | | 0.9549511 | 0.6151771 |
| zgc:112982 | 0.3941652 | 0.8354449 | | 0.4601476 | 0.667084 |
| zgc:113019 | 0.4138675 | 0.6264009 | | 0.3983875 | 0.4343382 |
| zgc:113026 | 0.4297294 | 0.4834403 | | 0.3669461 | 0.4830642 |
| zgc:113143 | 0.3487615 | 0.5142528 | | 0.6067786 | 0.5957384 |
| zgc:113159 | 0.4165111 | 0.3439312 | | 0.4637922 | 0.6549461 |
| zgc:113209 | 0.4414598 | 0.5819068 | | 0.4099971 | 0.5845866 |
| zgc:113210 | 0.2612507 | 0.7312592 | | 0.4209427 | 0.530976 |
| zgc:113220 | 0.3982256 | 0.7379089 | | 0.4963382 | 0.5730018 |
| zgc:113383 | 0.393494 | 0.4576954 | | 0.4417859 | 0.665457 |
| zgc:113426 | 0.0470864 | 0.4678291 | | 0.3329085 | 0.337327 |
| zgc:113531 | 0.4025543 | 0.5972801 | | 0.5899455 | 0.4685566 |
| zgc:114060 | 0.4813968 | 0.5308335 | | 0.4708196 | 0.5737721 |
| zgc:114104 | 0.3093465 | 0.9389168 | | 0.6010062 | 0.6929491 |
| zgc:114130 | 0.3387545 | 0.84635 | | 0.5424843 | 0.690256 |
| zgc:114180 | 0.1868919 | 0.162561 | | 0.3585414 | 0.1524552 |
| zgc:123019 | 0.4132859 | 0.4614841 | | 0.323191 | 0.5005436 |
| zgc:136474 | 0.1985989 | 0.6580659 | | 0.4819732 | 0.5414573 |
| zgc:136552 | 0.4908236 | 0.5293013 | | 0.4077082 | 0.5504025 |
| zgc:136683 | 0.0236532 | 0.5210102 | | 0.0092355 | 0.4236177 |
| zgc:136820 | 0.1471113 | 0.2138556 | | 0.2620454 | 0.3143033 |
| zgc:136929 | 0.4976348 | 0.4041544 | | 0.4581423 | 0.3527531 |
| zgc:136936 | 0.4620619 | 0.3374671 | | 0.2758467 | 0.3930003 |
| zgc:152785 | 0.3259579 | 0.663666 | | 0.5331376 | 0.6943658 |
| zgc:152925 | 0.3930313 | 0.4508776 | | 0.6299477 | 0.5419978 |
| zgc:153115 | 0.4791149 | 0.6723381 | | 0.4051213 | 0.7674471 |
| zgc:153225 | 0.4980585 | 0.7763938 | | 0.6161629 | 0.7764946 |
| zgc:153426 | 0.4130928 | 0.390507 | | 0.3364578 | 0.3870173 |
| zgc:153935 | 0.426348 | 0.4922535 | | 0.3320647 | 0.3602441 |
| zgc:153974 | 0.318176 | 0.6914639 | | 0.4932278 | 0.5213387 |
| zgc:153980 | 0.452838 | 0.6483403 | | 0.5685707 | 0.6537214 |
| zgc:154063 | 0.4629787 | 0.6808595 | | 0.6012819 | 0.6514655 |
| zgc:154141 | 0.1657598 | 0.2830658 | | 0.2197129 | 0.2120475 |
| zgc:154169 | 0.1719706 | 0.5338384 | | 0.305534 | 0.2643704 |
| zgc:158220 | 0.479305 | 0.507791 | | 0.2621146 | 0.4130425 |
| zgc:158291 | 0.1684419 | 0.1483116 | | 0.1359844 | 0.1977228 |
| zgc:158316 | 0.3418776 | 0.5241423 | | 0.58698 | 0.5791901 |
| zgc:158350 | 0.3696148 | 0.4806039 | | 0.4364478 | 0.6002515 |
| zgc:158437 | 0.1036211 | 0.1608671 | | 0.1601976 | 0.1325473 |
| zgc:158450 | 0.4021352 | 0.8102747 | | 0.6682367 | 0.6992742 |
| zgc:158452 | 0.4942534 | 0.6608253 | | 0.6601584 | 0.6527795 |
| zgc:158610 | 0.4421933 | 0.6641923 | | 0.4406858 | 0.5581769 |
| zgc:158803 | 0.4362588 | 0.7822705 | | 0.7560271 | 0.8691433 |
| zgc:158846 | 0.0989442 | 0.4709965 | | 0.1267043 | 0.0900882 |
| zgc:161969 | 0.3504504 | 0.6971582 | | 0.4451722 | 0.521081 |
| zgc:162267 | 0.1422679 | 0.1767524 | | 0.124571 | 0.1452316 |
| zgc:162322 | 0.1858684 | 0.3163903 | | 0 | 0.1900248 |
| zgc:162351 | 0.2119384 | 0.4557411 | | 0.3978084 | 0.4574417 |
| zgc:162576 | 0.3993615 | 0.7199862 | | 0.73695 | 0.5509975 |
| zgc:162595 | 0.1591642 | 0.1482453 | | 0.2465767 | 0.2037102 |
| zgc:162612 | 0.4255791 | 0.6359509 | | 0.5514095 | 0.8774649 |
| zgc:162738 | 0.2407595 | 0.7972585 | | 0.533717 | 0.4521464 |
| zgc:162825 | 0.322504 | 0.0744647 | | 0.0583258 | 0.0909186 |
| zgc:162925 | 0.3586039 | 0.6289283 | | 0.4703754 | 0.6525396 |
| zgc:162948 | 0.3211011 | 0.5209965 | | 0.7507284 | 0.6148644 |
| zgc:162985 | 0.4854694 | 0.6283323 | | 0.7708137 | 0.6346599 |
| zgc:162999 | 0.1481297 | 0 | | 0.0522986 | 0 |
| zgc:165502 | 0.1740712 | 0.9732433 | | 0.6849164 | 0.2664427 |
| zgc:165507 | 0.1381055 | 0.0428797 | | 0.1220702 | 0 |
| zgc:165515 | 0.4211054 | 0.5498284 | | 0.5816084 | 0.4308737 |
| zgc:165525 | 0.2634221 | 0.4224908 | | 0.3900527 | 0.5174633 |
| zgc:165647 | 0.4627797 | 0.3966805 | | 0.3102858 | 0.4447825 |
| zgc:171223 | 0.3310398 | 0.5680841 | | 0.6667537 | 0.5860791 |
| zgc:171531 | 0.2561645 | 0.2369201 | | 0.2743968 | 0.645496 |
| zgc:171967 | 0.4602408 | 0.9736354 | | 0.9197185 | 0.8589632 |
| zgc:172106 | 0.0862145 | 0.1070668 | | 0.0763214 | 0.1765499 |
| zgc:173486 | 0.1690055 | 0.4547381 | | 0.1995561 | 0.2708089 |
| zgc:173506 | 0.4426635 | 0.63319 | | 0.3864096 | 0.6079831 |
| zgc:173517 | 0.1354538 | 0.3417474 | | 0.1542429 | 0.2604559 |
| zgc:173693 | 0.2937776 | 0.3784037 | | 0.1358926 | 0.3475227 |
| zgc:173915 | 0.4193929 | 0.9376391 | | 0.7318825 | 0.5537209 |
| zgc:174160 | 0.3663005 | 0.5518059 | | 0.6518748 | 0.5107722 |
| zgc:174263 | 0.4592626 | 0.6980043 | | 0.5691778 | 0.6064493 |
| zgc:175131 | 0.1376599 | 0.8119524 | | 0.2443827 | 0.2115797 |
| zgc:175222 | 0.4324252 | 0.5767031 | | 0.6149401 | 0.6796366 |
| zgc:175247 | 0.1374806 | 0.4779697 | | 0.4522897 | 0.3382429 |
| zgc:193533 | 0.0695598 | 0.1296311 | | 0.4871967 | 0.4970447 |
| zgc:193865 | 0.1423917 | 0.1326854 | | 0.1037817 | 0.2906258 |
| zgc:194221 | 0.273015 | 0.519945 | | 0.1926484 | 0.4654367 |
| zgc:194224 | 0.291517 | 0.7903406 | | 0.7739746 | 0.9187187 |
| zgc:194336 | 0.1720139 | 0.053398 | | 0.3437002 | 0.2203452 |
| zgc:194800 | 0.4516193 | 0.526534 | | 0.4315878 | 0.6777893 |
| zgc:194937 | 0.3969662 | 0.234029 | | 0.1794057 | 0.2677444 |
| zgc:55764 | 0.4583799 | 0.4757524 | | 0.4915071 | 0.6518017 |
| zgc:55870 | 0.3891907 | 0.7732143 | | 0.5974687 | 0.6095452 |
| zgc:56062 | 0.4322107 | 0.7406028 | | 0.6657459 | 0.7974051 |
| zgc:56085 | 0.1004764 | 0.1664003 | | 0.1770709 | 0.1027473 |
| zgc:56197 | 0.3879629 | 0.8029967 | | 0.7441351 | 0.8055052 |
| zgc:56235 | 0.4627686 | 0.8857513 | | 0.7936375 | 0.7464905 |
| zgc:56702 | 0.4202096 | 0.8262278 | | 0.7590725 | 0.7214349 |
| zgc:63827 | 0.4749489 | 0.7416902 | | 0.5204945 | 0.7197715 |
| zgc:63914 | 0.4394317 | 0.4893615 | | 0.5078395 | 0.6734964 |
| zgc:64065 | 0.0859106 | 0.2666756 | | 0.1910104 | 0.0880743 |
| zgc:64095 | 0.3222997 | 0.793247 | | 0.609861 | 0.5126403 |
| zgc:65870 | 0.3523002 | 0.5103009 | | 0.4010744 | 0.6865669 |
| zgc:65894 | 0.4787788 | 0.4786377 | | 0.5797756 | 0.5837628 |
| zgc:66097 | 0.2318407 | 0.183202 | | 0.1122066 | 0.1295624 |
| zgc:66125 | 0.3761639 | 0.6066397 | | 0.7729971 | 0.6765686 |
| zgc:66306 | 0.4181381 | 0.7433389 | | 0.7875768 | 0.7744012 |
| zgc:66441 | 0.3316718 | 0.5376761 | | 0.398281 | 0.5350511 |
| zgc:66448 | 0.34236 | 0.5369889 | | 0.4718589 | 0.5571928 |
| zgc:66472 | 0.4735413 | 0.8525706 | | 0.7015985 | 0.8791328 |
| zgc:73189 | 0.2661629 | 0.247866 | | 0.1182865 | 0.2728234 |
| zgc:73275 | 0.114968 | 0.1784693 | | 0.4070306 | 0.2942751 |
| zgc:73328 | 0.2625111 | 0.5463 | | 0.3247835 | 0.3860039 |
| zgc:76883 | 0.2690734 | 0.6476758 | | 0.4715818 | 0.5494781 |
| zgc:77058 | 0.4661926 | 0.3546742 | | 0.2668592 | 0.3988014 |
| zgc:77222 | 0.4961099 | 0.3344673 | | 0.3611857 | 0.3501729 |
| zgc:85936 | 0.4891852 | 0.7772012 | | 0.7233076 | 0.9889203 |
| zgc:85944 | 0.4604505 | 0.2263428 | | 0.5111892 | 0.2424029 |
| zgc:86764 | 0.4200301 | 0.6714964 | | 0.5616774 | 0.6486085 |
| zgc:91874 | 0.1682896 | 0.7052246 | | 0.2432559 | 0.2588055 |
| zgc:91890 | 0.4147339 | 0.592738 | | 0.7774707 | 0.6333223 |
| zgc:92115 | 0.0860408 | 0.367264 | | 0.6491339 | 0.5399815 |
| zgc:92172 | 0.4321492 | 0.5993323 | | 0.8516447 | 0.8392579 |
| zgc:92177 | 0.4302561 | 0.9980872 | | 0.6882238 | 0.8198884 |
| zgc:92406 | 0.2083016 | 0.3634144 | | 0.0610646 | 0.0960114 |
| zgc:92510 | 0.4932497 | 0.7894837 | | 0.8156695 | 0.8680886 |
| zgc:92799 | 0.4637066 | 0.9254596 | | 0.9006717 | 0.5668493 |
| zhx2 | 0.4684348 | 0.4869733 | | 0.437055 | 0.533796 |
| zic5 | 0.4663408 | 0.5160307 | | 0.6070774 | 0.4695667 |
| zmym4 | 0.3709086 | 0.7277061 | | 0.625302 | 0.5860713 |
| zmynd11 | 0.3984544 | 0.5551389 | | 0.4357166 | 0.6332572 |
| znf143b | 0.3695417 | 0.5488511 | | 0.3601313 | 0.5345468 |
| znf292b | 0.329037 | 0.4844453 | | 0.4746197 | 0.6055842 |
| znf346 | 0.4525144 | 0.7157755 | | 0.5347129 | 0.6578724 |
| znf395 | 0.4441224 | 0.7753031 | | 0.6741675 | 0.6972002 |
| znf574 | 0.4376275 | 0.7157273 | | 0.8080916 | 0.9311927 |
| znf592 | 0.3125102 | 0.380826 | | 0.164866 | 0.2729923 |
| znf711 | 0.3354485 | 0.4782174 | | 0.254047 | 0.3438654 |
| znf865 | 0.4480192 | 0.4709894 | | 0.393219 | 0.5362993 |
| zswim5 | 0.4554605 | 0.3748063 | | 0.3288757 | 0.4847192 |
| zw10 | 0.4908179 | 0.6365737 | | 0.5104786 | 0.4766327 |
