# Supplementary material for: Transcriptome analysis reveals a ribosome constituents disorder involved in the RPL5 downregulated zebrafish model of Diamond-Blackfan anemia
Source: BMC Med Genomics. 2016 Mar 9;9:13. doi: 10.1186/s12920-016-0174-9 (PMC4785739; doi:10.1186/s12920-016-0174-9)
Supplement: Additional file 4: Table S4. — Down-regulated genes in RPL5 MO (fold-change < 0.5 and p-value < 0.05) showed opposite regulatory trend in other DBA zebrafish models. (DOC 159 kb) [file 12920_2016_174_MOESM4_ESM.doc]

**Table S4 Down-regulated genes in RPL5 MO (fold-change < 0.5 and p-value < 0.05) showed opposite regulatory trend in other DBA zebrafish models.**

| gene_symbol | RPL5 | RPS19 | RPS24 | RPL11 |
| --- | --- | --- | --- | --- |
| LOC405768 | 0.405493 | 2.3781542 | 1.5123964 | 1.7135543 |
| LOC555692 | 0.3719911 | 1.304048 | 1.4083306 | 1.0656828 |
| aars | 0.4831263 | 1.69418 | 1.4482842 | 1.3550858 |
| aatf | 0.2961754 | 1.5001828 | 1.10972 | 1.1245943 |
| abce1 | 0.4860559 | 1.3597098 | 1.3713798 | 1.1893607 |
| abcf1 | 0.2905556 | 1.1358775 | 1.0351513 | 1.1237664 |
| actn3b | 0.4065632 | 1.550852 | 1.1239917 | 1.1934546 |
| adkb | 0.4186376 | 1.6588421 | 1.1200148 | 1.1605855 |
| asb2b | 0.4640185 | 2.3015488 | 2.2985296 | 2.360114 |
| atad3b | 0.4406857 | 1.9748146 | 1.8928612 | 1.6594794 |
| atp1a2a | 0.4784512 | 2.5865264 | 2.2346618 | 2.1164878 |
| bag3 | 0.4814867 | 1.7972518 | 1.1763857 | 2.324005 |
| bop1 | 0.4071948 | 1.5731583 | 1.3402025 | 1.35772 |
| cacna1sb | 0.3045658 | 1.8050522 | 1.5143183 | 1.4180093 |
| casq1b | 0.4630691 | 1.4623841 | 1.3401868 | 1.2144967 |
| chrna1 | 0.4950818 | 1.5061676 | 1.1356976 | 1.0367635 |
| cirh1a | 0.3129721 | 1.8206155 | 1.2404964 | 1.3795758 |
| cmn | 0.268575 | 1.5399037 | 1.9939774 | 3.4258574 |
| ctpsa | 0.3941017 | 1.3415328 | 1.0761334 | 1.0329856 |
| cx39.9 | 0.3501892 | 1.4440272 | 1.7429073 | 1.3715103 |
| dcaf13 | 0.4926218 | 1.4410541 | 1.2647783 | 1.2091741 |
| ddx18 | 0.4131433 | 1.2804301 | 1.0879763 | 1.7616151 |
| ddx27 | 0.3479468 | 1.3074454 | 1.1944046 | 1.010863 |
| ddx51 | 0.4639989 | 1.2606432 | 1.5946782 | 1.0321415 |
| ddx54 | 0.4302237 | 1.72454 | 1.5223768 | 1.4670955 |
| ddx55 | 0.4122733 | 1.1506281 | 1.0691001 | 1.1687755 |
| ddx56 | 0.4260725 | 1.6481296 | 1.4395811 | 1.461986 |
| dhtkd1 | 0.4208104 | 1.4465585 | 1.1277204 | 1.182981 |
| dhx37 | 0.3470551 | 1.6198896 | 1.281117 | 1.1160308 |
| dkc1 | 0.3367248 | 1.2908688 | 1.0369886 | 1.1103844 |
| dnajc2 | 0.3522665 | 1.3837554 | 1.1736918 | 1.1171739 |
| drg1 | 0.4708326 | 1.4249613 | 1.0763185 | 1.1218819 |
| eed | 0.45434 | 1.339787 | 1.0463069 | 1.1789115 |
| eif2b3 | 0.4272543 | 1.7661366 | 1.8667126 | 1.4838607 |
| eif2b5 | 0.4829376 | 1.2786286 | 1.1692117 | 1.1513104 |
| eif2d | 0.4230956 | 1.5076947 | 1.0620158 | 1.0282798 |
| eif5 | 0.4267829 | 1.4239271 | 1.1362835 | 1.0888378 |
| elp4 | 0.334606 | 1.5462995 | 1.42012 | 1.5610364 |
| enah | 0.3804608 | 1.6692169 | 1.2586829 | 1.3779941 |
| epb4.1l4 | 0.4908371 | 1.0574636 | 1.1959911 | 1.3370553 |
| eprs | 0.4249852 | 1.2984631 | 1.2005845 | 1.5300187 |
| esf1 | 0.2696027 | 1.4165714 | 1.2798048 | 1.1416816 |
| farsa | 0.4137565 | 1.660436 | 1.2573978 | 1.5761803 |
| fxr2 | 0.353111 | 1.8903948 | 2.0812086 | 1.758074 |
| gnl2 | 0.4302691 | 1.6591669 | 1.4553193 | 1.2891767 |
| gstcd | 0.3097103 | 1.3756089 | 1.3253081 | 1.202998 |
| gtf2h1 | 0.4012426 | 1.0438267 | 1.007157 | 1.0313024 |
| gtf3aa | 0.4276374 | 1.8140833 | 1.2978322 | 1.5574384 |
| gtpbp1l | 0.3701528 | 1.5735447 | 1.4524217 | 1.1855671 |
| gtpbp4 | 0.497339 | 1.4621012 | 1.3345339 | 1.2028381 |
| heatr1 | 0.3755889 | 2.1550242 | 1.1113007 | 1.3672447 |
| heatr3 | 0.3864346 | 1.7750063 | 1.68002 | 1.5737047 |
| hmgcra | 0.3416678 | 2.1442334 | 1.2489497 | 1.5963979 |
| hsp90aa1.1 | 0.213194 | 1.4370759 | 1.7392921 | 1.9915328 |
| hspa14 | 0.4332725 | 1.4400963 | 1.3441652 | 1.3170258 |
| hspb11 | 0.3273559 | 4.0453922 | 1.9402482 | 4.087116 |
| hspd1 | 0.4155299 | 1.1188591 | 1.0524398 | 1.0513682 |
| iars | 0.4458289 | 1.7027257 | 1.7495275 | 1.8397478 |
| imp4 | 0.4121264 | 1.5152014 | 1.354573 | 1.1674553 |
| ipo8 | 0.4956556 | 1.2227291 | 1.1235864 | 1.3240564 |
| kbtbd10b | 0.4371756 | 1.6621936 | 1.5228371 | 1.1779361 |
| klhl31 | 0.416908 | 1.8931187 | 1.8607184 | 1.3933676 |
| limch1a | 0.4468762 | 1.3776928 | 1.0924028 | 1.0641033 |
| mak16 | 0.4119732 | 1.3334793 | 1.012716 | 1.0261709 |
| mapkapk3 | 0.4695351 | 1.9459398 | 1.291134 | 1.2987543 |
| mars | 0.399981 | 1.2404775 | 1.2309691 | 1.2551119 |
| matn3a | 0.2927609 | 1.5664601 | 1.6873524 | 1.5964844 |
| mrps30 | 0.4663292 | 1.3628588 | 1.2542656 | 1.1940395 |
| mthfr | 0.4622434 | 1.0812023 | 1.4034753 | 1.6029041 |
| mtr | 0.3376208 | 1.152102 | 1.3191439 | 1.1511479 |
| c-mybbp1a | 0.3476102 | 1.092835 | 1.0221351 | 1.1609804 |
| myca | 0.4227839 | 1.1578403 | 1.0934181 | 1.0479072 |
| myom1a | 0.4037853 | 1.1534776 | 1.0519846 | 1.0114273 |
| myoz2a | 0.4291843 | 1.4565258 | 1.2526688 | 1.2318204 |
| naa15a | 0.4117806 | 1.3393879 | 1.1244216 | 1.0728247 |
| ncl1 | 0.4807842 | 1.092136 | 1.1529655 | 1.3129453 |
| nexn | 0.3878395 | 1.69238 | 1.3426943 | 1.7781405 |
| nle1 | 0.4170378 | 1.5084294 | 1.4841647 | 1.3562769 |
| noa1 | 0.4400835 | 1.4870857 | 1.3530989 | 1.0566548 |
| noc3l | 0.3827053 | 1.3450362 | 1.2824449 | 1.0478446 |
| noc4l | 0.4647852 | 1.5287352 | 1.4856784 | 1.3460328 |
| nol6 | 0.3077024 | 1.224911 | 1.0628853 | 1.0085768 |
| nolc1 | 0.4244161 | 1.4243899 | 1.0744666 | 1.1541786 |
| nop56 | 0.4686969 | 1.3019917 | 1.1024842 | 1.1955066 |
| npm1a | 0.3372064 | 1.5402695 | 1.0868543 | 1.1060203 |
| nsun4 | 0.384039 | 1.4309391 | 1.3389374 | 1.0510114 |
| nup54 | 0.4182431 | 1.4900856 | 1.1073174 | 1.0609979 |
| pa2g4a | 0.4465065 | 1.3271393 | 1.183999 | 1.0545215 |
| pes | 0.3230468 | 1.4562905 | 1.1310621 | 1.2496585 |
| pinx1 | 0.4417326 | 1.3962136 | 1.1212596 | 1.1066761 |
| polr1e | 0.4237662 | 1.9163117 | 1.4621921 | 1.3074407 |
| polr3e | 0.4888704 | 1.2792325 | 1.1462777 | 1.2357029 |
| polr3gla | 0.3781718 | 2.4251179 | 1.4963699 | 1.3792466 |
| ppan | 0.464439 | 1.6970678 | 1.1692525 | 1.3744357 |
| prmt5 | 0.3636227 | 1.3770946 | 1.2238754 | 1.0932005 |
| ptcd3 | 0.4097914 | 1.7817995 | 1.4166594 | 1.1964601 |
| ptpn12 | 0.4584298 | 1.4524175 | 1.1023853 | 1.212898 |
| pus7 | 0.4740568 | 2.5529155 | 1.6331495 | 2.0024144 |
| qars | 0.4509603 | 2.4862574 | 1.8553271 | 2.1106975 |
| qdprb1 | 0.4746269 | 1.7543476 | 1.4979304 | 1.3723718 |
| qtrt1 | 0.4244494 | 1.9184971 | 1.6597192 | 1.6435116 |
| rbbp9 | 0.4824858 | 1.5233787 | 1.2163974 | 1.1895445 |
| rbfox1l | 0.3682899 | 1.8576458 | 1.7775066 | 1.217097 |
| rbm19 | 0.3588888 | 1.3116499 | 1.0947393 | 1.0446413 |
| rbm34 | 0.3538906 | 1.4208529 | 1.2055133 | 1.291488 |
| riok2 | 0.4729464 | 1.3814519 | 1.0796955 | 1.0197991 |
| rpf2 | 0.4782776 | 1.8628102 | 1.6201549 | 1.4732514 |
| rpl5a | 0.2108875 | 1.2496475 | 1.593182 | 1.4320715 |
| rrp12 | 0.3425906 | 1.6975426 | 1.3887331 | 1.2721402 |
| rrp9 | 0.4372481 | 1.5181431 | 1.1487671 | 1.3206409 |
| sdad1 | 0.3908929 | 1.4879954 | 1.2614099 | 1.3994636 |
| si:ch211-261f7.2 | 0.3498954 | 2.0236359 | 1.4406181 | 1.2270104 |
| si:dkeyp-84a8.8 | 0.455697 | 1.6381038 | 1.3714167 | 1.5411603 |
| smyd1a | 0.4872607 | 1.6485094 | 1.418203 | 1.4330311 |
| smyd1b | 0.4563457 | 2.5127569 | 2.4876329 | 2.1388402 |
| smyd2b | 0.3454692 | 1.1754749 | 1.1148337 | 1.1510432 |
| smyd5 | 0.4474248 | 2.1320244 | 1.6951957 | 1.6571682 |
| stac3 | 0.4878288 | 2.1974337 | 2.0622407 | 1.2101111 |
| tagln | 0.4933821 | 3.4804855 | 1.838482 | 1.1827882 |
| tars | 0.3711212 | 1.2722206 | 1.2030247 | 1.1407407 |
| tbl2 | 0.3490316 | 1.0339495 | 1.2445165 | 1.0126123 |
| tgm2a | 0.4110702 | 1.7150672 | 2.8337348 | 2.0136413 |
| timm50 | 0.4518644 | 2.868287 | 1.8388454 | 1.2980935 |
| tmx2a | 0.444269 | 2.7591682 | 2.1435787 | 2.7190332 |
| trim55a | 0.3841532 | 2.1583193 | 2.2989167 | 1.8376698 |
| trim55b | 0.3697208 | 3.0677515 | 1.9451832 | 2.1400307 |
| trim63 | 0.3941278 | 1.3872255 | 1.0296097 | 1.286151 |
| tsr1 | 0.4013859 | 1.9167538 | 1.6493935 | 1.5623401 |
| ttc4 | 0.3845196 | 1.5491479 | 1.0095747 | 1.0058786 |
| twf2 | 0.3994913 | 1.2897006 | 1.1620317 | 1.1242202 |
| txlnbb | 0.2494878 | 1.8125901 | 1.5664286 | 1.5459397 |
| tyw1 | 0.4900013 | 1.0372147 | 1.0059155 | 1.0810818 |
| unc45b | 0.2502968 | 2.2236378 | 2.0180244 | 2.6402325 |
| utp18 | 0.3846925 | 1.8328052 | 1.6308245 | 1.5753198 |
| utp6 | 0.4380932 | 1.4648201 | 1.0718276 | 1.0833929 |
| vgll2b | 0.3045293 | 1.8311163 | 1.4318032 | 1.5845688 |
| vtg1 | 0.2662093 | 3.9917183 | 1.4046062 | 1.4836588 |
| wbscr22 | 0.4515455 | 1.7419945 | 1.158117 | 1.2683824 |
| wdr12 | 0.4462069 | 1.5996234 | 1.3391169 | 1.1716334 |
| wdr3 | 0.3914901 | 1.7171993 | 1.2266691 | 1.2445871 |
| wdr36 | 0.4048115 | 1.4605113 | 1.0526327 | 1.0534269 |
| wdr43 | 0.3627078 | 1.5341312 | 1.3430033 | 1.2342077 |
| wdr74 | 0.4683219 | 1.2660786 | 1.1119455 | 1.1667325 |
| wdr75 | 0.3956823 | 1.8733512 | 1.5439961 | 1.4414501 |
| wu:fc61g08 | 0.4259997 | 1.6123802 | 1.2275195 | 1.3236201 |
| yars | 0.3782367 | 1.7467423 | 1.3541798 | 1.3913718 |
| zgc:103638 | 0.1505068 | 3.4477649 | 2.7635109 | 3.1916051 |
| zgc:109995 | 0.4200198 | 2.0235244 | 1.9742616 | 1.3724999 |
| zgc:110388 | 0.4797016 | 1.563849 | 1.1226601 | 1.152843 |
| zgc:110848 | 0.1815241 | 2.2585123 | 1.4066112 | 1.2348757 |
| zgc:112350 | 0.4202992 | 1.7637063 | 1.571332 | 2.5417238 |
| zgc:113232 | 0.3659959 | 1.7190684 | 3.6675204 | 2.1391519 |
| zgc:113358 | 0.3950529 | 1.8402504 | 1.4092341 | 1.4087129 |
| zgc:152769 | 0.3307287 | 1.2779022 | 1.365427 | 1.0304075 |
| zgc:153172 | 0.4862051 | 1.3408434 | 1.1369041 | 1.0053719 |
| zgc:153635 | 0.4022541 | 1.4984889 | 1.1703614 | 1.1179455 |
| zgc:153989 | 0.4383835 | 2.4386883 | 1.8754991 | 1.4435736 |
| zgc:171476 | 0.3264624 | 1.5584087 | 1.5921554 | 1.2791925 |
| zgc:172158 | 0.3558043 | 1.5463575 | 1.770909 | 1.1651739 |
| zgc:56683 | 0.4753065 | 1.7211644 | 1.2168361 | 1.0901344 |
| zgc:63779 | 0.4399963 | 1.4522787 | 1.6676853 | 1.4537526 |
| zgc:76871 | 0.4459672 | 1.7147364 | 1.803274 | 2.0812036 |
| zgc:77221 | 0.4603978 | 1.6787115 | 1.6381807 | 1.4806661 |
| zgc:86709 | 0.4906556 | 4.1058818 | 2.1860608 | 2.7134006 |
| zgc:92006 | 0.4565979 | 1.2802486 | 1.1559068 | 1.3660534 |
| zgc:92429 | 0.3413429 | 1.4098519 | 1.4510618 | 1.425659 |
| zgc:92518 | 0.3769482 | 1.085583 | 1.0092697 | 1.01912 |
| znf330 | 0.4303883 | 1.8231567 | 1.3609579 | 1.3643873 |
