# Supplementary material for: Transcriptome analysis reveals a ribosome constituents disorder involved in the RPL5 downregulated zebrafish model of Diamond-Blackfan anemia
Source: BMC Med Genomics. 2016 Mar 9;9:13. doi: 10.1186/s12920-016-0174-9 (PMC4785739; doi:10.1186/s12920-016-0174-9)
Supplement: Additional file 5: Table S5. — LncRNAs commonly regulated (fold-change > 2 and p-value < 0.05) in DBA zebrafish models and expression patterns correlated with more than 3000 genes (DOC 32 kb) [file 12920_2016_174_MOESM5_ESM.doc]

**Table S5 LncRNAs commonly regulated (fold-change > 2 and p-value < 0.05) in DBA zebrafish models and expression patterns correlated with more than 3000 genes**

| **Locus** | **FPKM in Control** | **FPKM in RPL11** | **FPKM in RPL5** | **FPKM in RPS19** | **FPKM in RPS24** | **Number of correlated genes** |
| --- | --- | --- | --- | --- | --- | --- |
| chr2:40268840-40271962 | 2.91352 | 1.27309 | 2.54751 | 1.19642 | 0.836629 | 3775 |
| chr9:20828527-20830579 | 0.067476 | 1.22653 | 0.579036 | 1.81806 | 1.79742 | 3755 |
| chr4:48079733-48080742 | 2.24734 | 0.542896 | 1.86791 | 0.343783 | 0.271995 | 3732 |
| chr2:23611814-23614038 | 2.56123 | 1.04127 | 2.00911 | 0.87215 | 0.660505 | 3693 |
| chr21:26686909-26688701 | 0.826776 | 0.125121 | 0.819824 | 0.182092 | 0.088972 | 3608 |
| chr6:14971188-14972634 | 1.56389 | 0.37702 | 1.10996 | 0.26897 | 0.286638 | 3501 |
| chr7:74751006-74752902 | 0.826117 | 0.19529 | 0.657966 | 0.170495 | 0.166719 | 3493 |
| chr22:2890140-2890961 | 0 | 0.34114 | 0.106916 | 0.926627 | 0.838533 | 3414 |
| chr24:11807202-11808164 | 3.31437 | 8.21007 | 3.9583 | 8.18349 | 15.2278 | 3277 |
| chr8:38391146-38392280 | 1.25089 | 0.219167 | 0.685157 | 0.136994 | 0.077501 | 3209 |
