# Supplementary material for: Transcriptome analysis reveals a ribosome constituents disorder involved in the RPL5 downregulated zebrafish model of Diamond-Blackfan anemia
Source: BMC Med Genomics. 2016 Mar 9;9:13. doi: 10.1186/s12920-016-0174-9 (PMC4785739; doi:10.1186/s12920-016-0174-9)
Supplement: Additional file 6: Table S6. — The primers designed for real-time PCR (DOC 33 kb) [file 12920_2016_174_MOESM6_ESM.doc]

**Table S6** The primers designed for real-time PCR

| **Genes/miRNAs** | **Primers** |
| --- | --- |
| cirh1a | forward primer: caggcaatcaacaaggaaca  reverse primer: gaagggtgccaggatagaga |
| noc2l | forward primer: ttcaattccctcgtcctgtt  reverse primer: tctgccacctggtacttgaa |
| abce1 | forward primer: ggagaaggaaaccacacaca  reverse primer: ccaggattttgagagcagtg |
| tars | forward primer: cgagtaatgatttcccctgt  reverse primer: tcctgcacttgaacttgttg |
| nol6 | forward primer: gggcctgctactcctcatta  reverse primer: ggccacaccctctctaaaag |
| β-Actin | forward primer: agatcaagatcattgctccccc  reverse primer: ggccatttaaggtggcaaca |
| dre-miR-10a | gccaaattcgtgtcttggggaata |
| dre-miR-722 | gcttttttgcagaaacgtttcagatt |
| dre-miR-737 | gcgcaatcaaaacctaaagaaaata |
| dre-miR-155 | gcttaatgctaatcgtgatagggg |
| dre-miR-223 | gctgtcagtttgtcaaatacccc |
| dre-miR-142a-3p | gctgtagtgtttcctactttatgga |
